# PRRX1 silencing is required for metastatic outgrowth in melanoma and is an independent prognostic of reduced survival in patients

**DOI:** 10.1002/1878-0261.13688

**Published:** 2024-07-08

**Authors:** Josep R. Ferreres, Antònia Vinyals, Rafael Campos‐Martin, Roderic Espín, Sebastian Podlipnik, Raquel Ramos, Esther Bertran, Cristina Carrera, Joaquim Marcoval, Josep Malvehy, Isabel Fabregat, Susana Puig, Àngels Fabra

**Affiliations:** ^1^ TGF‐β and Cancer Group, Oncobell Program, Bellvitge Biomedical Research Institute (IDIBELL) Hospital Duran i Reynals Barcelona Spain; ^2^ Centro de Investigaciones Biomédicas en Red de Enfermedades Hepáticas y Digestivas (CIBEREHD) ISCIII Instituto de Salud Carlos III Madrid Spain; ^3^ Dermatology Service, IDIBELL Hospital Universitari de Bellvitge Barcelona Spain; ^4^ Division of Neurogenetics and Molecular Psychiatry, Department of Psychiatry and Psychotherapy University of Cologne Germany; ^5^ Program Against Cancer Therapeutic Resistance (ProCURE) Catalan Institute of Oncology (ICO), Oncobell Program (IDIBELL) Barcelona Spain; ^6^ Dermatology Department, Melanoma Unit, Hospital Clínic IDIBAPS & University of Barcelona Spain; ^7^ Centro de Investigaciones Biomédicas en Red de Enfermedades Raras (CIBERER) ISCIII Instituto de Salud Carlos III Madrid Spain

**Keywords:** BRAF, EMT‐MET, melanoma, phenotypic plasticity, prognostic of survival, *PRRX1*

## Abstract

Paired related homeobox 1 (*PRRX1*) is an inducer of epithelial‐to‐mesenchymal transition (EMT) in different types of cancer cells. We detected low *PRRX1* expression in nevus but increased levels in primary human melanoma and cell lines carrying the *BRAF* ^*V600E*^ mutation. High expression of *PRRX1* correlates with invasiveness and enrichment of genes belonging to the EMT programme. Conversely, we found that loss of *PRRX1* in metastatic samples is an independent prognostic predictor of poor survival for melanoma patients. Here, we show that stable depletion of PRRX1 improves the growth of melanoma xenografts and increases the number of distant spontaneous metastases, compared to controls. We provide evidence that loss of *PRRX1* counteracts the EMT phenotype, impairing the expression of other EMT‐related transcription factors, causing dysregulation of the ERK and signal transducer and activator of transcription 3 (STAT3) signaling pathways, and abrogating the invasive and migratory properties of melanoma cells while triggering the up‐regulation of proliferative/melanocytic genes and the expression of the neural‐crest‐like markers nerve growth factor receptor (NGFR; also known as neurotrophin receptor p75NTR) and neural cell adhesion molecule L1 (L1CAM). Overall, our results indicate that loss of PRRX1 triggers a switch in the invasive programme, and cells de‐differentiate towards a neural crest stem cell (NCSC)‐like phenotype that accounts for the metastatic aggressiveness.

## Graphical abstract

*PRRX1* expression in primary melanomas maintains the mesenchymal‐like phenotype, but its silencing is required for the outgrowth of metastases. We show that *PRRX1* silencing in melanoma cells causes a loss of mesenchymal features and triggers a phenotypic switch towards a differentiation programme. Low *PRRX1* expression in metastatic samples is an independent prognostic predictor of melanoma patients' overall survival (OS).

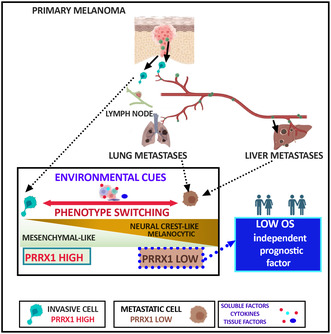

**Abbreviations**
AUC: area under the curve
BRAF: B‐Raf proto‐oncogene, serine/threonine kinase
CSC: cancer stem cell
CTC: circulating tumor cells
EMT: epithelial‐to mesenchymal transition
ERK: extracellular signal‐regulated kinase
ESCC: esophagus squamous cell carcinoma
FCS: foetal calf serum
FN1: fibronectin 1
GEO: Gene Expression Omnibus
GSEA: gene set enrichment analysis
HCC: hepatocellular carcinoma
MAPK: mitogen‐activated protein kinases
MCL2: myosin light chain 2
MEK: mitogen‐activated protein kinase
MET: mesenchymal‐to‐epithelial transition
MITF: microphthalmia‐associated transcription factor
MMP: matrix metalloproteinase
NANOG: NANOG homeobox
NCSC‐like: neural crest stem cell‐like
NGFR: nerve growth factor receptor
NSG mice: NOD.Cg‐*Prkdc* ^scid^ Il2rg^tm1Wjl^/SzJ
OS: overall survival
PRRX1: paired related homeobox1
SKCM‐TCGA: Skin Cutaneous Melanoma Cancer Genome Atlas database (TCGA)
SLUG: Snail family transcriptional repressor 2
SNAIL: Snail family transcriptional repressor 1
SOX: SRY‐box
SSM: superficial spreading melanoma
TF: transcription factor
TGF‐β: transforming growth factor beta
TWIST1: Twist family bHLH transcription factor 1
ZEB1: zinc finger E‐box binding homeobox 1
ZEB2: zinc finger E‐box binding homeobox 2

## 1 Introduction

Malignant melanoma is one of the most aggressive forms of skin cancer because of its high metastatic potential and resistance to treatments. Acquisition of *BRAF* or *NRAS* mutations is a recurrent event in melanoma and triggers the activation of MAPK, leading to constitutive ERK signaling and enhanced proliferation and survival [1, 2].

Moreover, melanoma progression relies on the plasticity of tumor cells, which renders them able to adapt to different microenvironments through the induction of changes in their phenotype in response to extracellular signals [3]. Most of these changes are executed by a non‐genetic process known as Epithelial‐Mesenchymal Transition (EMT) in carcinomas [4, 5, 6, 7], which is finely regulated by the SNAIL, ZEB, TWIST, FOXC2, and PRRX families of transcription factors (TF) [8, 9]. These TF share the ability to repress epithelial genes and directly or indirectly activate genes associated with a mesenchymal phenotype, affording invasive and migratory properties to tumor cells [10], in a context and cell‐type‐specific manner [11]. In non‐epithelial cancers such as melanoma, a similar process of cellular plasticity contributes to transitions between proliferative/differentiated (ZEB2^high^/SLUG^high^) and invasive/mesenchymal‐like (ZEB1^high^/TWIST1^high^) phenotypes [12, 13], engaged by the loss of MITF expression [14]. The transitions, originally described by Hoek et al., as “phenotypic switching” [15], occur back and forth between both phenotypes in response to external cues [16, 17].

Recently, based on melanoma expression at the single‐cell level, the phenotype‐switching model has been redefined into four distinct stepwise stages: melanocytic, transitory, neural crest‐like, and undifferentiated [18, 19, 20]. The neural crest‐like subtype was defined by its enrichment of neural crest‐related genes such as NGFR, whose expression during melanocyte development supports melanoma initiation, progression, metastasis, and immune‐chemoresistance to the therapeutic agents [21, 22, 23].

PRRX1 is a TF belonging to the paired homeobox family [24] that regulates the EMT process through TGFß‐, Notch‐, and the activation of Wnt/ßcatenin‐signaling pathways [24, 25], and confers migratory and invasive properties characteristic of the mesenchymal phenotype. PRRX1 overexpression is found to be associated with poor prognosis in epithelial cancers such as pancreas, head–neck squamous cell carcinoma (SCC), gastric, liver, and colon [26, 27, 28, 29, 30], as well as in nonepithelial cancers, and in uveal and cutaneous melanoma [31, 32].

This contrasts with the findings in HCC, breast, and lung‐SCC, in which PRRX1 downregulation is a risk factor for early recurrence and reduced overall survival [33, 34, 35, 36].

Nevertheless, the biological functions and clinical significance of PRRX1 expression in human cutaneous melanoma have not been studied. Here, we show that *PRRX1* expression is dependent on MAPK‐activation and its levels correlate with “invasiveness”. Conversely, low *PRRX1* expression in metastatic samples is an independent prognostic predictor of reduced survival in melanoma patients.

In this work, we sought to decipher the key phenotypic changes triggered by loss of PRRX1 expression that account for the increased metastatic potential at distant sites. We show that stable silencing of *PRRX1* in melanoma cells counteracts the invasiveness associated with the EMT programme, impairing the expression of other EMT‐TFs while driving a phenotype switch towards a dedifferentiation programme, such as the NCSC‐like. This new melanoma cell state may favour cell proliferation and metastasis in orthotopic xenografts and cause poor survival in melanoma patients.

## 2 Materials and methods

### 2.1 Human melanoma samples

Three sets of samples were included: (a) 50 fresh‐frozen non‐invasive primary tumors with a Breslow index < 4 mm (cohort I); (b) 44 consecutive fresh‐frozen primary melanoma and 37 metastasis samples derived from patients, independently of disease stage (cohort II); (c) 103 primary melanomas and 367 metastases included in the SKCM‐TCGA database (cohort III). Clinical and histological characteristics are provided in Tables S1 and S2. Samples from cohorts I and II were collected at the IDIBELL‐HUB and the Melanoma Unit‐Hospital Clinic Barcelona, respectively.

### 2.2 Cell lines

Human melanoma cell lines WM793 (CVCL_8787), SK‐Mel 131 (CVCL_6081), MeWo (CVCL_0445), WM1366 (CVCL_6789), SK‐Mel173 (CVCL_6090), SK‐Mel 147(CVCL_3876), WM115 (CVCL_0040) and WM 1552c (CVCL_6472) were purchased from the ATCC, Manassas, VA, USA; the TRP cell line was derived from a patient at the Melanoma Unit‐Hospital Clinic, Barcelona. All the cell lines were used in our previous studies [37]. Cell line A375MM was a kind gift from M. Nakajima (Tokyo, Japan), who derived the cell line from A375P (CVCL_6233) following an identical selection procedure as described previously [38]. Authentication was ensured by validating the cell lines using the STR‐based method and genemapper v3.7 software (Applied Biosystems, Waltham, MA, USA).

*PRRX1* knockdown was performed in WM 793, and A375 MM cells that overexpressed PRRX1 and SK‐Mel 131 cells that expressed low levels of PRRX1. Cells were cultured in DMEM: F12 medium (1 : 1) supplemented with 10% inactivated FCS (Life Technologies‐Thermo Fisher Sci., Waltham, MA, USA) and maintained at 37 °C in a humidified atmosphere with 5% CO_2_. Cells were routinely tested for Mycoplasma contamination. All studies were performed within a few passages after thawing the cells.

Where indicated, cells were incubated with either ERK‐inhibitor PD 98059 (1 μmol·L^−1^) or MEK1/2 inhibitor UO126 (5 μmol·L^−1^) from Calbiochem (Darmstadt, Germany) for 48 h.

### 2.3 Viral production and cell infections

Lentiviral plasmids used to silence PRRX1 (sh*PRRX1*:RHS3979‐9588052/201751761) and TWIST1 (sh *TWIST1* RHS3979‐9587949/201751656) were purchased from Thermo Scientific Open Biosystems, GE (Lafayette, CO, USA). The empty pLKO.1 puro vector was used as a control. pLenti CMV GFP Addgene#17448 was used as a reporter of EGFP expression (Addgene, Watertown, MA, USA), summarized in Table S3. Lentivirus was packaged as described previously [37]. Supernatants containing viral particles were used for viral infections carried out on exponentially growing cultures in the presence of 8 μg·mL^−1^ polybrene. Two consecutive rounds of infection were performed, and cells were selected with 2 μg·mL^−1^ puromycin (Life Technologies) for 2 weeks. Best‐silenced clones were chosen and at least two were used for functional studies in each cell line.

### 2.4 Cell proliferation

Cells (5 × 10^3^) were seeded in a 96‐well plate and cultured overnight in complete media. The next day cells were transferred to 2.5% FCS and cultured under the described conditions until reaching the experimental end‐point. The number of attached cells was estimated by the crystal violet method, and measured by Absorvance (A^570 nm^).

### 2.5 Spheroid formation assay

Spheroid (mø) cultures were performed as described in Ocaña et al. [36]. Briefly, 3 × 10^3^ single cells were plated in 6‐well plates previously coated with poly‐HEMA (Sigma‐Aldrich, St Louis, MO, USA) to avoid cell attachment. Cells were grown in serum‐free DMEM/Glutamax media (Life Technologies‐Thermo Fisher Sci.), and supplemented with B27 without vitamin A (1 : 50) (Life Technologies‐Thermo Fisher Sci.), 4 μm insulin (Sigma‐Aldrich), 20 ng·mL^−1^ human basic fibroblast growth factor (bFGF; PeproTech, Cranbury, NJ, USA), 2 ng·mL^−1^ recombinant human epidermal growth factor (hrEGF; Sigma‐Aldrich), 4 μg·mL^−1^ sodium heparin salt from porcine intestinal mucosa (Sigma‐Aldrich), and 10 ng·μL^−1^ Leukemia Inhibitory Factor (LIF 1010) (Millipore, Burlington, MA, USA). Primary spheroids were collected after 10 days, enzymatically dissociated, and sieved through a 40 μm pore cell strainer (Sarstedt AG &Co, Numbrecht, Germany). The single‐cell suspension was used for further replating and the tertiary spheroids were analyzed.

### 2.6 Cell culture on thick layers of collagen I

Fibrillar bovine collagen I (5005; PureCol, Advanced BioMatrix, San Diego, CA, USA) was prepared at 1.7 mg·mL^−1^ in DMEM as described previously [39]. After polymerization (4 h–37 °C, 10% CO_2_) cells were seeded on top in media containing 10% FCS and fixed after 24 h in culture. Where indicated, cells were incubated with 2.5 μm Blebbistatin (a Myosin II ATPase inhibitor) from Calbiochem for 24 h. Immunostaining was performed using the antibodies summarized in Table S4. For imaging, collagen gels were transferred to glass‐bottom dishes and visualized on a Zeiss LSM 510 Meta confocal microscope (Carl Zeiss, Cambridge, UK) with C‐Apochromat Å ~ 40/1.2 numerical aperture and zen software (Carl Zeiss). Confocal Z‐slice images were analyzed using imagej software (U. S. National Institutes of Health, Bethesda, MA, USA).

### 2.7 Migration and invasion assays

Cell migration was examined by Transwell assay using a 6.5 mm diameter and an 8 μm pore polycarbonate membrane. Briefly, 2 × 10^5^ cells in serum‐free media were seeded in triplicate on the upper chamber and media containing 10% FCS was used as a chemoattractant in the lower compartment. Cells were allowed to migrate overnight at 37 °C. Then, the inserts were collected the non‐migrating cells on the upper surface of the filter were wiped with a cotton swap, and the migrated cells on the lower face of the polycarbonate filters were fixed and stained with crystal violet. Quantification of migrated cells was carried out by absorbance (A^570 nm^) of stained filters and expressed relative to controls.

The invasive potential of melanoma spheroids was analyzed using the hanging drop method, as previously described [40]. Briefly, spheroids were resuspended in collagen I solution (1.7 mg·mL^−1^) and incubated in media containing 10%FCS. Phase‐contrast images were taken on day four after seeding.

### 2.8 Gelatin zymography

Gelatin zymography was performed in the serum‐free media of exponential cultures as described previously [41]. The gelatinolytic activities were detected in the gelatin‐embedded gel as clear bands against a blue background.

### 2.9 Immunoblot analysis

Western blotting was performed on whole‐cell extracts by lysing cells in RIPA buffer as previously described [41]. The blots were probed with primary antibodies (listed in Table S4) and detected using either horseradish peroxidase‐linked anti‐mouse or anti‐rabbit conjugates, as appropriate, from Dako (Glostrup, Denmark) and visualized using the Immobilon Western Chemiluminescent HRP Substrate (Millipore) following the manufacturer's instructions.

### 2.10 Immunohistochemistry

Immunohistochemical studies were performed from paraffin‐embedded tissues as described previously [37]. Antigen–antibody complexes were detected with Super Sensitive Link‐Label IHC (BioGenex Laboratories, Fremont, CA, USA) and developed with the ImmPACT NovaRED system (Vector Laboratories, Burlingame, CA, USA). The sections were counterstained with hematoxylin and images were acquired using a Nikon ECLIPSE 80i microscope. The list of antibodies used is provided in Table S4.

### 2.11 Orthotopic tumor growth and spontaneous metastasis

NOD.Cg‐*Prkdc* ^scid^ Il2rg^tm1Wjl^/SzJ (NSG) male mice were purchased from Charles River (Charles River Laboratories, Les Oncins, France) and kept in filter top cages at 22 °C with 60% humidity. Food and water were provided *ad libitum*.

For orthotopic implantations, 2 × 10^6^ cells were intradermally injected into 8‐week‐old NSG mice and monitored twice a week. Tumor volumes were calculated by measuring two orthogonal diameters (*L* and *W*) and the *V* = (*L* × W^2^)/2 formula. Tumors (100 mm^3^) were excised under anesthesia and mice were kept alive until the experimental end‐point (once the mouse first presented respiratory distress or weight loss > 15% of their body weight) and were then sacrificed. Metastatic dissemination was identified by gross and histologic examination. Liver and lung metastases were visualized and scored under a Leica ST microscope. Primary tumors and metastasis were fixed in formalin and embedded in paraffin for further histological studies.

### 2.12 Tumor cell extravasation

Briefly, 2 × 10^6^ EGFP‐A375MM mock or ‐sh*PRRX1* cells were inoculated into the tail vein of NSG mice. After 24–30 h, mice were injected (i.v.) with Texas Red‐conjugated lectin (TL‐1176‐1; Vector Laboratories) to label the lung vasculature and sacrificed 60 min later. For technical controls, mice were sacrificed 1 h after injection. Lungs were processed as described earlier [36] and the number of EGFP^+^ cells was counted (10 fields/section) under observation in a Nikon Eclipse 80i epifluorescence microscope (20×). Representative images were processed using image j software.

### 2.13 Genomic DNA and RNA extraction

DNA extraction from Fresh‐Frozen tumors was carried out using the QIAamp DNA mini kit (Qiagen, Valencia, CA, USA) as per the manufacturer's instructions. Total RNA was extracted from frozen samples and cultured cells using the TRI Reagent (Sigma‐Aldrich).

### 2.14 *BRAF* and *NRAS* characterization

*BRAF* and *NRAS* mutations were analyzed in the primary melanomas and metastases from cohorts I and II by PCR and direct sequencing. The primers used for *BRAF* exons 11 and 15 and *NRAS* exons 2 and 3 were described previously [37].

### 2.15 Gene expression analysis and gene set enrichment analysis (GSEA)

RNA Interference was performed in transient transfections with siBRAF and negative controls (scrambled) using Lipofectamine RNAiMAX following the manufacturer's instructions. All reagents were purchased from Life Technologies (Carlsbad, CA, USA). The siBRAF targeted sequence (5′‐GGUCUAGCUACAGAGAAAUCUCGAU‐3′), and scrambled controls were previously validated [37]. Cells were harvested 60 h after transfection and RNA was isolated. Stable transduced cells were selected as described above and collected at the indicated times after platting.

Reverse transcription was performed with the First Strand cDNA Synthesis kit (Life Technologies) using random hexamer primers. Quantitative RT‐PCR was performed in an LC 480 machine using the SYBR Green Mastermix from Roche Life Science (Mannheim, Germany). Primers listed in Table S5, were purchased from Life Technologies. All samples were normalized to the RP*L32* internal control, and fold changes were calculated through relative quantification ($2^{-\Delta \Delta C_{t}}$).

Data from the GSE65904 [42], GSE22155 [43], GSE50509 and GSE116237 [44, 45] datasets, were retrieved from the Gene Expression Omnibus (GEO) [46] using the Bioconductor package GEOquery [47]. Simultaneously, data generated by the TCGA Research Network was acquired at the webpage (https://www.cancer.gov/TCGA‐SKCM).

The signature, characterized by “Invasiveness”, consists of 200 genes specified as “Halmark_Epithelial_Mesenchymal_Transition” in GSEA_MSigDB M5930 and those defined as “invasive” in recent literature [16, 17] (Table S6). Signature scores were derived using the Single Sample Gene Set Expression Analysis (ssGSEA) algorithm, implemented through the Gene Set Variation Analysis (gsva) [48] software (version 1.36.2). TCGA‐SKCM samples were classified according to their invasiveness (ssGSEA scores) using the median as the threshold. Subsequently, a *t*‐test was conducted to compare the two conditions. The resulting *t*‐statistic was utilized to establish a gene ranking, which was then applied in the Gene set enrichment analysis (pre‐ranked GSEA “invasive vs non‐invasive”) [49]. Similar analysis was performed according to *PRRX1* gene expression using the median as the threshold and performed pre‐ranked GSA comparing high *PRRX1* expression versus low expression.

The gene signatures included known pathways from KEGG and REACTOME; functional terms for GO; curated signatures from MsigDB v4 (http://www.broadinstitute.org/gsea/msigdb/genesets) and the hallmark Gene set collection [50].

In the non‐preranked GSEA analysis genes were systematically ranked based on their Pearson correlation with *PRRX1* gene expression. Enrichment computations were performed through 1000 permutations. Gene sets with an FDR‐value < 0.05 were considered significant.

### 2.16 Statistical analysis

Median *PRRX1* expression levels in cohorts II and III (SKCM‐TCGA) were used to divide patients into high‐ and low‐expression groups. The data sets were further segmented into metastatic and primary tumor samples. Kaplan–Meier analysis was used to compare high‐ and low‐expression patients in each group by the log‐rank test. The *PRRX1* expression group's hazard ratios (HR) and confidence intervals (CI) were estimated using multivariate Cox regression adjusted by the Breslow and Clark scores. Computations were carried out using the r software (version 4.2.2) (R Foundation for Statistical Computing, Vienna, Austria). The COX analysis was performed with the “survival” package (version 3.3), Kaplan–Meier evaluation with the “survminer” package (version 0.4.9), and “time‐dependent analysis” with the “timeROC” package (version 0.4). graphpad prism software 9.0 (GraphPad, La Jolla, CA, USA) was used for the rest of the statistical analysis.

### 2.17 Ethics statement

Clinical samples from cohorts I and II were collected from February 1997 to August 2019, at the IDIBELL‐HUB and the Melanoma Unit‐Hospital Clinic Barcelona, respectively. An EUS/ERCP database maintained for this period was retrospectively reviewed. Studies were approved by our institutional ethics committee (CEIC) of IDIBELL‐HUB (Reference PR355/13) and the Hospital Clinic (Reference HCB/2015/0298), and conducted following the principles of the Declaration of Helsinki and the guidelines for Good Clinical Practice.

Mouse studies were carried out with the approval of the IDIBELL Animal Ethics Committee (Procedure 18012/10482 AFF), in compliance with the AAALAC for the Care and Use of Laboratory Animals.

## 3 Results

### 3.1 *PRRX1* is expressed in human melanoma primary tumors and associated with EMT

**Fig. 1:**
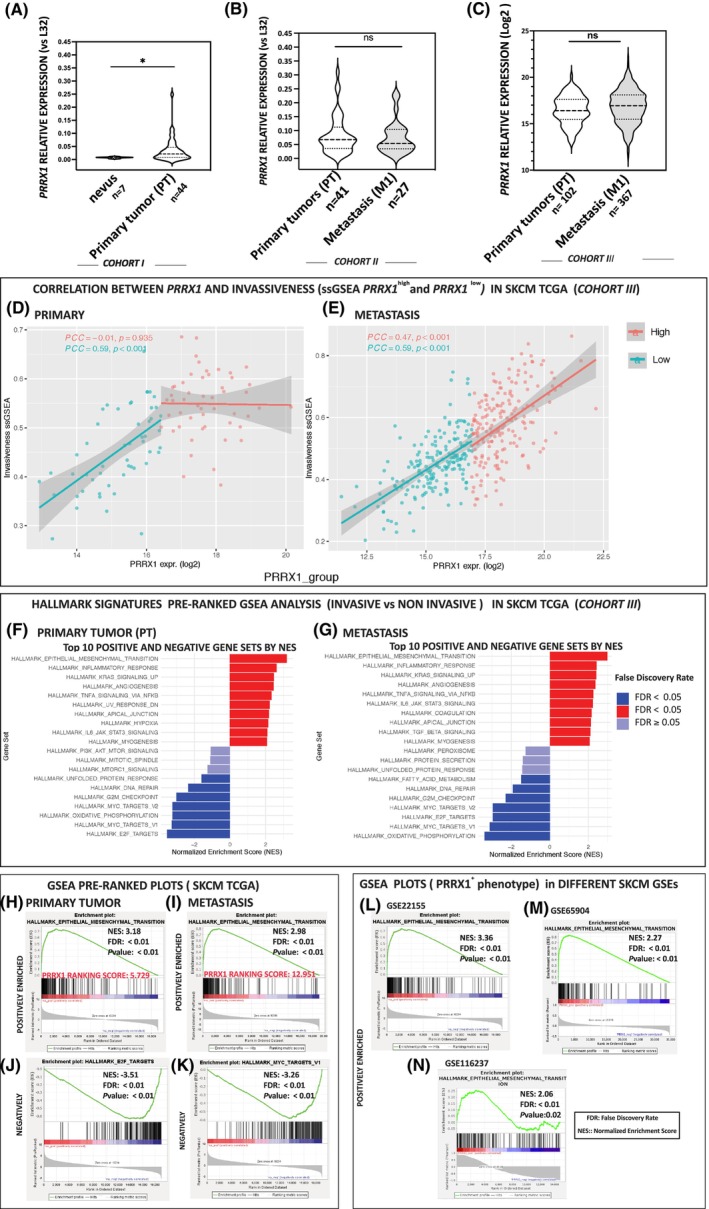
Expression of *PRRX1* mRNA in human melanoma samples. Violin plots depicting the expression of *PRRX1* mRNA levels in (A) benign *nevi* and primary melanoma samples (SSM cohort I); (B) in primary and metastases from advanced melanoma samples (Cohort II); (C) in primary tumors and metastases from the SKCM‐TCGA dataset (Cohort III). *P*‐values were calculated by the Mann–Whitney test: *P* = 0.036 is indicated by an asterisk * in panel A, and non‐significant (ns) in panels B and C; (D, E) Correlation between *PRRX1* and invasiveness by Single Sample Gene Set Enrichment Analysis (ssGSEA) (high/low *PRRX1* expression) in primary tumors (D) and metastasis (E) from the SKCM‐TCGA dataset; (F, G) Bar charts showing the most significantly enriched Gene sets by NES in primary tumors (F) and metastasis (G) obtained by pre‐ranked GSEA (invasiveness vs non‐invasiveness) in SKCM‐TCGA dataset. Bars indicate the up‐pathways (positively correlated, in red) and down‐pathways (negatively correlated, in blue), according to FDR. The Epithelial_Mesenchymal_Transition (EMT) signature was the first in the Top 10 positive Gene sets; (H, I) Showing the Pre‐Ranked GSEA plots of “EMT” gene signatures exhibiting similar significant enrichment in primary tumors and metastasis respectively of the SKCM‐TCGA dataset. The presence of *PRRX1* is indicated (*PRRX1* ranking score); (J, K) Representative plots of E2F and Myc Target hallmarks negatively enriched in pre‐ranked GSEA; (L–N) GSEAs showing positive enrichment of epithelial to mesenchymal transition (EMT) signatures in the GSE22155, GSE65904, and GSE116237 datasets. A *t*‐test was used to compare two situations, and significance was considered as *P* < 0.05. Genes were ranked based on their Pearson correlation with *PRRX1* (panels L–N). The NES (Normalized Enrichment Score), FDR (False‐Discovery Rate), and *P* are indicated in all GSEA plots.

*PRRX1* expression was detected in benign *nevi* and primary melanomas in two independent sets of our melanoma patients (cohorts I and II), finding that levels were significantly increased in non‐invasive melanoma (< 1.79 mm^3^) (cohort I) compared with *nevi* (Fig. 1A) (all clinical and histological characteristics of melanoma samples are described in Table S1). Moreover, increased *PRRX1* levels were detected in advanced primary tumors from cohort II compared with non‐invasive melanomas. However, *PRRX1* levels in metastasis samples were lower than in primaries, albeit without statistical significance (Fig. 1B). Of note, samples from primary tumors do not match metastasis samples. Computing the Pearson's correlation coefficient between *PRRX1* expression and clinicopathological features of melanoma patients, we found that lymphatic invasion (*R* = 0.277, *P* = 0.05) was significantly associated with high levels of *PRRX1* in primary tumors (cohort II). Other parameters, such as TNM stage, AJCC stage, age, and sex, were not associated with *PRRX1* expression in cohort III (Table S2).

Given the key role of PRRX1 in promoting the invasive phenotype in carcinomas and other non‐epithelial tumors, our initial purpose was to analyze whether its expression in melanoma also relies on invasion. To this end, we defined an “invasiveness set of genes” to compare the gene expression data (High/Low expression of *PRRX1*) provided by the SKCM‐TCGA dataset. To build the “invasiveness set”, we compiled evidence from the literature preceding the invasive phenotype in melanoma studies and genes defined as Hallmark EMT as described in Section 2.

We carried out non‐parametric ssGSEA to compute signature values in individual cell transcriptomes (*n* = 107 primary tumor samples, and *n* = 367 metastasis). The invasiveness score is shown in Table S7. As shown in Fig. 1D,E, *PRRX1* expression correlates with invasiveness in both primary and metastatic samples of the SKCM‐TCGA dataset, albeit with remarkable differences in primary tumors, with high PRRX1 expression levels. In these samples and based on Pearson correlation (PCC = −0.01, *P* < 0.05), an increase in PRRX1 levels is not accompanied by an increase in invasion, which could already be sufficient to invade surrounding territories.

Since the activities of genes are precisely coordinated to execute cellular functions, we carried out GSEA analysis to unveil signaling and cellular pathways associated with invasiveness versus non‐invasiveness. GSEA was performed on the pre‐ranked genes (invasiveness vs non‐invasiveness) of the SKCM‐TCGA dataset. According to the FDR value, this yielded 50 Hallmark gene sets significantly enriched in either primary tumor or metastatic samples with high *PRRX1* expression. Figure 1F,G show the top 10 of these, and as expected, the EMT was significantly enriched at the top followed by “Inflammatory response” (commonly associated with EMT); “KRAS_Signaling‐UP”; “Angiogenesis”, and “TNF_Signaling via NFKB”, among others (see Table S8). In contrast, “E2F” and “MYC_Targets” were significantly enriched in low‐expression *PRRX1* samples, denoting activation of the E2F and MYC gene sets, which might favour cell proliferation and tumor growth rather than invasion. Figure 1H,I show the “EMT” positively enriched plots from pre‐ranked GSEA (invasive vs non invasive) in primary tumors and metastasis, respectively. Figure 1J,K show the negatively enriched plots “E2F” and “MYC Targets”.

To complement the study, we explored pathways associated with *PRRX1* expression in independent GEO datasets GSE65904, GSE22155, GSE50509, and GSE116237 by GSEA. In this case, we simply ranked genes based on their Pearson distance to *PRRX1*. In agreement with the above results, the EMT hallmark was significantly enriched at the top of this co‐expression ranking (False Discovery Rate < 0.01), as shown in Fig. 1L,M (see the ranking of genes in Table S9).

Altogether, these results allow us to validate the expression of members of the EMT gene set, especially upon PRRX1 depletion (see Sections 3.3, 3.4 and 3.6). They may also open new studies on other gene sets expressed in melanoma lesions that could provide further insight into disease biology.

### 3.2 *PRRX1* expression in metastatic samples is a useful biomarker for predicting the early mortality of melanoma patients

**Fig. 2:**
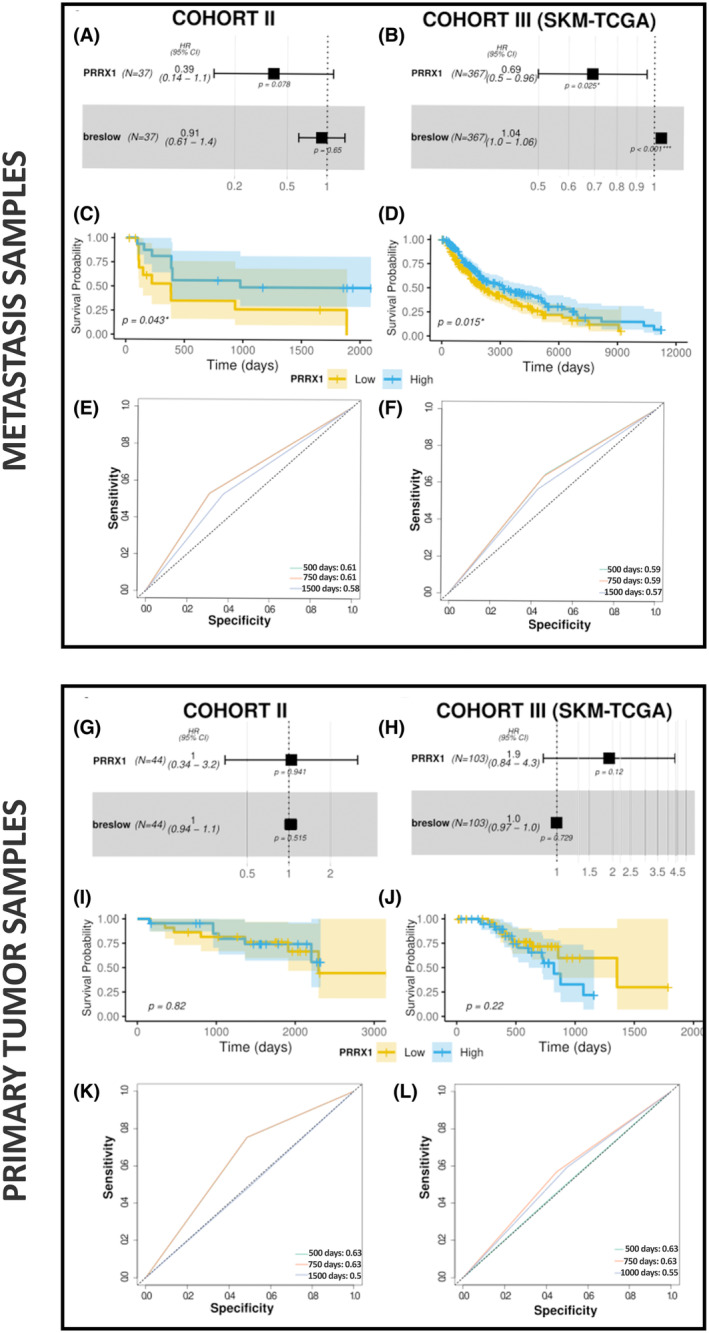
Assessment of *PRRX1* for Prognostic Prediction and survival analysis of cutaneous melanoma patients. The analysis was performed in metastasis (upper panels A–F) and primary tumor samples (bottom panels G–L) and adjusted for the Breslow score. The inner left panels show analysis results for Cohort II and the inner right panels for Cohort III (SKCM‐TCGA). Inner panels: (A, B) and (G, H): Forest plots for the multivariate Cox regression models. HR together with 95% CIs are shown. Below each square are the *P*‐values for each variable. (C, D) and (I, J): Kaplan–Meier analysis comparing *PRRX1* expression at high (blue) and low (yellow) levels. Shaded areas around the curve indicate 95% CI. (E, F) and (K, L): ROC curves for the time‐dependent analysis for the indicated days. AUC, area under the curve; CI, confidence interval; HR, hazard ratio; *N*, sample size; *P*, *P*‐value.

To evaluate the clinical significance of *PRRX1* expression in melanoma, *PRRX1* levels were analyzed by qPCR in primary tumors and metastasis samples from our patients. Gene expression levels of *PRRX1* were categorized as high or low, based on the median expression value. A multivariate Cox regression model was performed to evaluate the association between *PRRX1* expression and survival, adjusted for the Breslow score. Furthermore, our analysis revealed that there is no statistically significant linear relationship between PRRX1 levels and Breslow score (*P* ≥ 0.05) (Fig. S1A,B). Analysis of metastasis samples suggested that low *PRRX1* expression was an independent prognostic predictor of reduced overall survival (OS) in cohort II ([HR]: 0.39, 95% [CI]: 0.14–1.1, *P* = 0.078; number of patients [*N*] = 37) (Fig. 2A). These results were confirmed using SKCM‐TCGA cohort III (HR: 0.69, CI: 0.5–0.96, *P* = 0.025; *N* = 367) (Fig. 2B). Additionally, Kaplan–Meyer curves for metastasis samples showed significant differences between low and high values of *PRRX1* (*P*_cohort II_ = 0.043; *P*_cohort III_ = 0.015) (Fig. 2C,D) but not in primary tumor samples (*P*_cohort II_ = 0.82; *P*_cohort III_ = 0.22; [*N*] = 104) (Fig. 2I,J). Time‐dependent analysis for 500, 750, and 1500 days in metastasis samples yielded AUCs of 0.61 [0.52–0.70], 0.61 [0.52–0.70], and 0.58 [47, 48, 49, 50, 51, 52, 53, 54, 55, 56, 57, 58, 59, 60, 61, 62, 63, 64, 65, 66, 67, 68, 69] respectively, for cohort II (Fig. 2C). Similar outcomes were found in cohort III for 500, 750, and 1500 days with AUCs of 0.59 [0.55–0.63], 0.59 [0.55–0.63], and 0.57 [0.54–0.60], respectively (Fig. 2F). The same analysis in primary tumor samples did not show any significant results in cohort II (HR: 1, CI: 0.34–3.2, *P* = 0.941; [*N*] = 44) (Fig. 2G) or cohort III (HR: 1.9, CI: 0.84–4.3, *P* = 0.12; [*N*]: 103) (Fig. 2H). Results suggest that *PRRX1* in metastasis samples is a useful biomarker for predicting the early mortality of patients. Moreover, adjusting for the Clark score did not alter the results for *PRRX1* (Fig. S2).

### 3.3 *PRRX1* expression in human melanoma correlates with the *BRAF* ^*V600E*^ mutation

**Fig. 3:**
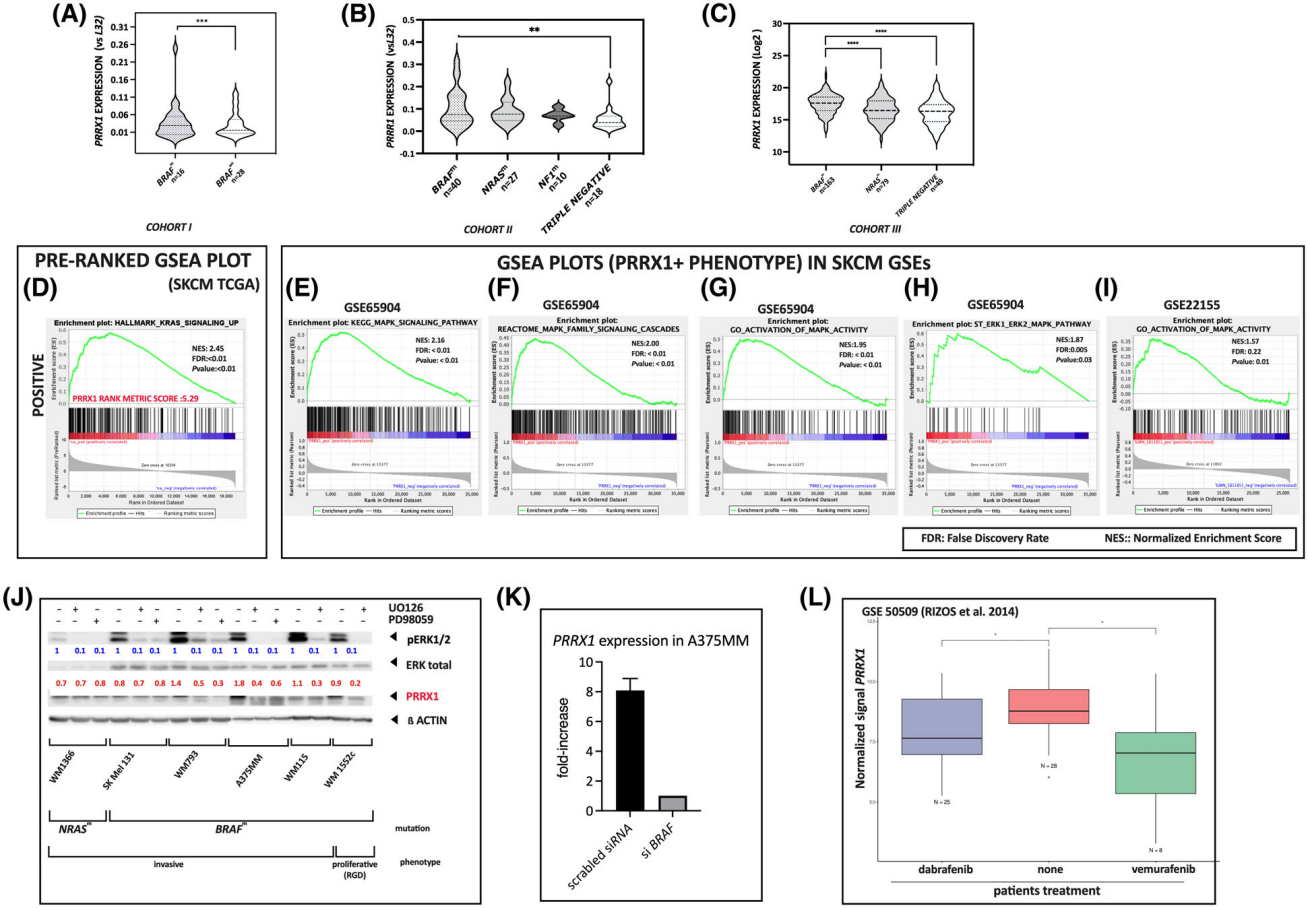
*PRRX1* expression in human melanoma correlates with the *BRAF* ^*V600E*^ mutation (A–C) *PRRX1* expression according to *BRAF* ^*V600E*^/*NRAS* ^*Q61L*^ prevalent mutations; (A) Cohort I; (B) Cohort II; (C) Cohort III from the SKCM‐TCGA dataset, including primary tumors and metastases. The central dashed lines depict the median and dot lines reflect the lower and upper quartiles. *P*‐values were calculated by the Mann–Whitney test (*P < 0.01, **P < 0.001, ***P < 0.0001). (D) Plot from pre‐ranked GSEAs (KRAS Signaling in the SKCM‐TCGA dataset) showing enrichment of gene sets related to MAPK activation; (E–I) GSEA plots associated with MAPK activation from different GSE datasets. For E–I plots we ranked genes based on their Pearson correlation with *PRRX1*. FDR, false‐discovery rate; NES, normalized enrichment score. (J) Detection of PRRX1 and ERK proteins in several melanoma cell lines treated with MEK inhibitor (UO126), ERK inhibitor (PD98059), or vehicle. α Tubulin was used as a loading control. The relative intensity of pERK signals was calculated by the respective total ERK and is shown in blue. PRRX1 quantification of signals was calculated by the respective α Tubulin and is shown in red. This is a representative immunoblot from three independent experiments. The mutational status and the phenotype of the cell lines are provided; (K) Box plot of *PRRX1* expression in short‐term A375MM cells transfected with *PRRX1*siRNA or non‐targeting control (scrambled). Mean and error bar (SD) were calculated by Student's *t*‐distribution from three independent experiments with technical duplicates; (L) Box plot showing the distribution of normalized *PRRX1* gene expression across three treatment groups: None (untreated), Dabrafenib, and Vemurafenib for the GSE50509 dataset [45]. Significant differences between groups were calculated by Student's *t*‐distribution (*P*‐value < 0.05 is represented with *). The number of samples (*N*) per group is provided below each boxplot.

We explored the presence of mutation *BRAF* ^*V600E*^ and found that 63.6% (28/44) of tumors in cohort I presented this mutation. Relative *PRRX1* expression was higher in those *BRAF* ^*V600E*^ melanomas compared with *wild‐type BRAF* (Fig. 3A). We then assessed whether *PRRX1* expression differed according to *NRAS*/*BRAF/NF1* mutation status in an additional set that included primary and metastasis samples (cohort II). *BRAF* ^*V600E*^ and *NRAS* ^*Q61K*^ mutations were detected in 42.1% (40/95) and 28.42% (27/95) of samples, respectively, and *NF1* mutations in 10.5% (10/95) of samples. Relative *PRRX1* expression was significantly higher in *BRAF*‐mutated samples compared with triple‐negative samples (Fig. 3B). To confirm these findings, *PRRX1* expression was evaluated in SKCM‐TCGA samples containing the mutational status (cohort III) finding similar results (Fig. 3C). Furthermore, we classified these samples according the *PRRX1* expression using the median as the threshold and performed pre‐ranked GSA comparing *PRRX1* ^high^ versus *PRRX1* ^low^group (Table S8). In this case, the “KRAS_Signaling‐Up” hallmark was found in the second position of the 10 top significantly enriched pathway in high *PRRX1* samples, preceded by the EMT (Fig. S3). Figure 3D shows the enrichment plot of *PRRX1* and the “KRAS_Signaling‐Up” hallmark obtained by pre‐ranked GSEA in SKCM‐TCGA samples. Accordingly, *PRRX1* expression was associated with the *BRAF* ^*V600E*^ mutation. Given that BRAF^m^ proteins phosphorylate MEK1/2, subsequently activating ERK1/2 [2] we explored any potential relationship between this signaling pathway and *PRRX1* expression. We extended the studies to GSE22155, and GSE65904 datasets by GSEA, here ranking the genes based on their Pearson correlation with *PRRX1* (Fig. 3E–I and Table S10). In summary, MAPK gene sets contained genes positively correlated with *PRRX1*.

Furthermore, we analyzed PRRX1 expression levels in human melanoma cell lines and the effects of MEK/ERK inhibition *in vitro*. We found that inhibition by either UO126 or PD98059 inhibitors diminished PRRX1 protein levels in melanoma cell lines carrying the *BRAF* ^*V600E*^ mutation (Fig. 3J). Moreover, silencing *BRAF* in A375MM cells caused a substantial decrease in the *PRRX1* expression (Fig. 3K). In line with these results, we explored *PRRX1* expression in biopsies from *BRAF* ^*V600*^‐mutated metastatic melanoma patients analyzed for mRNA expression in the study of Rizos et al., 2014 in the GSE50509 dataset [45]. *PRRX1* expression levels were significantly reduced after patients were treated with either dabrafenib (*n* = 25) or vemurafenib (*n* = 8), compared with those that had not received BRAF inhibitors (*n* = 28) (Fig. 3L). Importantly, the authors reported that loss of MAPK activation occurs early in treatment‐responding melanoma tumors and in a subset of BRAF inhibitor‐resistant progressing metastases. Together, these results provide support at least in part for MAPK‐mediated PRRX1 expression in melanoma.

### 3.4 Targeted inhibition of PRRX1 increases cell proliferation and favours a switch towards a de‐differentiated melanoma cell state

**Fig. 4:**
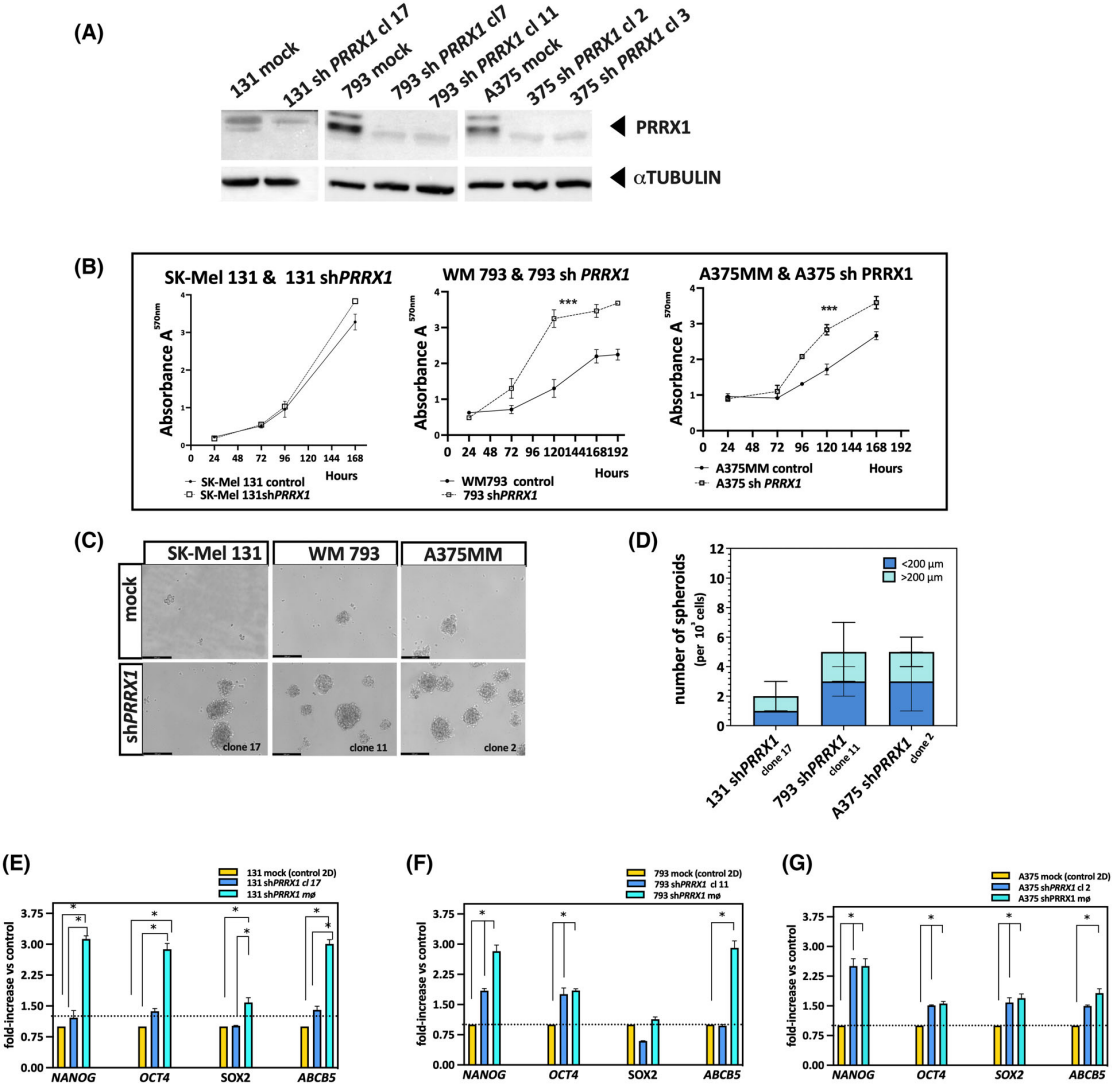
PRRX1 silencing in human melanoma cell lines increases the proliferation and expression of Cancer Stem Cell markers. (A) Detection of PRRX1 in SK‐Mel 131, WM 793, and A375MM control cells (infected with pLKO.1 empty vector, mock) or PRRX1‐silenced cells. One representative immunoblot of three independent experiments is shown. α Tubulin was used as a loading control. (B) Cell proliferation *in vitro* in the presence of 2.5% Fetal Calf Serum. Diagrams show the A^570 nm^ for mocks, and their respective PRRX1‐silenced clone at different times after platting the cells. The mean and SEM of technical quadruplicates are indicated, and significant differences between groups were calculated by Student's *t*‐distribution. * Statistical significance was considered *P* < 0.05. *P* < 0.01 in the middle and right panel is indicated by *** asterisks. One out of three experiments is presented. (C) One out of three independent experiments in 3D cultures showing phase‐contrast images of fourth‐generation spheroids in silenced cells. Upper panels: mock Mel 131; mock 793; and mock A375MM cells. Bottom panels: the indicated sh*PRRX1* clones. Scale bars, 200 μm. (D) Quantification of spheroids (mø) formed by sh*PRRX1*‐silenced cells. The number of spheroids (mø) per field (observed at 10× magnification) is shown in the bar plot indicating the mean and SEM of technical replicates in one out of three independent experiments. (E–G) Relative expression of pluripotency transcription factors *NANOG*, *OCT4*, *SOX2*, and the side‐population marker *ABCB5* in control cells (mock, cultured in 2D) and PRRX1‐silenced cells cultured in 2D or 3D (spheroids, mø): (E) SK‐Mel 131; (F) WM 793; (G) A375MM. Results are presented as fold‐increase relative to the expression of control cells (mock) growing in 2D. One out of three independent experiments is shown and includes the mean and SEM of technical triplicates. *P*‐values were calculated by a two‐sided unpaired Student's *t*‐test *P* < 0.05.

Since the decreased expression of *PRRX1* in melanoma patients correlates with poor prognosis, we explored the biological consequences of stably silencing *PRRX1* expression in human melanoma cell lines. According to the results shown in Fig. S4, we chose those cell lines with high PRRX1 expression levels (WM793 and A375MM) exhibiting different morphology (pseudoepithelial and spindle, respectively) but belonging to the “invasive phenotype”, as described previously [51]. When indicated, SK‐Mel 131 cells, albeit with low PRRX1 expression, were included in experiments for stable silencing of *PRRX1*. We used a sh*RNA* sequence for *PRRX1* cloned into a lentiviral vector previously described by [36]. Independent clones of puromycin‐resistant cells were generated in each case, and the PRRX1 protein was analyzed by immunoblot (Fig. 4A). The expression of the target was reduced significantly in PRRX1‐silenced clones of WM793 and A375MM cells compared with cells infected with pLKO.1‐empty vector (hereafter called “mock” or controls), which maintained a robust PRRX1 expression. Moreover, *PRRX1*‐silenced cells had enhanced cell proliferation compared with their mock cells (Fig. 4B). In SK‐Mel 131cells, whose PRRX1 levels were lower than in the other cell lines, the effect of sh*PRRX1* on cell proliferation was less notorious. Considering that EMT accounts for reduced cell proliferation [52], it is not surprising that the abrogation of EMT causes an increase in cell proliferation. Enhanced cell proliferation in impaired EMT may favour the outgrowth of metastasis. The activation of cell cycle progression and the involvement of Cyclins D1, D2, and p21 upon PRRX1 depletion remains elusive.

We next asked whether PRRX1 loss enables SK‐Mel 131, WM793, and A375MM cells to adopt stem cell features. Firstly, we explored the sphere‐forming capacity, a test for stemness competence. As shown in Fig. 4C,D, the selected PRRX1‐silenced clones form a greater number and larger‐sized spheres than their respective controls. These spheroids maintained the capacity to grow in these conditions for at least eight consecutive passages (not shown). Furthermore, we explored the expression levels of the pluripotency transcription factors *NANOG*, *OCT4*, and *SOX2* and the side‐population marker *ABCB5* in PRRX1‐loss cells after three cycles of spheroids enrichment. Notably, we detected increased expression of *NANOG* in all cell lines growing in 3D conditions, while *OCT4* and *SOX2* expression was variable and only increased in SK‐Mel 131 and A375MM (Fig. 4E–G). Remarkably, both NANOG and SOX2 are expressed by multipotent proliferative neural crest cells [53]. Thus, since melanoma cells have a neural crest origin, we interpreted that these TF may play a similar role in melanospheres, favouring the capacity of cells for self‐renewal and differentiation.

### 3.5 PRRX1 loss impairs the cell migration and invasion of melanoma cells

**Fig. 5:**
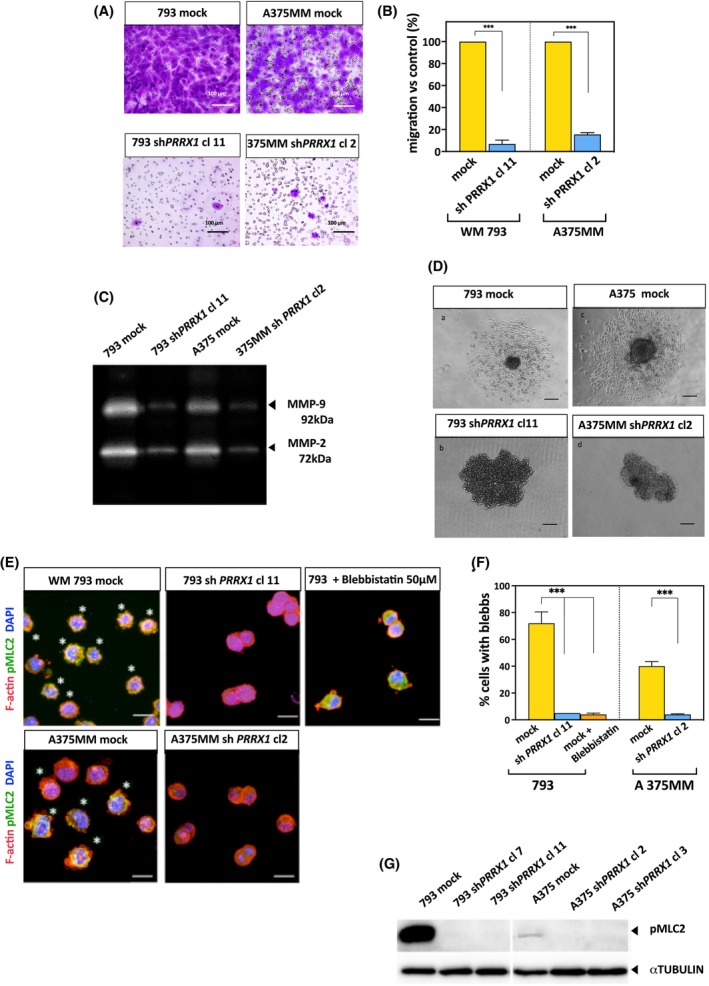
PRRX1 knockdown suppresses the migration and invasion of melanoma cells. (A) Cell migration of PRRX1‐silenced cells relative to control (mock) as detected by Transwell assay. Panels show the migrated cells on the downside of polycarbonate filters, stained with crystal violet. Scale bar, 100 μm. Images from one of three independent experiments are presented. (B) Migrating cells were quantified by measuring the A^570 nm^ of crystal violet stain eluted from membranes by 10% SDS. The bar plot shows the mean and SEM relative to controls of technical replicates. *P*‐values were calculated in the presented experiment by a two‐sided unpaired Student's *t*‐test and *P* < 0.01 is indicated by ***. (C) Gelatin zymography of conditioned media from controls (mock) and PRRX1‐silenced cells. Cleared bands show the position of secreted gelatinases on dark SDS‐gelatin gel stained with Coomassie blue. The zymograme is representative of two independent experiments. (D) Invasive growth was analyzed in controls and PRRX1‐silenced cells by embedding cells as spheroids in the bovine collagen I matrix. The images are representatives of two independent experiments. Inner panels: (a) 793 Mock (control); (b) 793 sh*PRRX1*; (c) A375 MM mock (control); (d) A375 sh*PRRX1* (Scale bar, 100 μm). (E) 3D collagen invasion assays for PRRX1‐silenced cells and controls. Representative confocal images of F‐actin (red), pMLC2 (green), and DAPI (blue). White asterisks indicate cells with blebs. Blebbistatin was used as a negative control. Scale bar, 20 μm. Images from one of four independent experiments are presented. (F) Quantification of cells with blebs. The numbers were obtained from at least 6 pictures per experiment. Bars show the mean and SD of three independent experiments. *P*‐values were calculated by a two‐sided unpaired Student's *t*‐test (***P < 0.001). (G) One of three representative immunoblots of pMLC2 in 793 mock, and A375MM mock cells and their respective clones upon knockdown of *PRRX1* is shown. α Tubulin was used as a loading control.

We investigated the impact of PRRX1‐loss on the migration and invasion of WM793 and A375MM cells. As shown in Fig. 5A,B, the PRRX1 knockdown dramatically reduced cell migration in Transwell assays as compared with controls. Furthermore, we analyzed the secreted gelatinases MMP‐9 and MMP‐2, which could contribute to collagen degradation and individual cell migration through the extracellular matrix and basal membranes. MMP‐9 and MMP‐2 activities were reduced in the conditioned media of PRRX1‐silenced cells compared with mock cells (Fig. 5C). These results indicated that pericellular proteolysis was ablated because of PRRX1 knockdown; thus, these cells could not fully achieve the canonical migratory/invasive mode of mesenchymal‐like cells. We further validated the invasive capacity of cells cultured as spheroids embedded in collagen I. As shown in Fig. 5D, invasive growth was dramatically impaired in PRRX1‐silenced cells compared with controls after 4 days of culture. Finally, we investigated if PRRX1 loss might alter the amoeboid‐blebbing behavior of melanoma cells, which use their actomyosin contractility to move and remodel the matrix when pericellular proteolysis is ablated [39]. By 3D imaging, we could observe blebb‐like structures in WM793 and A375MM mock cells but not in PRRX1‐knockdown cells or those treated with the inhibitor Blebbistatin. Importantly, cells were rounded upon PRRX1 loss (Fig. 5E,F). Accordingly, the levels of phosphorylated MLC2 were reduced in PRRX1‐silenced cells (Fig. 5G). These results indicate that PRRX1 could regulate actomyosin contractility and amoeboid features. Future investigations will address the mechanism(s) by which loss of PRRX1 causes these effects.

### 3.6 Downstream of PRRX1 loss, reduced expression of EMT‐transcription factors (TFs), and dysregulation of signaling pathways may account for impaired migration and invasion

We further examined the expression of other EMT‐TFs and signaling pathways that might be involved in the inhibition of cell migration and invasion in PRRX1‐deficient cells.

**Fig. 6:**
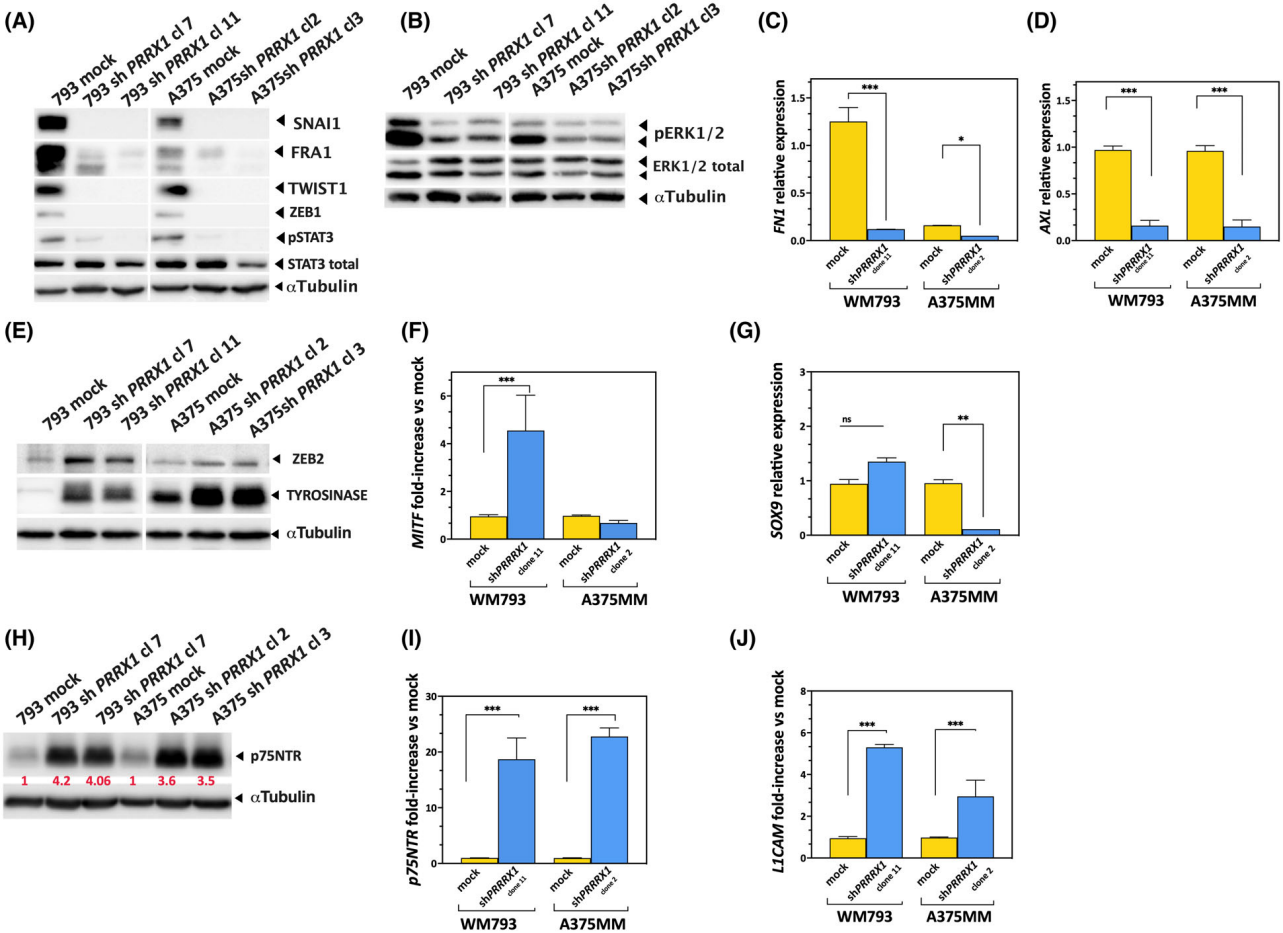
PRRX1 depletion leads to decreased expression of epithelial to mesenchymal transcription factors (EMT‐TFs) and signaling proteins. (A) Representative immunoblots showing expression of SNAI1, FRA1, TWIST1, ZEB1, and STAT3 Transcription Factors after stable knockdown of *PRRX1* in the 793 and A375MM cell lines. A significant decrease in SNAI1, FRA1, TWIST1, ZEB1, and pSTAT3 signals was detected in two independent *PRRX1*‐silenced clones compared with their respective controls (mock). The blot shown is one out of three independent experiments. α Tubulin signal was used as loading control; (B) Representative immunoblots for phosphorylated and total ERK upon stable knockdown of *PRRX1* in the indicated cell lines and their respective *PRRX1*‐silenced clones. α Tubulin was used as a loading control; (C, D) The bars in the bar plots show the expression of the mesenchymal marker *FN1* and *AXL* respectively, in control and *PRRX1*‐silenced cells. mRNA expression was measured by quantitative PCR and normalized by RP*L32*. Here are the respective mean and SEM of technical triplicates. *P*‐values were calculated by a two‐sided unpaired Student's *t*‐test (***P < 0.001, *P < 0.05). Shown one out of three independent experiments; (E) Shown here one out of three independent immunoblots for the ZEB2 and the melanocyte differentiation marker tyrosinase upon stable knockdown of *PRRX1* in the indicated cell lines and their respective controls. Compared with controls, a significant increase in tyrosinase signal was detected in PRRX1‐silenced clones. α Tubulin was used as a loading control. (F, G) The bars in the bar plot show the mean and error (SD) of the melanocytic marker *MITF* and *SOX9* expression respectively, in one out of two independent experiments. *P*‐values were calculated by a two‐sided unpaired Student's *t*‐test (***P < 0.001, **P < 0.01, ns indicates non‐signficant); (H) One out of two immunoblots from independent experiments is presented showing the increase of neurotrophin receptor (p75NTR) in sh*PRRX*1 cell lines compared with mock controls. Shown in red are the relative expression levels calculated by densitometry of signals relatives to α Tubulin; Panels (I, J) show the expression of the neural crest‐like markers *p75NTR* (NGFR) and *L1CAM* respectively, in mock controls and *PRRX1*‐silenced cells. mRNA expression was measured by quantitative PCR and normalized by RP*L32*. One of three independent experiments is plotted here, and the boxplot indicates the mean and SEM of technical triplicates. *P*‐values were calculated by two‐sided unpaired Student's *t*‐test ***P < 0.0001.

Remarkably, *PRRX1* knockdown was associated with reduced expression of the EMT‐activator TWIST1 (Fig. S5). In contrast, PRRX1 expression was maintained upon depletion of TWIST1 by a specific short hairpin *RNA* (sh*TWIST1*) (Fig. S5). Furthermore, in line with previous data [12], we found that *PRRX1* depletion in melanoma cells was associated with a reduced expression of BRAF‐inducible FRA1, the EMT‐activators ZEB1, and SNAI1, and tentatively, the phosphorylated form of STAT3, although the expression of total STAT3 was maintained (Fig. 6A).

Moreover, silencing *PRRX1* reduced phosphorylated ERK levels compared with controls (Fig. 6B), which might contribute to reduced cell migration [54]. Accordingly, the expression of the mesenchymal marker FN1 was found to be significantly reduced (Fig. 6C). Then, we explored the expression of the receptor tyrosine kinase *AXL* gene, which is involved in the migration and invasion of melanoma cells [55]. We detected *AXL* present in mock cells, however its expression was dramatically reduced in *PRRX1‐*depleted clones in both cell lines by qRT‐PCR (Fig. 6D). By contrast, the expression of ZEB2 was increased in *PRRX1*‐silenced cells (Fig. 6E).

These results suggested that depletion of PRRX1 in invasive melanoma cells leads to a phenotype switch owing to their transcriptional reprogramming into a proliferative and more differentiated phenotype. To this end, we first explored *MITF* expression levels in mock and PRRX1‐depleted cells since the expression of this well‐known melanoma differentiation marker has been used to define proliferative versus invasive phenotype [12, 15]. As shown in Fig. 6F, *MITF* expression was significantly increased in 793 *PRRX1*‐silenced cells, compared with their controls. Accordingly, an increase in tyrosinase expression was observed in all *PRRX1*‐loss cells (Fig. 6E). Furthermore, we did not find any significant changes in levels of SOX10 expression (data not shown), whereas those of SOX9 were significantly decreased in A375MM sh*PRRX1* cells (Fig. 6G).

To further investigate the co‐expression or antagonistic expression of other markers of melanoma cell states and *PRRX1*‐loss, we explored the expression of the melanoma stem cell marker CD271 (also named p75NTR or NGFR) [21, 23]. We witnessed that p75NTR expression was significantly increased at the protein and mRNA levels in *PRRX1*‐silenced clones compared with controls that exhibited scant expression (Fig. 6H,I). Of note, cells were cultured in regular media instead of a medium that may promote neural differentiation. This finding raises the question of whether p75NTR is involved in the melanoma phenotype switching due to the loss of *PRRX1* expression.

We then analyzed the expression of L1CAM, also a known NCSC marker [44]. As shown in Fig. 6J, its expression was significantly increased in both cell lines after impairing the PRRX1 expression. This needs to be addressed further in future experiments, nevertheless, the results suggest that *PRRX1*‐loss may facilitate the up‐regulation of proliferative/melanocytic genes specific to the melanocytic lineage, counteracting the EMT phenotype while promoting a neural crest‐like phenotype.

### 3.7 PRRX1 depletion increases lung colonization

**Fig. 7:**
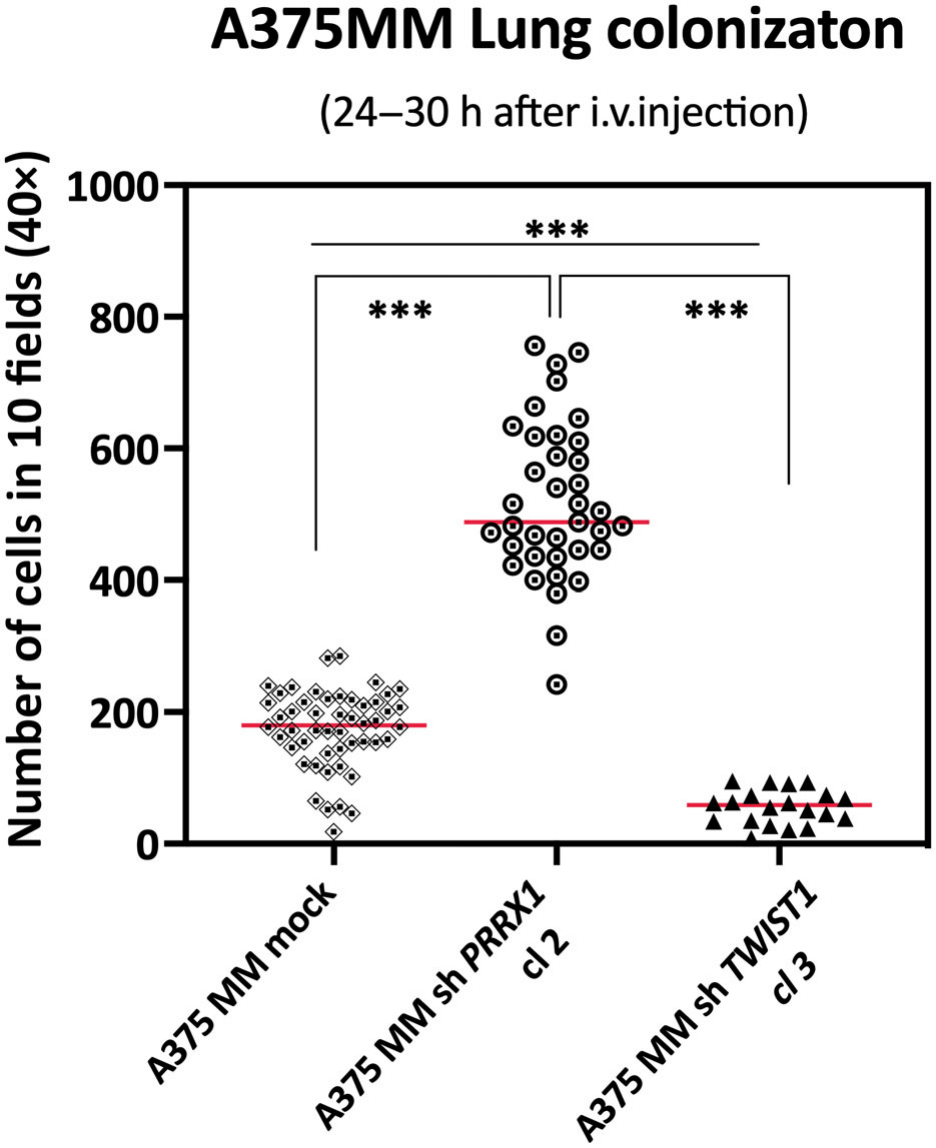
Silencing PRRX1 in melanoma cells increases lung colonization in NSG mice. Quantification of A375MM EGFP‐labeled tumor cells retained into the lung parenchyma 24 h after intravenous injection of cells. Dots represent the number of tumor cells detected in 10 fields (20×) of lung sections from three independent experiments. NSG mice were injected with A375MM control (mock) cells (*n* = 16); A375MM sh*PRRX1* cells (*n* = 10) or A375 sh*TWIST1*cells (*n* = 5). Individual values are plotted, and the red horizontal lines depict the respective median. Significance was determined using the unpaired Student's *t*‐test. *P* < 0.001 is indicated by ***. See Fig. S6 for representative images.

An important aspect of cancer progression is the ability of cells to colonize distant territories and develop metastasis. We exemplified the colonization capacity of PRRX1‐silenced cells by i.v. injection of A375MM EGFP‐labeled cells into NSG mice and quantifying the number of tumor cells retained in the lung parenchyma 24 h later. We found that PRRX1 loss led to a significant increase in single EGFP‐positive cells in the lungs as compared with mock cells, indicating that PRRX1 depletion favours lung colonization (Fig. 7). By contrast, the depletion of TWIST1 decreased the number of EGFP‐positive cells retained in the lung parenchyma (Fig. 7, Fig. S6A–C). Remarkably, similar numbers of green‐fluorescent tumor cells were detected in the lungs 1 h after i.v. injection of controls and either PRRX1‐ or TWIST1‐knockdown cells, indicating that cells equally reached the lungs (Fig. S6A′–C′).

### 3.8 PRRX1‐loss in melanoma cells enhances the primary tumor cell proliferation and spontaneous metastasis in xenografts

We analyzed the tumorigenicity and the capacity to spontaneously develop metastasis in distant organs of NSG mice after orthotopic injection of SK‐Mel 131‐, WM793‐ or A375MM‐mock and their corresponding *PRRX1*‐silenced cells. Primary tumors (< 3 mm^3^) were macroscopically detected at day 10 ± 2 after injection in mice. The growth ratio (mm^3^·day^−1^) of xenografted tumors was calculated in the exponential growth phase. For 131‐mock, the ratio was 7.64 ± 2.7 and 7.6 ± 2 in 131sh*PRRX1* xenografts. The ratio for 793‐mock was 8.8 ± 1 and 7.51 ± 3 in 793‐sh*PRRX1*, and in the A375MM model, it was 13.53 ± 4 for controls and 14.16 ± 2 in the case of A375MM‐sh*PRRX1*. Accordingly, in NSG mice, no significant differences in tumor volume were found between control and PRRX1‐silenced cells. We surgically excised the primary tumors (< 100 mm^3^) and mice were kept alive until sacrificed. Visceral and lymph node metastases were detected macroscopically at necropsy and the results are summarized in Table 1. The incidence of metastases was defined as the percentage of mice harboring one or more metastases in a particular organ. Importantly, the incidence of spontaneous metastases in the lungs and liver was significantly increased in PRRX1‐silenced xenografts compared with their respective controls. The number of metastases in the lungs and liver detected in each mouse was also significantly increased in 793‐ and 375MM‐sh*PRRX1* compared with their respective controls, yet not the size of the lesions (Table 2). In mice bearing *PRRX1*‐silenced cells, small and large metastases were distributed in the sinusoid and liver parenchyma, replacing the normal liver.

**Table 1:** Incidence of spontaneous metastasis in orthotopic xenograft mice assay. Orthotopic models were performed by intradermal injection of 2 × 10^6^ cells (100 μL) cells into the right flank of 8‐week‐old NSG mice. Primary tumors were excised when reaching a volume of 100 mm^3^, and mice were kept alive until sacrifice. At necropsy metastatic lesions were visualized and scored by gross examination under a Leica ST microscope. The incidence of metastases was defined as the percentage of mice harboring one or more metastases in a particular organ and was calculated for all mice included in each group. Day of sacrifice is indicated by mean and SD (±) for all mice in each group.

Incidence of spontaneous metastasis in NSG mice					
	Lung	Liver	Lymph nodes	Sacrifice (day)	Number (mice)
SK‐Mel 131 mock	3/11 (27.27%)	4/11 (36.36%)	10/11 (90.9%)	62 ± 18	11
Mel 131 sh *PRRX1*	5/5 (100%)	1/5 (20%)	1/5 (20%)	60 ± 0	5
WM 793 mock	1/20 (5%)	1/5 (20%)	0/20 (0%)	60 ± 14	20
793 sh *PRRX1*	10/10 (100%)	10/10 (100%)	2/10 (20%)	60 ± 2	10
A375 MM mock	4/9 (44.4%)	3/9 (33.3%)	1/9 (11.1%)	95 ± 10	9
375 MM sh *PRRX1*	10/10 (100%)	10/10 (100%)	9/10 (90%)	60 ± 2	10

**Table 2:** Number and size of spontaneous metastasis in orthotopic xenograft mice assay. Metastatic lesions from orthotopic tumors were visualized at necropsy and scored by gross examination under a Leica ST microscope. The number of metastases in the lungs and liver per individual mouse was recorded, and the major diameter of the lesions was measured. Means ± SD are shown calculated for all mice included in each group. The number of mice and day of sacrifice are indicated in Table 1.

Number and size of spontaneous metastasis in NSG mice				
	Lung		Liver	
	Number of metastases /mice	Ø (mm) of metastases	Number of metastases/mice	Ø (mm) of metastases
SK‐Mel 131 mock	5.5 ± 0.5	1.2 ± 0	2 ± 0	2.35 ± 0.39
Mel 131 sh *PRRX1*	6.5 ± 1.323	1.94 ± 0.48	1 ± 0	1.43 ± 0.03
WM 793 mock	2.75 ± 0.75	0.84 ± 0.07	4 ± 0.75	3.3 ± 0.9
793 sh *PRRX1*	19.3 ± 2.1	1.4 ± 0.1	8 ± 3.7	2.2 ± 0.44
A375 MM mock	10.5 ± 0.5	3 ± 0	13 ± 1.5	4.65 ± 0.65
375 MM sh *PRRX1*	22.2 ± 5.6	1.8 ± 0.39	17.2 ± 7.4	4.24 ± 0.44

**Fig. 8:**
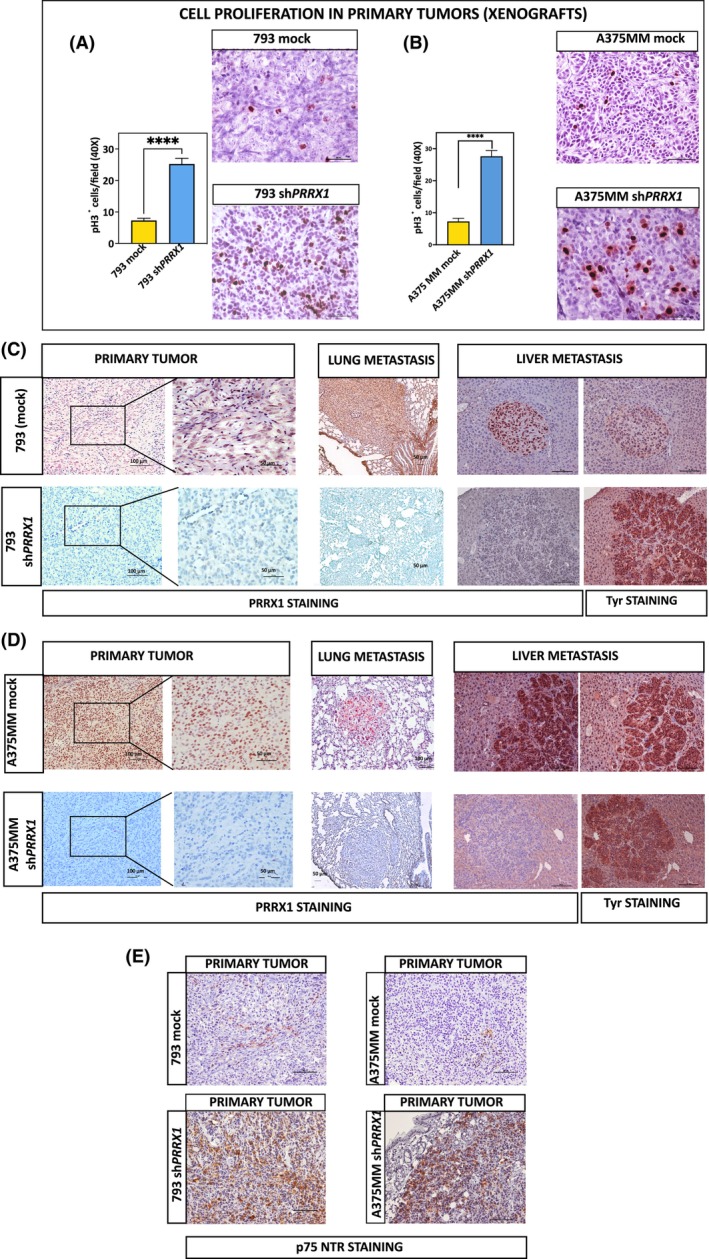
Silencing PRRX1 in melanoma cells increases cell proliferation in primary tumors and spontaneous metastasis in NSG orthotopic xenografts. (A, B) Detection of cells undergoing mitosis by phospho‐ Histone 3 (pH3) immunostaining in 793's (A) and A375MM (B) primary tumors. Left bar plots show the mean and SEM of pH3 positive cells in either controls or sh*PRRX*, detected in xenografts (40×) (6 fields per tumor and *n* = 5 independent tumors per group). ****P < 0.0001 determined by unpaired *t*‐tests. Bars, 50 μm. (C, D) Silencing PRRX1 in melanoma cells increases spontaneous metastasis in NSG mice. Immunohistochemical images of PRRX1 expression in primary tumors and spontaneous liver and lung metastasis from human melanoma xenografts. Representative images of the primary tumor, liver, and lung sections from NSG mice after orthotopic injection of 793 mock or 793 sh*PRRX1* (C) cells and A375MM mock or A375sh*PRRX1* (D) cells, respectively. One out of three independent experiments are presented. The positive immunostaining for PRRX1 or Tyrosinase is indicated at the bottom. Both stainings were made from consecutive sections of the same sample. PRRX1 positive staining was visualized as an intense reddish‐brown stain in the nuclei of cells of the primary tumor and metastatic foci of mock controls (upper panels). Positive immunoreactivity was also detected in collagen fibers, fibroblasts, and the dermis surrounding the primary tumor of both groups. Remarkably, the nuclei of the primary tumors or metastatic foci from sh*PRRX1* were negative for PRRX1 immunostaining although a diffuse stain was observed in the cytoplasm. Scale bars 100 μm (primary tumors and liver metastasis) and 50 μm (enlarged areas of primaries and lung metastasis, except when indicated). All sections were counterstained with Hematoxylin. (E) Detection of p75 NTR in primary tumors of melanoma xenografts. One out of three independent experiments are presented. Representative images of primary tumor sections from NSG mice after orthotopic injection of 793 mock or 793 sh*PRRX1* cells and A375MM mock or A375 sh*PRRX1* cells. Immunostaining for p75NTR is indicated at the bottom and was visualized as an intense reddish‐brown stain in cell membranes and cytoplasm of the sh*PRRX1* primary tumors (bottom panels). In contrast, positive staining was only seen in isolated few cells of mock controls (upper panels). Scale bars 100 μm. All sections were counterstained with Hematoxylin.

Histological characterization of primary tumors revealed qualitative differences between control and *PRRX1*‐silenced cells. Remarkably, we observed a dramatic increase in melanin production in primary tumors derived from *PRRX1*‐silenced cells, contrasting with the achromic aspect in controls corroborating the previous macroscopic visualization of samples (Fig. S7). Moreover, the cell density in primary tumors from *PRRX1*‐silenced cells was significantly increased compared with controls, suggesting a nodular growth pattern. The number of cells undergoing mitosis was increased in *PRRX1*‐silenced tumors compared with controls, as assessed by pH3‐immunopositive cells (Fig. 8A,B).

The expression of PRRX1 was evaluated by immunohistochemistry in histological samples of WM793 and A375MM primary tumors, lungs, and livers of mice. Positive immunostaining for PRRX1 was visualized as an intense reddish‐brown stain in the nuclei of cells at the primary tumor and metastatic foci of mock controls (upper panels Fig. 7D,E). Interestingly, only one mouse inoculated with 793 control cells presented lung and hepatic metastases, which stained positively for the PRRX1 antibody. Similar patterns were observed in lung and liver lesions from A375MM controls. Positive immunoreactivity was also detected in collagen fibers and the dermis surrounding the primary tumor of the WM793 and A375MM systems. Remarkably, the nuclei of neither primary tumors nor metastatic foci from sh*PRRX1* cells presented PRRX1 immunopositivity, although a diffuse stain was observed in the cytoplasm (bottom panels Fig. 8C,D). Interestingly, we observed the presence of melanin in primary tumors and metastasis as well as positive staining for tyrosinase in PRRX1‐negative metastatic foci in the liver of both xenografts (right panels Fig. 8C,D).

Moreover, we witness a significant increase in p75NTR‐immunopositive cells in primary tumors belonging to PRRX1‐depleted cells compared with the scant presence in mock controls in which only p75NTR‐positive cells were detected in very limited regions of tumors (Fig. 7E).

Collectively, these data indicate that depletion of PRRX1 does not prevent the primary tumor growth, but rather leads to an increase in cell proliferation and p75NTR expression, and significantly favours the development of spontaneous visceral metastases in melanoma xenografts. Overall, our results highlighted the importance of PRRX1‐loss *in vivo* mediating melanoma progression.

## 4 Discussion

Based on our findings, PRRX1 exerts two distinct roles during metastatic melanoma progression that appear to be highly dependent on its expression levels. This study shows that *PRRX1* expression is detected in benign nevi and, according to a recent report, its levels increase in primary tumors [56]. We show that *PRRX1* expression is dependent on MAPK activation and correlates with “invasiveness” since it forms part of the top 50 genes enriched in the EMT signature. Conversely, our study also reveals that low *PRRX1* expression in metastatic samples is an independent prognostic predictor of reduced OS in melanoma patients. Remarkably, the same analysis for primary tumor samples did not show any significant results in patient survival.

Our data suggest that, while PRRX1‐mediated EMT is required at the primary tumor, metastatic melanoma cells may impair this overexpression to succeed in metastasis, likely occurring in breast and HCC [26, 34, 35, 36].

Similar controversies regarding the contribution of EMT to tumor progression (having correlated EMT status with the ability of cells to escape the primary tumor), and the requirement of the reversal process, termed MET, for the establishment of macrometastases have been extensively discussed in carcinomas [4, 9, 36, 57, 58]; however, much less is known in melanomas.

Reprogramming the expression of different EMT‐TFs, including ZEB1/2, TWIST, SNAI2, and SNAI, in either melanoma cells or their microenvironment, has been associated with melanoma progression [12, 56, 59, 60]. However, whether PRRX1 expression impacts metastatic overgrowth in melanoma has not been reported. In this study, we aimed to unveil the consequences of PRRX1 loss in melanoma cells and its contribution to metastasis. We show that silencing *PRRX1* in mesenchymal‐like melanoma cells is not compensated by the overexpression of other proinvasive EMT‐TFs, rather it causes the impaired expression of FRA1, SNAI1, TWIST1, ZEB1 counteracting mesenchymal features and EMT signatures. Importantly, these changes are not impeded by the presence of the *BRAF* ^*V600E*^ mutation in these cells. Moreover, the reduction of PRRX1 levels induces the expression of ZEB2, an increase in melanin production and cell proliferation *in vitro*. Accordingly, PRRX1 loss does not impede the growth of primary tumors, which have a different growth pattern with increased proliferation as compared with the achromic tumors generated by control cells, suggesting that depletion of PRRX1 triggers a phenotype switch back to a more proliferative melanoma cell state.

Recently, spatial transcript sequencing and lineage‐tracing analysis of a *Prrx1* ^+^ mesenchymal‐like cell population have demonstrated the different spatial distribution of distinct melanoma cell states in melanoma tumors [44, 56, 61]. Such cell states bestow melanoma cells the ability to switch to adapt to external cues. Indeed, Karras et al. demonstrated that “*Prrx1* ^+^‐metastatic initiating cells” change their identity when or before they reach the liver or lungs and express very low to undetectable *Prrx1* levels.

Hence, we investigate the expression of some markers of melanoma cell states that might carry out these biological processes, upon PRRX1 loss. We show that silencing *PRRX1* in melanoma cells led to the acquisition of some stemness features, such as spheroid formation, and increased expression of pluripotency transcription factors, such as NANOG. Furthermore, we observed a significant gain in the expression of NCSC markers p75NTR and L1CAM, concomitantly with a sustained expression of SOX10. In line with these findings, Li et al. [62] reported that p75NTR^+^ ESCC cells overexpressed NANOG, formed more self‐renewing spheres, and promoted anchorage‐independent growth compared with p75NTR^−^ cells. Acquisition of these properties might afford these cells several advantages for metastatic progression, and eventually therapy resistance. For instance, Boiko's lab previously demonstrated that within human melanomas, cells expressing p75NTR, represent the most aggressive tumor‐ and metastasis‐initiating cell population since its elevated levels can serve as a cell proliferative switch in melanoma‐initiating cells [21, 63]. Similarly, the expression of neural crest‐stem cell factors p75NTR and SOX10 in human melanoma correlates with high metastatic potential and worse patient prognosis [64].

Overall, our data support the notion that PRRX1 loss in melanoma cells promotes a phenotypic switch by inducing a reprogramming process towards a de‐differentiated phenotype favouring the reemergence/reinforcement of a neural crest‐like signature and counteracting the invasiveness associated with the EMT programme. This is not surprising given that Durand et al. reported that the A375 parental cell line displays an NCSC‐like phenotype, albeit with high ZEB1 expression levels [65]. These authors demonstrate that ZEB1 binds to the promoter of p75NTR and activates its expression in both NCSC‐like and mesenchymal populations of melanoma cells. Nevertheless, in our scenario, the activation of p75NTR cannot be directly attributed to ZEB1 since its levels are decreased upon depletion of PRRX1. We speculate whether the phenotypic switch caused by PRRX1 loss might affect the expression of p75NTR, which is also regulated by DNA methylation [62].

Thus, further research is necessary to decipher the mechanisms underlying the convergent up‐regulation of p75NTR and PRRX1 loss, as well as how it may impact the adoption of different melanoma cell states that compromise cell behavior throughout the metastatic progression.

On the other hand, the identity of the effector(s) required for the loss of PRRX1 expression in metastasis remains challenging. It is well known that, before or after tumor cell dissemination, some effectors or signals emanating from the tumor microenvironment may promote phenotype switching favouring or not the aggressiveness. For instance, Kasemeier‐Kulesa and Kulesa [66] have identified NGF as the signal within the chick embryonic neural crest microenvironment that allows reprogramming and sustains the transition of human metastatic melanoma to a neural crest cell‐like phenotype.

In addition, some microenvironment‐derived factors might induce the transcriptional activation of specific miRs that may target *PRRX1* expression, triggering a phenotypic switch. Several miRs have been identified as direct upstream regulators of PRRX1 in CRC, breast cancer, and even melanoma [32, 67, 68, 69]. However, the activation of their expression at the secondary sites needs to be further explored.

Our results highlight the importance of identifying factors that cause PRRX1silencing *in vivo*, which could be targeted to hinder the re‐emergence of NCSC‐like melanoma cell states, whose consequences worsen the prognosis of these patients.

## 5 Conclusion

PRRX1 plays a pleiotropic role in melanoma progression. While its expression in primary tumors sustains the mesenchymal‐like phenotype, the loss of its expression is required for the outgrowth of metastases. Our study reveals that low *PRRX1* expression in human metastatic samples is an independent prognostic predictor of reduced OS in melanoma patients, and sheds light on the plasticity of melanoma cells that, upon PRRX1 loss, allows the re‐emergence of NCSC‐like melanoma cell states critical for the outgrowth of metastasis.

We propose that early detection of PRRX1 loss in metastatic skin lesions of melanoma patients should be considered to adopt more specific therapies that may benefit these patients.

## Conflict of interest

The authors declare no conflict of interest.

## Author contributions

AF designed the experiments. JRF, SP, CC, JM (J Marcoval), JM (J Malvehy), and SP collected the clinical samples, analyzed the molecular and clinical‐pathological patterns, and assessed the datasets of patients. AV, RR, and EB performed the *in vitro* and JRF the *in vivo* experiments under the supervision of AF. RCM, RE, and JRF carried out biostatistical analyses. Moreover, AV, RCM, RE, JRF, and AF prepared the figures. The manuscript was written by AF and supervised by IF. All authors read and approved the final manuscript.

## Supporting information


**Fig. S1.** (A, B) Scatterplots illustrating the relationship between PRRX1 levels and Breslow score.
**Fig. S2.** (A–H) Assessment of *PRRX1* for Prognostic Prediction of cutaneous melanoma patients.
**Fig. S3.** (A, B) Bar charts showing the most significantly enriched genesets by NES in primary tumors and metastasis by pre‐ranked GSEA.
**Fig. S4.** Human melanoma cell lines express different levels of PRRX1 and TWIST1 transcription factors.
**Fig. S5.** PRRX1 silencing in the A375MM human melanoma cell line abrogates the expression of TWIST1.
**Fig. S6.** Representative images of extravasated A375MM's EGFP‐labeled tumor cells in the lungs of NSG mice.
**Fig. S7.** Representative macro images of paraffin‐embedded pieces from primary tumor xenografts.

**Table S1.** Clinical and histopathological characteristics of human melanoma samples (*cohort I*, *cohort II*, and *cohort III*).

**Table S2.** Correlation of *PRRX1* expression in primary tumors of cohort II with clinicopathological parameters.
**Table S3.** Plasmids used.
**Table S4.** Primary and Secondary antibodies used.
**Table S5.** Oligonucleotides used for qPCR.

**Table S6.** Selection of invasive genes used in ssGSEA (*n* = 200 genes) (related to Fig. 1E–G).

**Table S7.** TCGA‐SKCM metadata generated from TCGA‐SKCM dataset (including the invasiveness score calculated from ssGSEA).

**Table S8.** Hallmarks Gene sets from pre‐ranked GSEA in TCGA‐SKCM (invasive vs non invasive).

**Table S9.** EMT genes from pre‐ranked GSEA in TCGA‐SKCM (invasive vs non invasive) and Hallmarks Gene sets from GSEA (*PRRX1*Pearson correlation) in GSE22155; GSE65904; GSE116237.

**Table S10.** KRAS _UP genes from pre‐ranked GSEA in TCGA‐SKCM (*PRRX1* ^high^ vs *PRRX1* ^low^) and MAPK related Hallmarks Gene sets from GSEA (*PRRX1* Pearson correlation) in GSE22155; GSE65904; GSE116237.

## Data Availability

Research data are available upon request.
