# Supplementary material for: PRRX1 silencing is required for metastatic outgrowth in melanoma and is an independent prognostic of reduced survival in patients
Source: Mol Oncol. 2024 Jul 8;18(10):2471–94. doi: 10.1002/1878-0261.13688 (PMC11459042; doi:10.1002/1878-0261.13688)
Supplement: Supplementary file 1 — Fig. S1. (A, B) Scatterplots illustrating the relationship between PRRX1 levels and Breslow score. Fig. S2. (A–H) Assessment of PRRX1 for Prognostic Prediction of cutaneous melanoma patients. Fig. S3. (A, B) Bar charts showing the most significantly enriched genesets by NES in primary tumors and metastasis by pre‐ranked GSEA. Fig. S4. Human melanoma cell lines express different levels of PRRX1 and TWIST1 transcription factors. Fig. S5. PRRX1 silencing in the A375MM human melanoma cell line abrogates the expression of TWIST1. Fig. S6. Representative images of extravasated A375MM's EGFP‐labeled tumor cells in the lungs of NSG mice. Fig. S7. Representative macro images of paraffin‐embedded pieces from primary tumor xenografts. [file MOL2-18-2471-s005.pdf]

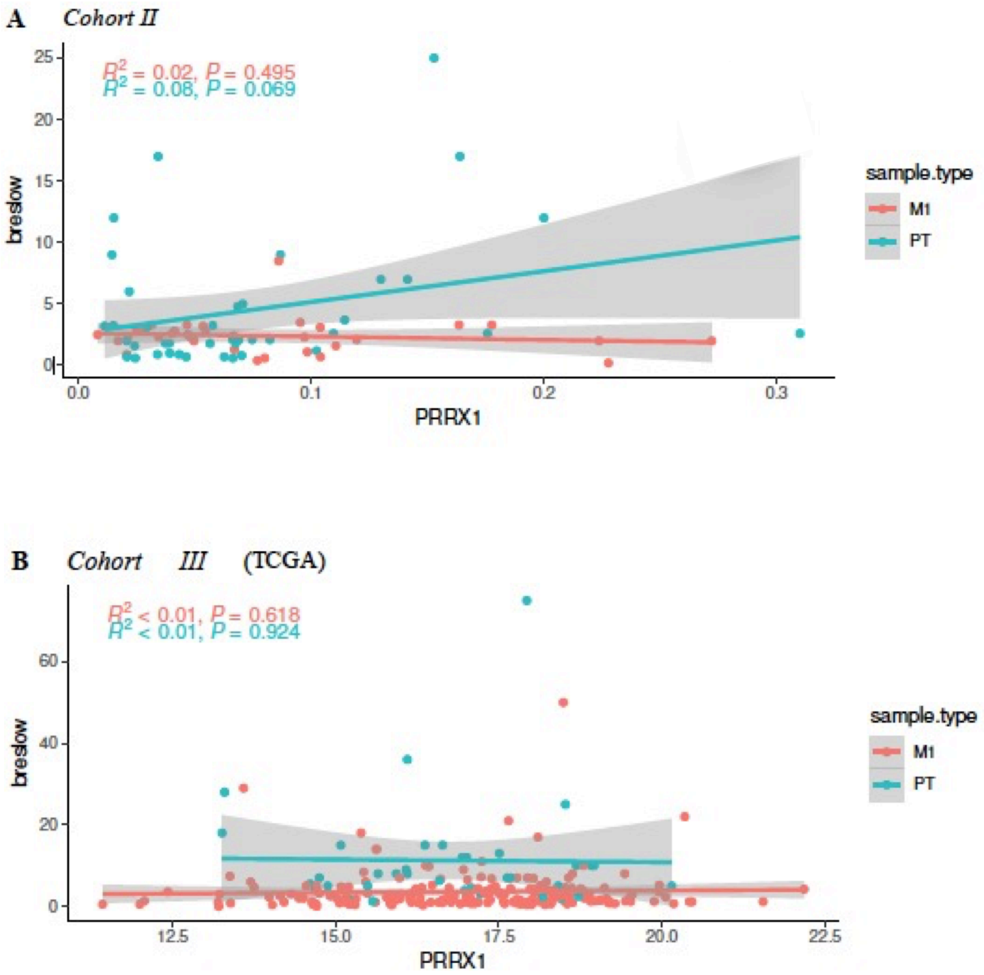

Supplementary Figure 1 A-B. Scatterplots illustrating the relationship between PRRX1 levels and Breslow score. Samples color-coded according to tumor status (M1 for metastatic and PT for primary tumor) in two distinct cohorts: A) *Cohort II*, and B) *Cohort III* (TCGA dataset). The coefficient of determination (R^2) and associated p-values (*P*) are reported to assess the strength and significance of the correlation.

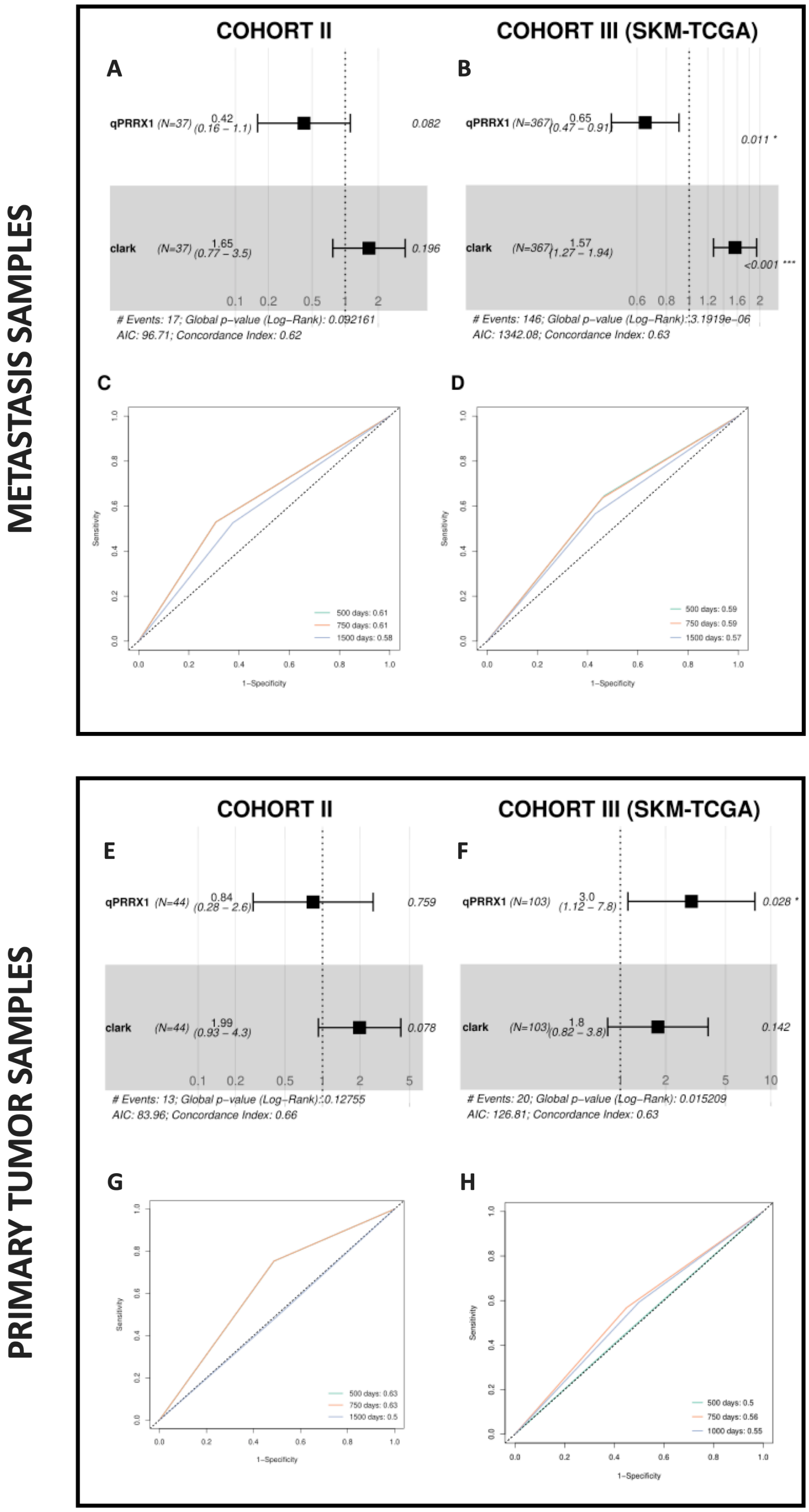

Supplementary Figure 2 A-H. Assessment of *PRRX1* for Prognostic Prediction of cutaneous melanoma patients. Results for the metastatic samples (*upper panels* A-D) and primary tumor samples (*bottom panels* E-H) were adjusted for the Clark score. The left panels show results for Cohort *II* analysis and the right panels for Cohort *III* (SKCM-TCGA). (A, B) and (E-F): Forest plots for the multivariate Cox regression models. HR together with 95% CIs are shown. Below each square are the p-values for each variable. (C-D) and (G-H): ROC curves for the time-dependent analysis on the indicated days. HR: Hazard ratio: N: sample size. CI: Confidence Interval. *P*: p-value. AUC: Area under the curve.

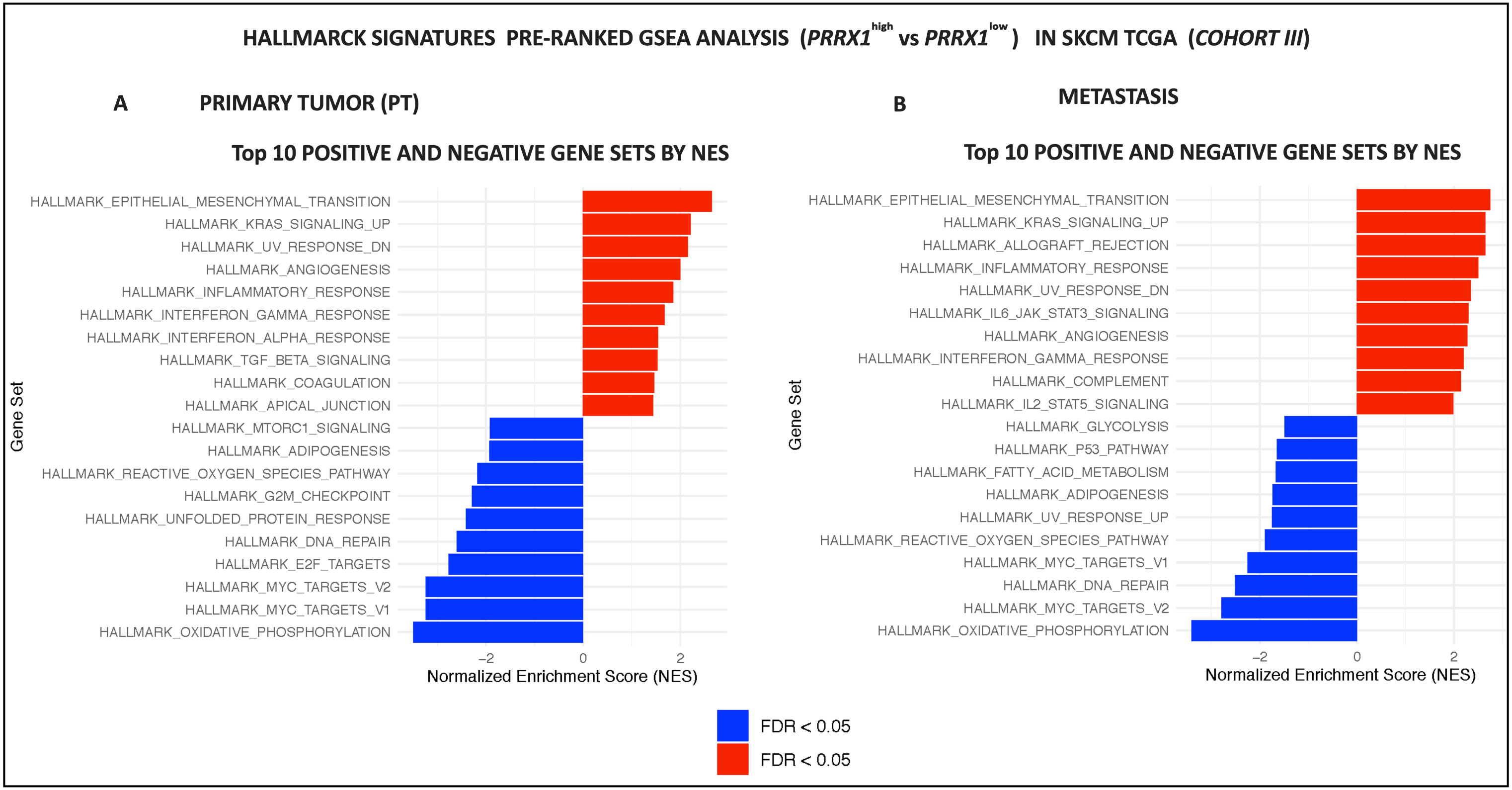

Supplementary Figure 3 A-B) Bar charts showing the most significantly enriched gene sets by NES in primary tumors (A), and metastasis (B) obtained by pre-ranked GSEA (high versus low *PRRX1* expression) in SKCM-TCGA dataset. Bars indicate the up-(positively correlated, in red) and down-pathways (negatively correlated, in blue), according to FDR. The KRAS_UP was the second of the 10 top gene sets positively correlated with high PRRX1 expression levels.

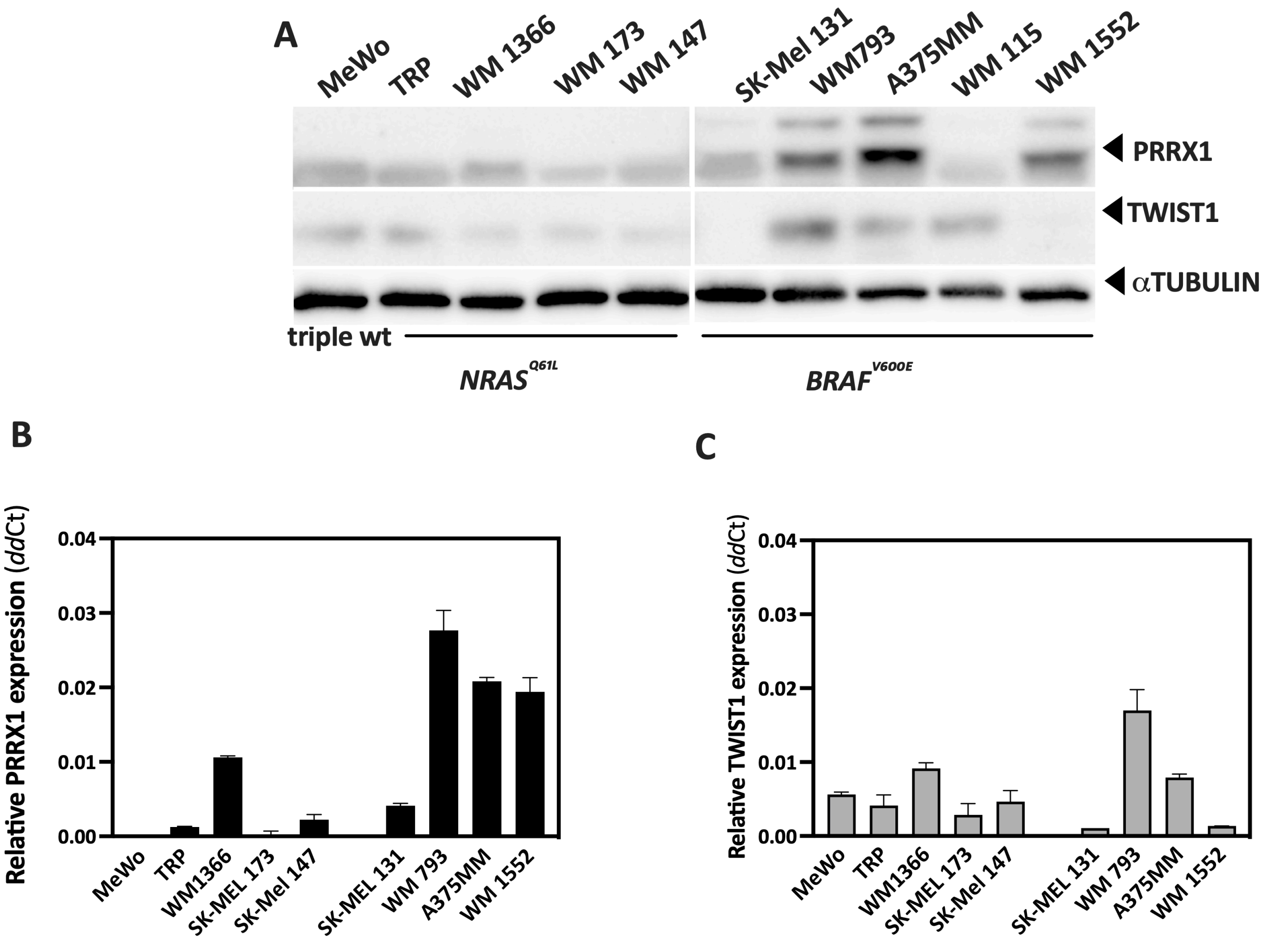

Supplementary Figure 4. Human melanoma cell lines express different levels of PRRX1 and TWIST1 transcription factors. (**A**) Immunoblot for PRRX1 and TWIST1 proteins in a panel of human melanoma cell lines. Cell lines are grouped based on *NRAS*^{Q61L} or *BRAF*^{V600E} mutations. MeWo cell line lacked hot-spot mutations in *NRAS*, *BRAF,* or *NF1* genes (triple *wt*). α-Tubulin was used as a loading control.

(**B**-**C**) Gene expression analysis of *PRRX1* and *TWIST1* mRNA relative to RP*L32* expression in a panel of melanoma cell lines. One representative of three independent experiments is shown and includes the mean ± SEM of technical triplicates.

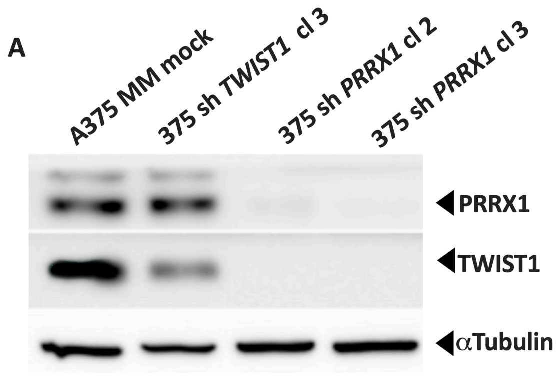

Supplementary Figure 5. PRRX1 silencing in the A375MM human melanoma cell line abrogates the expression of TWIST1 (**A**) Representative immunoblots showing expression of PRRX1 and TWIST 1 in A375MM control cells (mock) and upon PRRX1 or TWIST1 silencing by sh*PRRX1* or sh*TWIST1* respectively. α-Tubulin was used as a loading control.

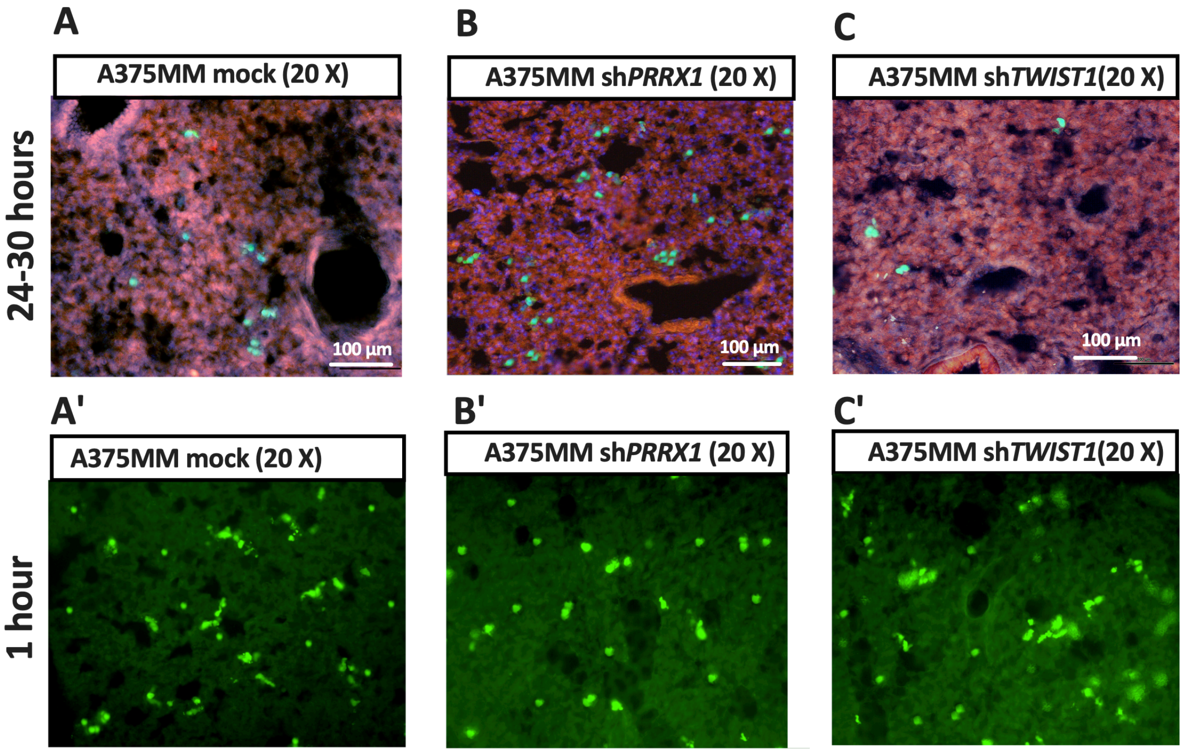

Supplementary Figure 6. Representative images of extravasated A375MM's EGFP-labelled tumor cells in the lungs of NSG mice. Images show the position of extravasated tumor cells (green) in sections of lung parenchyma (labeled with a Texas red lectin) 24 hours after intravenous injection of $2x10^6$ tumor cells. Nuclei were labeled with DAPI. *Upper* panels show images taken at 20X, scale bar 100 µm. *Bottom* panels: representative images showing the presence of tumor cells (green) in the lungs one hour after an injection of $2x10^6$ EGFP-labeled single cells.

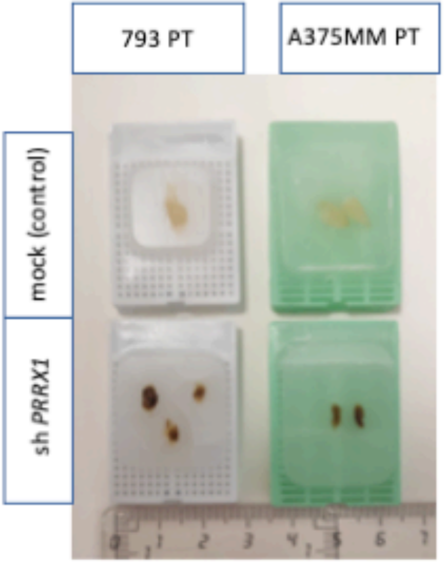

Supplementary Figure 7. Representative macroimages of paraffin-embedded primary tumors generated by either control or PRRX1-silenced cells.
