# Supplementary material for: PRRX1 silencing is required for metastatic outgrowth in melanoma and is an independent prognostic of reduced survival in patients
Source: Mol Oncol. 2024 Jul 8;18(10):2471–94. doi: 10.1002/1878-0261.13688 (PMC11459042; doi:10.1002/1878-0261.13688)
Supplement: Supplementary file 2 — Table S1. Clinical and histopathological characteristics of human melanoma samples (cohort I, cohort II, and cohort III). [file MOL2-18-2471-s001.pdf]

Supplementary Table ST1: Clinical and histopathological characteristics of human melanoma samples

COHORT I Primary Tumor		
	**Variable**	**n and (%)**
n=52		
	Gender	
	Male	24 (46.1)
	Female	28(53.4)
	Age of onset (Mean+/-SD)	62+/-13.98
	LMM	6(11.53)
	SSMM	40(76.92)
	NM	5(9.61)
	ALM	1(1.92)
	Clark	
	I	22(42.3)
	II-III	11(21.15)
	IV-V	19(36.53)
	Breslow (Mean+/-SD)	1.53+/-2.54
	REGRESSION	
	Present	NA
	Absent	NA
	ULCERATION	
	Present	5(9.6)
	Absent	47(90.38)
	VASCULAR INVASION	
	Present	4(7.69)
	Absent	47(90.3)
	SATELLITOSI	
	Present	2(3.84)
	Absent	50(96.15)
	Clinical Stage	
	in situ	23 (44.23)
	IA-IB	15(28.84)
	IIA-IIB	8(15.38)
	IIIA-IIIB	6(11.53)

COHORT II Primary Tumor		
	**Variable**	**n and (%)**
n=44		
	Gender	
	Male	28 (64)
	Female	16(36)
	Age of onset (Mean+/-SD)	69.55+/-13.3
	LMM	4(9.0)
	SSMM	35(79.5)
	NM	3(6.8)
	ALM	2(4.5)
	Clark	
	I	1(2.3)
	II-III	12(27.2)
	IV-V	31(70.4)
	Breslow (Mean+/-SD)	3.5+/-4.1
	REGRESSION	
	Present	NA
	Absent	NA
	ULCERATION	
	Present	16(36.36)
	Absent	28(63.6)
	VASCULAR INVASION	
	Present	3(6.1)
	Absent	46(93.9)
	SATELLITOSI	
	Present	3(6.8)
	Absent	41(93.18)
	Clinical Stage	
	in situ	2 (4.5)
	IA-IB	15(34.0)
	IIA-IIB	11(25)
	IIIA-IIIB	14(31.8)
	IV	2(4.5)
	Mitotic index	
	Mean+/-SD	4.69+/-6.61

COHORT III (SKCM-TCGA)Primary Tumor		
	**Variable**	**n and (%)**
n=103		
	Gender	
	Male	61 (59.2)
	Female	42(40.7)
	Age of onset (Mean+/-SD)	64.70+/-13.9
	LMM	1(0.97)
	SSMM	73(70.8)
	NM	16(15.5)
	ALM	2(1.9)
	NA	11(10.6)
	Clark	
	NA	28(27.1)
	I	0(0)
	II-III	15(14.5)
	IV-V	60(58.2)
	Breslow (Mean+/-SD)	11.7+/-12.7
	REGRESSION	
	Present	NA
	Absent	NA
	ULCERATION	
	Present	76(73.7)
	Absent	13(12.6)
	NA	14(13.5)
	VASCULAR INVASION	
	Present	NA
	Absent	NA
	SATELLITOSI	
	Present	NA
	Absent	NA
	Clinical Stage	
	in situ	0 (0)
	IA-IB	2(1.94)
	IIA-IIB-IIC	66(64.0)
	IIIA-IIIB	27(26.2)
	IV	3(2.9)
	NA	5(4.9)

COHORT II Metastases		
	**Variable**	**n and (%)**
n=37		
	Gender	
	Male	19 (51.35)
	Female	18(48.6)
	Age of onset (Mean+/-SD)	66.2+/-18.33
	Type of Metastasis	
	Cutaneous	37(100)
	Node	0(0)
	Visceral	0(0)

COHORT III Metastases		
	**Variable**	**n and (%)**
n= 368		
	Gender	
	Male	231 (62.8)
	Female	137(37.22)
	Age of onset (Mean+/-SD)	56,3+/-15.59
	Type of Metastasis	
	NA	2(0.5)
	Cutaneous	119(32.3)
	Node	210(57.0)
	Visceral	37(10)

COHORT II Primary Tumor and Metastasis		
	**Mutation**	**n and (%)**
	BRAF	21 (26.929
	BRAF+NF1	1(1.28)
	NRAS	19(24.36)
	NRAS+BRAF	1(1.28)
	Triple WT	14(17.95)
	Unknown	3

COHORT III Primary Tumor and Metastasis		
	**Mutation**	**n and (%)**
	BRAF	163
	BRAF+NF1	nd
	NRAS	79
	NRAS+BRAF	nd
	Triple WT	49
	Unknown	nd
