# Supplementary material for: PRRX1 silencing is required for metastatic outgrowth in melanoma and is an independent prognostic of reduced survival in patients
Source: Mol Oncol. 2024 Jul 8;18(10):2471–94. doi: 10.1002/1878-0261.13688 (PMC11459042; doi:10.1002/1878-0261.13688)
Supplement: Supplementary file 3 — Table S2. Correlation of PRRX1 expression in primary tumors of cohort II with clinicopathological parameters. Table S3. Plasmids used. Table S4. Primary and Secondary antibodies used. Table S5. Oligonucleotides used for qPCR. [file MOL2-18-2471-s008.pdf]

Supplementary Table ST2: PRRX1 Correlation of PRRX1 expression in primary tumors of cohort *II* with clinicopathological parameters

PRRX1 EXPRESSION	**Clinical stage AJCC 2017**	**Lymphatic invasion**
R Pearson	0.091	0.277
Significance	0.588	0.05*
number of samples	38	25

Supplementary Table ST3: Plasmids used

gene	vector	sequence	Antibiotic selection	reference
hPRRX1	pLKO.1 (lentiviral)	ATTCTTCAGAAAGAC TTTGGC (clone TRCN0000020644)	puromycin	RHS3979-9588052/201751761 shRNA Open Biosystems
hTWIST1	pLKO.1 (lentiviral)	TTCAGACTTCTATCAG AATGC (clone TRCN0000020539)	puromycin	RHS3979-9587949/201751656 shRNA Open Biosystems
EGFP	pLenti CMV GFP (lentiviral)	eGFP cDNA	puromycin	pLenti CMV GFP Addgene#17448

Supplementary Table ST4: Primary and secondary Antibodies used

PRIMARY ANTIBODIES	SOURCE	REFERENCE	Dilution
ß-Actin-HRP	Abcam	ab49900	1:25000 (WB)
FRA1 (D80B4)	Cell Signaling	5281	1:1000 (WB)
Phospho-MLC2 (thr18/Ser19)	Cell Signaling	3674	1:1000 (IF)
p44/42 MAP Kinase	Cell Signaling	9102	1:1000 (WB)
Phospho-p44/42 MAP Kinase	Cell Signaling	9101	1:1000 (WB)
PRRX1 (clone: OTI1E10)	Origen	TA803116	1:2000 (WB)
PRRX1	Sigma	HPA051084	1:250 (IHC)
SNAIL1 (SN9H2)	Cell Signaling	1:250	1:1000 (WB)
STAT3 (79D7)	Cell Signaling	1:1000	1:2000 (WB)
Phospho-STAT3 (Tyr705) (D3A7)	Cell Signaling	9145	1:2000 (WB)
α-Tubulin	Sigma	T6074	1:10000 (WB)
Tyrosinase	Santa Cruz	sc-15341	1:200 (WB)
TWIST (Twist2C1a)	Abcam	ab50887	1:250 (WB)
ZEB1 (D80D3)	Cell Signaling	3396	1:1000 (WB)
ZEB2	Abcam	223688	1:1000 (WB)
Phospho-Histone 3	Millipore	S10 clone 63-1c-8	1:2500 (IHC)
NGFR (p75NTR)	Sigma	HPA004765	1:500 (IHC antigen retrieval Citrate Buffer pH6) and (WB)

**SECONDARY ANTIBODIES**	**SOURCE**	**REFERENCE**	**Dilution**
anti-mouse HRP	Dako	P0260	1:10000 (WB)
Alexa Fluor 488 Goat anti-Rabbit	Life Technologies	A-11008	1:500 (IF)
anti-rabbit HRP	Dako	P0448	1:10000 (WB)

Supplementary Table ST5: Oligonucleotides used (qPCR)

Name	REFERENCE	
ABCB5	Forward	ATG TAC AGT GGC TCC GTT CC
	Reverse	ACA CGG CTG TTG TCA CCA TA
RPL32	Forward	GAT CTT GAT GCC CAA CAT TGG TTA TG
	Reverse	GCA CTT CCA GCT CCT TGA CG
NANOG	Forward	GCA GAG AAG AGT GTC G
	Reverse	AGC TGG GTG GAA GAG AAC ACA G
OCT-4	Forward	GAC AAC AAT GAA AAT CTT CAG GAG A
	Reverse	TTC TGG CGC CGG TTA CAG AAC CA
SOX2	Forward	TAC CTC TTC CTC CCA CTC CA
	Reverse	GGT AGT GCT GGG ACA TGT GA
TWIST1	Forward	CCG GAG ACC TAG ATG TCA TTG
	Reverse	CAC GCC CTG TTT CTT TGA AT
PRRX1	Forward	CTGATGCTTTTGTGCGAGAA
	Reverse	ACTTGGCTCTTCGGTTCTGA
FN1	Forward	CAATGTGGGTCCCTCTGTCT
	Reverse	CAGGCTGCAGTGTGGTAAAG
AXL	Forward	GGGCACGCAGGCTGAAGAAA
	Reverse	CTGTCCATCCCGAAGCCAATGT
NGFR	Forward	CAGGACAAGCAGAACACCGT
	Reverse	GGTGTGGACCGTGTAATCCA
L1CAM	Forward	CCACAGATGACATCAGCCTCAA
	Reverse	GGTCACACCCAGCTCTTCCTT
MITF	Forward	AGC CAT GCA GTC CGA AT
	Reverse	ACT GCT GCT CTT CAG CG
SOX9	Forward	GCGTATGAATCTCCTGGACC
	Reverse	GTCCTCCTCGCTCTCCTTCT
