# Supplementary material for: PRRX1 silencing is required for metastatic outgrowth in melanoma and is an independent prognostic of reduced survival in patients
Source: Mol Oncol. 2024 Jul 8;18(10):2471–94. doi: 10.1002/1878-0261.13688 (PMC11459042; doi:10.1002/1878-0261.13688)
Supplement: Supplementary file 4 — Table S6. Selection of invasive genes used in ssGSEA (n = 200 genes) (related to Fig. 1E–G). [file MOL2-18-2471-s007.pdf]

Supplementary Table 6: Selection of invasive genes used in ssGSEA

SOURCE	LIST OF GENES		(n=200)								
GSEA_MSIGDB:M 5930	ABI3BP	ACTA2	ADAM12	ANPEP	APLP1	AREG	BASP1	BDNF	BMP1	CADM1	CALD1
	CALU	CAP2	CAPG	CD44	CD59	CDH11	CDH2	CDH6	COL11A1	COL12A1	COL16A1
	COL1A1	COL1A2	COL4A1	COL4A2	COL5A1	COL5A2	COL5A3	COL6A2	COL6A3	COL7A1	COL8A2
	COLGALT1	COMP	COPA	CRLF1	CTHRC1	CXCL1	CXCL12	CXCL6	CXCL8	DAB2	DCN
	DPYSL3	DST	ECM1	ECM2	EDIL3	EFEMP2	ELN	EMP3	ENO2	FAP	FAS
	FBLN1	FBLN2	FBLN5	FBN1	FBN2	FERMT2	FGF2	FLNA	FMOD	FN1	FOXC2
	FSTL1	FSTL3	FUCA1	FZD8	GADD45A	GADD45B	GAS1	GEM	GJA1	GLIPR1	GPC1
	GPX7	GREM1	HTRA1	ID2	IGFBP2	IGFBP3	IGFBP4	IL15	IL32	IL6	INHBA
	ITGA2	ITGA5	ITGAV	ITGB1	ITGB3	ITGB5	LAMA1	LAMA2	LAMA3	LAMC1	LAMC2
	LGALS1	LOX	LOXL1	LOXL2	LRRC15	LUM	MAGEE1	MATN2	MATN3	MCM7	MEST
	MFAP5	MGP	MMP1	MMP14	MMP2	MMP3	MSX1	MXRA5	MYL9	MYLK	NID2
	NLRP1	NNMT	NOTCH2	NT5E	NTM	OXTR	PCOLCE2	PDGFRB	PDLIM4	PFN2	PLAUR
	P3H1	PCOLCE	PLOD1	PLOD2	PLOD3	PMEPA1	PPIB	PRRX1	PRSS22	PTHLH	PTX3
	PMP22	POSTN	PVR	QSOX1	RGS4	RHOB	SAT1	SCG2	SDC1	SDC4	SERPINE1
	SERPINE2	SERPINH1	SFRP1	SFRP4	SGCB	SGCD	SGCG	SLC6A8	SLIT3	SNAI2	SNTB1
	SPARC	SPOCK1	SPP1	TAGLN3	TFPI2	TGFB1	TGFBI	TGFBR3	THBS1	THBS2	THY1
	TIMP1	TIMP3	TNFAIP3	TNFRSF11B	TNFRSF12A	TPM1	TPM2	TPM4	VCAM1	VCAN	VEGFA
	VEGFC	VIM	WIPF1	WNT5A							
Rambow et al., 2018	CYR61	TNC	TGM2	BGN	SLIT2						
Pagliuca et al., 2022	AXL	ID3	AHR	TAZ							
