# Supplementary material for: PRRX1 silencing is required for metastatic outgrowth in melanoma and is an independent prognostic of reduced survival in patients
Source: Mol Oncol. 2024 Jul 8;18(10):2471–94. doi: 10.1002/1878-0261.13688 (PMC11459042; doi:10.1002/1878-0261.13688)
Supplement: Supplementary file 5 — Table S7. TCGA‐SKCM metadata generated from TCGA‐SKCM dataset (including the invasiveness score calculated from ssGSEA). [file MOL2-18-2471-s003.pdf]

Supplementary Table 7: TCGA-SKCM metadata

Barcode _ID and Sample_ID identification including 469 samples; PRRX1expression values (Log2) reported for each sample and PRRX1 score (High/Low expressed); Invasiveness score calculated by ssGSEA using the invasive signature vs non invasive (see Supp Table 7A); survival (OS status and OS time)

Barcode_ID	Sample_ID	PRRX1 (Log2)	PRRX1_group	Tissue	Invasiveness_score _n200 genes	OS.statu	OS.time
TCGA-3N-A9WB-06A	TCGA-3N-A9WB	16,7117563	Low	Metastasis	0,390565203	1	16,98361
TCGA-3N-A9WC-06A	TCGA-3N-A9WC	18,0086638	High	Metastasis	0,489706899	0	66,29508
TCGA-3N-A9WD-06A	TCGA-3N-A9WD	17,4340125	High	Metastasis	0,520172957	1	12,95082
TCGA-BF-A1PU-01A	TCGA-BF-A1PU	17,5689452	High	Primary Tumor	0,53920262	0	12,68852
TCGA-BF-A1PV-01A	TCGA-BF-A1PV	16,0810082	Low	Primary Tumor	0,513338027	0	0,459016
TCGA-BF-A1PX-01A	TCGA-BF-A1PX	17,6110836	High	Primary Tumor	0,602653854	1	9,245902
TCGA-BF-A1PZ-01A	TCGA-BF-A1PZ	14,4571737	Low	Primary Tumor	0,393897066	0	27,96721
TCGA-BF-A1Q0-01A	TCGA-BF-A1Q0	14,8756119	Low	Primary Tumor	0,546631664	0	27,2459
TCGA-BF-A3DJ-01A	TCGA-BF-A3DJ	18,2184424	High	Primary Tumor	0,574119206	0	15,21311
TCGA-BF-A3DL-01A	TCGA-BF-A3DL	14,7492651	Low	Primary Tumor	0,53813558	0	25,21311
TCGA-BF-A3DM-01A	TCGA-BF-A3DM	17,4742617	High	Primary Tumor	0,54533349	0	19,70492
TCGA-BF-A3DN-01A	TCGA-BF-A3DN	18,8638835	High	Primary Tumor	0,624088547	0	23,5082
TCGA-BF-A5EO-01A	TCGA-BF-A5EO	15,5702055	Low	Primary Tumor	0,552240759	0	23,04918
TCGA-BF-A5EP-01A	TCGA-BF-A5EP	17,8869856	High	Primary Tumor	0,561686767	0	10,98361
TCGA-BF-A5EQ-01A	TCGA-BF-A5EQ	17,0483856	High	Primary Tumor	0,592232972	0	10,59016
TCGA-BF-A5ER-01A	TCGA-BF-A5ER	14,2943186	Low	Primary Tumor	0,468059793	0	10,72131
TCGA-BF-A5ES-01A	TCGA-BF-A5ES	16,1467678	Low	Primary Tumor	0,540848962	0	16,06557
TCGA-BF-A9VF-01A	TCGA-BF-A9VF	13,3545796	Low	Primary Tumor	0,362946129	0	14,42623
TCGA-BF-AAOU-01A	TCGA-BF-AAOU	18,067316	High	Primary Tumor	0,619223405	0	15,60656
TCGA-BF-AAOX-01A	TCGA-BF-AAOX	14,6642811	Low	Primary Tumor	0,358783378	0	14,55738
TCGA-BF-AAP0-06A	TCGA-BF-AAP0	18,4249231	High	Metastasis	0,598857329	0	14,88525
TCGA-BF-AAP1-01A	TCGA-BF-AAP1	14,2827992	Low	Primary Tumor	0,404239247	0	13,40984
TCGA-BF-AAP2-01A	TCGA-BF-AAP2	14,920831	Low	Primary Tumor	0,381813683	0	13,27869
TCGA-BF-AAP4-01A	TCGA-BF-AAP4	15,8737573	Low	Primary Tumor	0,480840482	0	10,98361
TCGA-BF-AAP6-01A	TCGA-BF-AAP6	17,2669285	High	Primary Tumor	0,52022919	0	10,65574
TCGA-BF-AAP7-01A	TCGA-BF-AAP7	16,8056786	High	Primary Tumor	0,584524669	0	10,42623
TCGA-BF-AAP8-01A	TCGA-BF-AAP8	14,6565207	Low	Primary Tumor	0,365214811	0	14,65574
TCGA-D3-A1Q1-06A	TCGA-D3-A1Q1	14,0287919	Low	Metastasis	0,395998991	1	16,52459
TCGA-D3-A1Q3-06A	TCGA-D3-A1Q3	16,3179231	Low	Metastasis	0,460770832	1	16,62295
TCGA-D3-A1Q4-06A	TCGA-D3-A1Q4	19,0363886	High	Metastasis	0,805852201	0	111,7377
TCGA-D3-A1Q5-06A	TCGA-D3-A1Q5	17,8712532	High	Metastasis	0,718133288	1	112,2623
TCGA-D3-A1Q6-06A	TCGA-D3-A1Q6	18,4938569	High	Metastasis	0,560524566	1	71,60656
TCGA-D3-A1Q7-06A	TCGA-D3-A1Q7	17,5894478	High	Metastasis	0,60767536	0	132,8852
TCGA-D3-A1Q8-06A	TCGA-D3-A1Q8	19,5021439	High	Metastasis	0,779707388	1	28
TCGA-D3-A1Q9-06A	TCGA-D3-A1Q9	15,4633263	Low	Metastasis	0,479600704	1	31,5082
TCGA-D3-A1QA-06A	TCGA-D3-A1QA	15,2928362	Low	Metastasis	0,361294078	0	90,65574
TCGA-D3-A1QB-06A	TCGA-D3-A1QB	14,5168265	Low	Metastasis	0,439607604	0	95,47541
TCGA-D3-A2J6-06A	TCGA-D3-A2J6	15,4899089	Low	Metastasis	0,490889048	1	43,31148
TCGA-D3-A2J7-06A	TCGA-D3-A2J7	17,0235303	High	Metastasis	0,589060339	1	102,8197
TCGA-D3-A2J8-06A	TCGA-D3-A2J8	18,5043725	High	Metastasis	0,655429153	1	65,31148
TCGA-D3-A2J9-06A	TCGA-D3-A2J9	17,6502934	High	Metastasis	0,521532315	1	23,70492
TCGA-D3-A2JA-06A	TCGA-D3-A2JA	19,8803487	High	Metastasis	0,770979117	0	115,2131
TCGA-D3-A2JB-06A	TCGA-D3-A2JB	16,5442337	Low	Metastasis	0,508848764	1	167,541
TCGA-D3-A2JC-06A	TCGA-D3-A2JC	19,1991146	High	Metastasis	0,565385809	0	86,52459
TCGA-D3-A2JD-06A	TCGA-D3-A2JD	15,392264	Low	Metastasis	0,537826509	1	11,83607
TCGA-D3-A2JE-06A	TCGA-D3-A2JE	15,4513441	Low	Metastasis	0,396323475	1	27,57377
TCGA-D3-A2JF-06A	TCGA-D3-A2JF	16,3412032	Low	Metastasis	0,532603127	0	61,90164
TCGA-D3-A2JG-06A	TCGA-D3-A2JG	18,3208548	High	Metastasis	0,498840092	1	113,2131
TCGA-D3-A2JH-06A	TCGA-D3-A2JH	16,158911	Low	Metastasis	0,455189186	0	41,96721
TCGA-D3-A2JK-06A	TCGA-D3-A2JK	17,9763177	High	Metastasis	0,553501328	1	12,06557
TCGA-D3-A2JL-06A	TCGA-D3-A2JL	15,217986	Low	Metastasis	0,416414576	0	171,1148
TCGA-D3-A2JN-06A	TCGA-D3-A2JN	19,2905556	High	Metastasis	0,690899078	1	66,29508
TCGA-D3-A2JO-06A	TCGA-D3-A2JO	15,6095439	Low	Metastasis	0,486644301	0	65,90164
TCGA-D3-A2JP-06A	TCGA-D3-A2JP	16,6709684	Low	Metastasis	0,5424039	0	59,40984
TCGA-D3-A3BZ-06A	TCGA-D3-A3BZ	18,5167484	High	Metastasis	0,756076357	0	130,3607
TCGA-D3-A3C1-06A	TCGA-D3-A3C1	18,0716027	High	Metastasis	0,564177412	0	NA
TCGA-D3-A3C3-06A	TCGA-D3-A3C3	16,70056	Low	Metastasis	0,371145151	0	NA
TCGA-D3-A3C6-06A	TCGA-D3-A3C6	18,971623	High	Metastasis	0,532075746	1	57,90164
TCGA-D3-A3C7-06A	TCGA-D3-A3C7	17,8779925	High	Metastasis	0,629273864	0	46,85246
TCGA-D3-A3C8-06A	TCGA-D3-A3C8	17,5267901	High	Metastasis	0,625364498	0	46,19672
TCGA-D3-A3CB-06A	TCGA-D3-A3CB	17,7823619	High	Metastasis	0,5227463	0	166,0656
TCGA-D3-A3CC-06A	TCGA-D3-A3CC	17,2272476	High	Metastasis	0,566733542	0	86,68852

TCGA-D3-A3CE-06A	TCGA-D3-A3CE	18,2409294	High	Metastasis	0,495648104	1	60,06557
TCGA-D3-A3CF-06A	TCGA-D3-A3CF	16,9602644	High	Metastasis	0,575833925	1	24,45902
TCGA-D3-A3ML-06A	TCGA-D3-A3ML	13,9824125	Low	Metastasis	0,32443981	1	13,83607
TCGA-D3-A3MO-06A	TCGA-D3-A3MO	19,0965118	High	Metastasis	0,586086658	1	9,311475
TCGA-D3-A3MR-06A	TCGA-D3-A3MR	18,6275637	High	Metastasis	0,590396428	0	103,3115
TCGA-D3-A3MU-06A	TCGA-D3-A3MU	17,587884	High	Metastasis	0,641626666	0	39,63934
TCGA-D3-A3MV-06A	TCGA-D3-A3MV	18,3087258	High	Metastasis	0,574245103	0	45,18033
TCGA-D3-A51E-06A	TCGA-D3-A51E	14,6377287	Low	Metastasis	0,445006622	0	174,3607
TCGA-D3-A51F-06A	TCGA-D3-A51F	18,5656033	High	Metastasis	0,685072581	0	55,57377
TCGA-D3-A51G-06A	TCGA-D3-A51G	20,6981601	High	Metastasis	0,70291962	0	NA
TCGA-D3-A51H-06A	TCGA-D3-A51H	17,3275323	High	Metastasis	0,45647794	0	56,19672
TCGA-D3-A51J-06A	TCGA-D3-A51J	16,3207819	Low	Metastasis	0,478328724	0	144,7213
TCGA-D3-A51K-06A	TCGA-D3-A51K	13,2128095	Low	Metastasis	0,325288796	0	32,85246
TCGA-D3-A51N-06A	TCGA-D3-A51N	18,8395038	High	Metastasis	0,507631554	0	22,55738
TCGA-D3-A51R-06A	TCGA-D3-A51R	18,5728449	High	Metastasis	0,517418387	0	63,63934
TCGA-D3-A51T-06A	TCGA-D3-A51T	18,0211569	High	Metastasis	0,445473995	0	26,81967
TCGA-D3-A5GL-06A	TCGA-D3-A5GL	17,3658295	High	Metastasis	0,471912143	0	125,4426
TCGA-D3-A5GN-06A	TCGA-D3-A5GN	18,5524626	High	Metastasis	0,677048679	0	135,377
TCGA-D3-A5GO-06A	TCGA-D3-A5GO	18,7627228	High	Metastasis	0,541576189	0	137,541
TCGA-D3-A5GR-06A	TCGA-D3-A5GR	18,5449221	High	Metastasis	0,643927106	0	177,8361
TCGA-D3-A5GS-06A	TCGA-D3-A5GS	16,9060529	High	Metastasis	0,542614392	0	18,13115
TCGA-D3-A5GT-01A	TCGA-D3-A5GT	14,8615884	Low	Primary Tumor	0,329651783	0	15,96721
TCGA-D3-A5GU-06A	TCGA-D3-A5GU	15,2418439	Low	Metastasis	0,370991936	0	124,8525
TCGA-D3-A8GB-06A	TCGA-D3-A8GB	17,5629564	High	Metastasis	0,670561096	1	30,7541
TCGA-D3-A8GC-06A	TCGA-D3-A8GC	15,0479855	Low	Metastasis	0,471898108	1	79,37705
TCGA-D3-A8GD-06A	TCGA-D3-A8GD	15,5644216	Low	Metastasis	0,438414744	0	23,54098
TCGA-D3-A8GE-06A	TCGA-D3-A8GE	16,1368201	Low	Metastasis	0,747147889	0	26,36066
TCGA-D3-A8GI-06A	TCGA-D3-A8GI	16,0269414	Low	Metastasis	0,642295468	1	58,36066
TCGA-D3-A8GJ-06A	TCGA-D3-A8GJ	18,0135303	High	Metastasis	0,473835404	0	240,7213
TCGA-D3-A8GK-06A	TCGA-D3-A8GK	13,7751445	Low	Metastasis	0,394476671	0	169,7377
TCGA-D3-A8GL-06A	TCGA-D3-A8GL	14,9285073	Low	Metastasis	0,352906272	1	88,88525
TCGA-D3-A8GM-06A	TCGA-D3-A8GM	16,5402912	Low	Metastasis	0,477939649	1	106,8525
TCGA-D3-A8GN-06A	TCGA-D3-A8GN	16,3539588	Low	Metastasis	0,570229907	0	160,5574
TCGA-D3-A8GO-06A	TCGA-D3-A8GO	17,5492656	High	Metastasis	0,555314152	1	NA
TCGA-D3-A8GP-06A	TCGA-D3-A8GP	16,5666315	Low	Metastasis	0,401108945	0	152,3279
TCGA-D3-A8GQ-06A	TCGA-D3-A8GQ	16,7219528	Low	Metastasis	0,557196829	1	28,98361
TCGA-D3-A8GR-06A	TCGA-D3-A8GR	14,6357263	Low	Metastasis	0,37609592	1	129,2787
TCGA-D3-A8GS-06A	TCGA-D3-A8GS	16,4760862	Low	Metastasis	0,322082455	1	116,8525
TCGA-D3-A8GV-06A	TCGA-D3-A8GV	19,3158141	High	Metastasis	0,444039643	1	167,2459
TCGA-D9-A148-06A	TCGA-D9-A148	15,4787426	Low	Metastasis	0,453939101	0	151,1148
TCGA-D9-A149-06A	TCGA-D9-A149	15,8528255	Low	Metastasis	0,433402028	0	54,52459
TCGA-D9-A1JW-06A	TCGA-D9-A1JW	14,7174241	Low	Metastasis	0,429688466	0	3,639344
TCGA-D9-A1JX-06A	TCGA-D9-A1JX	15,9738364	Low	Metastasis	0,448844693	1	7,081967
TCGA-D9-A1X3-06A	TCGA-D9-A1X3	17,0772158	High	Metastasis	0,472019578	0	18,06557
TCGA-D9-A3Z1-06A	TCGA-D9-A3Z1	15,9262674	Low	Metastasis	0,440103672	1	15,34426
TCGA-D9-A3Z3-06A	TCGA-D9-A3Z3	16,2159742	Low	Metastasis	0,551056886	0	22,22951
TCGA-D9-A3Z4-01A	TCGA-D9-A3Z4	16,9443463	High	Primary Tumor	0,501859443	1	17,01639
TCGA-D9-A4Z2-01A	TCGA-D9-A4Z2	18,5217555	High	Primary Tumor	0,528632487	1	6,229508
TCGA-D9-A4Z3-01A	TCGA-D9-A4Z3	17,9346242	High	Primary Tumor	0,414823868	0	16,55738
TCGA-D9-A4Z5-01A	TCGA-D9-A4Z5	16,2269816	Low	Primary Tumor	0,563605629	0	7,147541
TCGA-D9-A4Z6-06A	TCGA-D9-A4Z6	16,5960981	Low	Metastasis	0,483190357	1	18,39344
TCGA-D9-A6E9-06A	TCGA-D9-A6E9	15,266728	Low	Metastasis	0,470957894	0	9,868852
TCGA-D9-A6EA-06A	TCGA-D9-A6EA	13,2357324	Low	Metastasis	0,428499004	0	25,11475
TCGA-D9-A6EC-06A	TCGA-D9-A6EC	14,8322829	Low	Metastasis	0,415777989	0	77,34426
TCGA-D9-A6EG-06A	TCGA-D9-A6EG	19,3410574	High	Metastasis	0,437026241	1	22,88525
TCGA-DA-A1HV-06A	TCGA-DA-A1HV	15,0047164	Low	Metastasis	0,442542705	0	76,36066
TCGA-DA-A1HW-06A	TCGA-DA-A1HW	17,1285173	High	Metastasis	0,468385029	0	26,88525
TCGA-DA-A1HY-06A	TCGA-DA-A1HY	20,4602637	High	Metastasis	0,689456946	0	144,4918
TCGA-DA-A1I0-06A	TCGA-DA-A1I0	15,0980544	Low	Metastasis	0,555496093	1	20,32787
TCGA-DA-A1I1-06A	TCGA-DA-A1I1	19,7011999	High	Metastasis	0,623615006	0	206,918
TCGA-DA-A1I2-06A	TCGA-DA-A1I2	19,8307717	High	Metastasis	0,489170167	1	176,0656
TCGA-DA-A1I4-06A	TCGA-DA-A1I4	17,5084141	High	Metastasis	0,672905123	1	35,83607
TCGA-DA-A1I5-06A	TCGA-DA-A1I5	18,2965984	High	Metastasis	0,619460234	0	119,6721
TCGA-DA-A1I7-06A	TCGA-DA-A1I7	20,3178517	High	Metastasis	0,745267775	0	73,63934
TCGA-DA-A1I8-06A	TCGA-DA-A1I8	19,427834	High	Metastasis	0,585211436	1	53,77049
TCGA-DA-A1IA-06A	TCGA-DA-A1IA	12,0678253	Low	Metastasis	0,377192019	1	65,7377
TCGA-DA-A1IB-06A	TCGA-DA-A1IB	17,7839446	High	Metastasis	0,553272058	0	27,04918
TCGA-DA-A1IC-06A	TCGA-DA-A1IC	16,818228	High	Metastasis	0,494370137	1	67,90164
TCGA-DA-A3F2-06A	TCGA-DA-A3F2	18,1286735	High	Metastasis	0,695926975	1	33,83607
TCGA-DA-A3F3-06A	TCGA-DA-A3F3	15,8736776	Low	Metastasis	0,359606542	1	10,45902

TCGA-DA-A3F5-06A	TCGA-DA-A3F5	18,2088185	High	Metastasis	0,621020833	1	225,3443
TCGA-DA-A3F8-06A	TCGA-DA-A3F8	15,9921958	Low	Metastasis	0,522544839	0	43,2459
TCGA-DA-A95V-06A	TCGA-DA-A95V	15,856508	Low	Metastasis	0,413814134	0	71,90164
TCGA-DA-A95W-06A	TCGA-DA-A95W	17,1158794	High	Metastasis	0,337155253	0	25,08197
TCGA-DA-A95X-06A	TCGA-DA-A95X	17,9151716	High	Metastasis	0,53851902	0	61,63934
TCGA-DA-A95Y-06A	TCGA-DA-A95Y	13,7632103	Low	Metastasis	0,368605328	1	14,09836
TCGA-DA-A95Z-06A	TCGA-DA-A95Z	17,1840896	High	Metastasis	0,337085849	0	12,98361
TCGA-DA-A960-01A	TCGA-DA-A960	15,8591394	Low	Primary Tumor	0,283575601	0	26,36066
TCGA-EB-A1NK-01A	TCGA-EB-A1NK	16,1024408	Low	Primary Tumor	0,573277985	0	34,06557
TCGA-EB-A24C-01A	TCGA-EB-A24C	18,9162286	High	Primary Tumor	0,523459981	0	20,72131
TCGA-EB-A24D-01A	TCGA-EB-A24D	16,1000252	Low	Primary Tumor	0,450962595	0	21,14754
TCGA-EB-A299-01A	TCGA-EB-A299	18,452751	High	Primary Tumor	0,602394809	0	12,39344
TCGA-EB-A3HV-01A	TCGA-EB-A3HV	17,5080019	High	Primary Tumor	0,634033032	0	1,278689
TCGA-EB-A3XB-01A	TCGA-EB-A3XB	15,3800749	Low	Primary Tumor	0,464288106	0	26,09836
TCGA-EB-A3XC-01A	TCGA-EB-A3XC	16,2802681	Low	Primary Tumor	0,54142999	0	21,31148
TCGA-EB-A3XD-01A	TCGA-EB-A3XD	17,2803428	High	Primary Tumor	0,609345453	0	38,03279
TCGA-EB-A3XE-01A	TCGA-EB-A3XE	17,216081	High	Primary Tumor	0,571107209	0	5,901639
TCGA-EB-A3XF-01A	TCGA-EB-A3XF	18,6913138	High	Primary Tumor	0,501672159	0	9,114754
TCGA-EB-A3Y6-01A	TCGA-EB-A3Y6	17,0773356	High	Primary Tumor	0,55785079	0	4,131148
TCGA-EB-A3Y7-01A	TCGA-EB-A3Y7	14,6813964	Low	Primary Tumor	0,407224015	1	10,68852
TCGA-EB-A41A-01A	TCGA-EB-A41A	14,7457782	Low	Primary Tumor	0,421252888	0	8,590164
TCGA-EB-A41B-01A	TCGA-EB-A41B	13,3036084	Low	Primary Tumor	0,273305382	0	9,540984
TCGA-EB-A42Y-01A	TCGA-EB-A42Y	20,1467311	High	Primary Tumor	0,542413126	1	23,63934
TCGA-EB-A42Z-01A	TCGA-EB-A42Z	18,9586646	High	Primary Tumor	0,588020421	0	14,45902
TCGA-EB-A430-01A	TCGA-EB-A430	13,2592529	Low	Primary Tumor	0,407556582	0	NA
TCGA-EB-A431-01A	TCGA-EB-A431	15,0797059	Low	Primary Tumor	0,365120883	0	18,62295
TCGA-EB-A44N-01A	TCGA-EB-A44N	18,4080475	High	Primary Tumor	0,612209186	1	6,721311
TCGA-EB-A44O-01A	TCGA-EB-A44O	15,4903029	Low	Primary Tumor	0,422128552	0	2,655738
TCGA-EB-A44P-01A	TCGA-EB-A44P	17,6107467	High	Primary Tumor	0,498076377	0	24,29508
TCGA-EB-A44Q-06A	TCGA-EB-A44Q	17,3382183	High	Metastasis	0,396075879	0	13,83607
TCGA-EB-A44R-06A	TCGA-EB-A44R	16,4382281	Low	Metastasis	0,464221452	1	10,32787
TCGA-EB-A4IQ-01A	TCGA-EB-A4IQ	16,3710596	Low	Primary Tumor	0,494395291	1	20,85246
TCGA-EB-A4IS-01A	TCGA-EB-A4IS	17,7275904	High	Primary Tumor	0,61602667	0	25,37705
TCGA-EB-A4OY-01A	TCGA-EB-A4OY	14,0039279	Low	Primary Tumor	0,335879864	0	32,03279
TCGA-EB-A4OZ-01A	TCGA-EB-A4OZ	16,7198675	Low	Primary Tumor	0,566243791	0	20,32787
TCGA-EB-A4P0-01A	TCGA-EB-A4P0	18,2621751	High	Primary Tumor	0,474917401	1	10,68852
TCGA-EB-A4XL-01A	TCGA-EB-A4XL	17,6251194	High	Primary Tumor	0,527264755	0	25,47541
TCGA-EB-A51B-01A	TCGA-EB-A51B	12,9311276	Low	Primary Tumor	0,39016041	0	30,52459
TCGA-EB-A550-01A	TCGA-EB-A550	16,0308129	Low	Primary Tumor	0,573285282	1	8,655738
TCGA-EB-A551-01A	TCGA-EB-A551	16,4121359	Low	Primary Tumor	0,524145629	0	19,34426
TCGA-EB-A553-01A	TCGA-EB-A553	17,3004137	High	Primary Tumor	0,474535798	0	7,409836
TCGA-EB-A57M-01A	TCGA-EB-A57M	17,4343322	High	Primary Tumor	0,683410127	1	15,47541
TCGA-EB-A5FP-01A	TCGA-EB-A5FP	15,6461375	Low	Primary Tumor	0,41753913	1	14,88525
TCGA-EB-A5KH-06A	TCGA-EB-A5KH	14,6039756	Low	Metastasis	0,459401629	1	20,29508
TCGA-EB-A5SE-01A	TCGA-EB-A5SE	17,283081	High	Primary Tumor	0,443243891	1	13,14754
TCGA-EB-A5SF-01A	TCGA-EB-A5SF	15,5387005	Low	Primary Tumor	0,550329021	1	12,09836
TCGA-EB-A5SG-06A	TCGA-EB-A5SG	17,1585537	High	Metastasis	0,55913336	0	68,06557
TCGA-EB-A5SH-06A	TCGA-EB-A5SH	14,221098	Low	Metastasis	0,366156423	0	53,86885
TCGA-EB-A5UL-06A	TCGA-EB-A5UL	15,2347354	Low	Metastasis	0,401394599	0	29,21311
TCGA-EB-A5UM-01A	TCGA-EB-A5UM	17,067721	High	Primary Tumor	0,400285012	0	25,54098
TCGA-EB-A5UN-06A	TCGA-EB-A5UN	13,9066323	Low	Metastasis	0,534470525	0	58,7541
TCGA-EB-A5VU-01A	TCGA-EB-A5VU	16,1346774	Low	Primary Tumor	0,518756359	1	10,52459
TCGA-EB-A5VV-06A	TCGA-EB-A5VV	18,0808997	High	Metastasis	0,434217746	0	7,016393
TCGA-EB-A6L9-06A	TCGA-EB-A6L9	16,1965534	Low	Metastasis	0,531441287	0	36,36066
TCGA-EB-A6QY-01A	TCGA-EB-A6QY	15,3459729	Low	Primary Tumor	0,403664428	0	12,52459
TCGA-EB-A6QZ-01A	TCGA-EB-A6QZ	16,7678943	Low	Primary Tumor	0,493456688	1	11,54098
TCGA-EB-A6R0-01A	TCGA-EB-A6R0	18,1309663	High	Primary Tumor	0,383172567	1	19,93443
TCGA-EB-A82B-01A	TCGA-EB-A82B	15,7693572	Low	Primary Tumor	0,507349576	0	12,78689
TCGA-EB-A82C-01A	TCGA-EB-A82C	15,9667204	Low	Primary Tumor	0,482682314	0	0,557377
TCGA-EB-A85I-01A	TCGA-EB-A85I	17,6313691	High	Primary Tumor	0,564594403	0	11,86885
TCGA-EB-A85J-01A	TCGA-EB-A85J	14,1690725	Low	Primary Tumor	0,370285198	0	11,80328
TCGA-EB-A97M-01A	TCGA-EB-A97M	16,3920751	Low	Primary Tumor	0,459226336	0	13,57377
TCGA-EE-A17X-06A	TCGA-EE-A17X	15,8281005	Low	Metastasis	0,370075609	1	29,7377
TCGA-EE-A17Y-06A	TCGA-EE-A17Y	18,891323	High	Metastasis	0,737551802	1	27,14754
TCGA-EE-A17Z-06A	TCGA-EE-A17Z	13,5869939	Low	Metastasis	0,402723776	1	8,622951
TCGA-EE-A180-06A	TCGA-EE-A180	17,445736	High	Metastasis	0,438343982	1	94,72131
TCGA-EE-A181-06A	TCGA-EE-A181	14,1539034	Low	Metastasis	0,351359553	1	33,63934
TCGA-EE-A182-06A	TCGA-EE-A182	15,6256497	Low	Metastasis	0,535975135	1	14,65574
TCGA-EE-A183-06A	TCGA-EE-A183	15,3946806	Low	Metastasis	0,522904205	1	26,81967
TCGA-EE-A184-06A	TCGA-EE-A184	15,0203348	Low	Metastasis	0,408971872	1	67,96721

TCGA-EE-A185-06A	TCGA-EE-A185	13,6961235	Low	Metastasis	0,380023977	1	4,95082
TCGA-EE-A20B-06A	TCGA-EE-A20B	17,0530747	High	Metastasis	0,678749474	0	133,4426
TCGA-EE-A20C-06A	TCGA-EE-A20C	11,9954317	Low	Metastasis	0,311802602	1	150,8525
TCGA-EE-A20F-06A	TCGA-EE-A20F	15,6083222	Low	Metastasis	0,484375688	0	91,31148
TCGA-EE-A20H-06A	TCGA-EE-A20H	16,9184395	High	Metastasis	0,509106823	1	167,8033
TCGA-EE-A20I-06A	TCGA-EE-A20I	16,9956638	High	Metastasis	0,546271263	1	13,5082
TCGA-EE-A29A-06A	TCGA-EE-A29A	18,3430596	High	Metastasis	0,67773754	1	63,18033
TCGA-EE-A29B-06A	TCGA-EE-A29B	15,9515486	Low	Metastasis	0,317218224	1	84,85246
TCGA-EE-A29C-06A	TCGA-EE-A29C	17,2879412	High	Metastasis	0,633880679	1	78,7541
TCGA-EE-A29D-06A	TCGA-EE-A29D	14,7531609	Low	Metastasis	0,451142841	1	13,93443
TCGA-EE-A29E-06A	TCGA-EE-A29E	13,3822943	Low	Metastasis	0,457782815	0	63,60656
TCGA-EE-A29G-06A	TCGA-EE-A29G	16,9771968	High	Metastasis	0,54750885	1	71,86885
TCGA-EE-A29H-06A	TCGA-EE-A29H	17,9370386	High	Metastasis	0,480898595	0	64,45902
TCGA-EE-A29L-06A	TCGA-EE-A29L	13,2030281	Low	Metastasis	0,294879562	1	2,590164
TCGA-EE-A29M-06A	TCGA-EE-A29M	15,0016102	Low	Metastasis	0,357937441	0	56,68852
TCGA-EE-A29N-06A	TCGA-EE-A29N	16,7423493	Low	Metastasis	0,518262019	1	18,55738
TCGA-EE-A29P-06A	TCGA-EE-A29P	15,8359195	Low	Metastasis	0,506638733	0	56,2623
TCGA-EE-A29Q-06A	TCGA-EE-A29Q	17,2777565	High	Metastasis	0,445062732	1	66,55738
TCGA-EE-A29R-06A	TCGA-EE-A29R	19,5202732	High	Metastasis	0,638334885	0	14,42623
TCGA-EE-A29S-06A	TCGA-EE-A29S	17,0953794	High	Metastasis	0,589674001	1	61,11475
TCGA-EE-A29T-06A	TCGA-EE-A29T	19,9571564	High	Metastasis	0,500077614	0	368,918
TCGA-EE-A29V-06A	TCGA-EE-A29V	14,7149234	Low	Metastasis	0,488705164	1	25,80328
TCGA-EE-A29W-06A	TCGA-EE-A29W	18,8080817	High	Metastasis	0,711689503	0	194,4918
TCGA-EE-A29X-06A	TCGA-EE-A29X	15,783095	Low	Metastasis	0,549695169	1	17,86885
TCGA-EE-A2A0-06A	TCGA-EE-A2A0	19,4436861	High	Metastasis	0,732760805	1	46,68852
TCGA-EE-A2A1-06A	TCGA-EE-A2A1	17,7816504	High	Metastasis	0,622313713	0	115,6393
TCGA-EE-A2A2-06A	TCGA-EE-A2A2	15,1376919	Low	Metastasis	0,37443129	0	59,47541
TCGA-EE-A2A5-06A	TCGA-EE-A2A5	17,8358416	High	Metastasis	0,551835946	1	39,18033
TCGA-EE-A2A6-06A	TCGA-EE-A2A6	17,0660792	High	Metastasis	0,455218859	0	85,90164
TCGA-EE-A2GB-06A	TCGA-EE-A2GB	15,8517992	Low	Metastasis	0,387138783	0	59,11475
TCGA-EE-A2GC-06A	TCGA-EE-A2GC	17,183432	High	Metastasis	0,54991074	0	67,2459
TCGA-EE-A2GD-06A	TCGA-EE-A2GD	19,1771552	High	Metastasis	0,772929096	1	339,2131
TCGA-EE-A2GE-06A	TCGA-EE-A2GE	19,207653	High	Metastasis	0,745698278	0	173,3115
TCGA-EE-A2GH-06A	TCGA-EE-A2GH	20,1808121	High	Metastasis	0,676179627	0	219,6393
TCGA-EE-A2GI-06A	TCGA-EE-A2GI	17,9982363	High	Metastasis	0,469882082	0	48,59016
TCGA-EE-A2GJ-06A	TCGA-EE-A2GJ	15,0559763	Low	Metastasis	0,485046439	1	107,082
TCGA-EE-A2GK-06A	TCGA-EE-A2GK	18,7200207	High	Metastasis	0,51780708	0	54,59016
TCGA-EE-A2GL-06A	TCGA-EE-A2GL	16,6997641	Low	Metastasis	0,476099556	0	79,44262
TCGA-EE-A2GM-06B	TCGA-EE-A2GM	17,0189111	High	Metastasis	0,374528918	0	75,27869
TCGA-EE-A2GN-06A	TCGA-EE-A2GN	17,3745161	High	Metastasis	0,530463335	1	101,8361
TCGA-EE-A2GO-06A	TCGA-EE-A2GO	22,1805453	High	Metastasis	0,628810162	0	126,459
TCGA-EE-A2GP-06A	TCGA-EE-A2GP	18,0325106	High	Metastasis	0,637958903	1	13,86885
TCGA-EE-A2GR-06A	TCGA-EE-A2GR	15,3095318	Low	Metastasis	0,408657239	1	42,65574
TCGA-EE-A2GS-06A	TCGA-EE-A2GS	20,061065	High	Metastasis	0,525131173	1	80,98361
TCGA-EE-A2GT-06A	TCGA-EE-A2GT	15,8579155	Low	Metastasis	0,438849631	0	44,7541
TCGA-EE-A2GU-06A	TCGA-EE-A2GU	13,3900809	Low	Metastasis	0,267893255	0	94,55738
TCGA-EE-A2M5-06A	TCGA-EE-A2M5	19,2808638	High	Metastasis	0,638253959	1	21,60656
TCGA-EE-A2M6-06A	TCGA-EE-A2M6	18,3963485	High	Metastasis	0,455151197	0	128,918
TCGA-EE-A2M7-06A	TCGA-EE-A2M7	16,8129928	High	Metastasis	0,537622079	1	28,7541
TCGA-EE-A2M8-06A	TCGA-EE-A2M8	18,2349099	High	Metastasis	0,566270381	1	19,70492
TCGA-EE-A2MC-06A	TCGA-EE-A2MC	18,5072512	High	Metastasis	0,727400261	1	61,34426
TCGA-EE-A2MD-06A	TCGA-EE-A2MD	15,2097467	Low	Metastasis	0,404503177	1	47,14754
TCGA-EE-A2ME-06A	TCGA-EE-A2ME	17,8268157	High	Metastasis	0,659368545	1	102,9836
TCGA-EE-A2MF-06A	TCGA-EE-A2MF	17,0724721	High	Metastasis	0,317862816	1	268
TCGA-EE-A2MG-06A	TCGA-EE-A2MG	18,0407027	High	Metastasis	0,679376027	1	102,918
TCGA-EE-A2MH-06A	TCGA-EE-A2MH	17,9337446	High	Metastasis	0,44428217	1	16,91803
TCGA-EE-A2MI-06A	TCGA-EE-A2MI	17,2387906	High	Metastasis	0,647011475	1	204,0984
TCGA-EE-A2MJ-06A	TCGA-EE-A2MJ	16,4842271	Low	Metastasis	0,466583067	1	95,96721
TCGA-EE-A2MK-06A	TCGA-EE-A2MK	19,546986	High	Metastasis	0,516947749	0	179,9016
TCGA-EE-A2ML-06A	TCGA-EE-A2ML	14,7017222	Low	Metastasis	0,423386357	1	216,0656
TCGA-EE-A2MM-06A	TCGA-EE-A2MM	16,5794742	Low	Metastasis	0,431710183	1	167,4426
TCGA-EE-A2MN-06A	TCGA-EE-A2MN	14,2108178	Low	Metastasis	0,391409281	1	47,40984
TCGA-EE-A2MP-06A	TCGA-EE-A2MP	16,181242	Low	Metastasis	0,455396529	0	247,9672
TCGA-EE-A2MQ-06A	TCGA-EE-A2MQ	17,0974304	High	Metastasis	0,548742599	1	43,11475
TCGA-EE-A2MR-06A	TCGA-EE-A2MR	16,7544479	Low	Metastasis	0,71051129	0	134,0328
TCGA-EE-A2MS-06A	TCGA-EE-A2MS	19,0693616	High	Metastasis	0,475894451	0	162,0328
TCGA-EE-A2MT-06A	TCGA-EE-A2MT	14,6466063	Low	Metastasis	0,47980963	0	71,01639
TCGA-EE-A2MU-06A	TCGA-EE-A2MU	17,1904653	High	Metastasis	0,583238424	0	53,11475
TCGA-EE-A3AA-06A	TCGA-EE-A3AA	17,0705388	High	Metastasis	0,496498223	0	123,9672
TCGA-EE-A3AB-06A	TCGA-EE-A3AB	20,2301569	High	Metastasis	0,649653862	0	122,3934

TCGA-EE-A3AC-06A	TCGA-EE-A3AC	17,9594729	High	Metastasis	0,391866167	0	63,86885
TCGA-EE-A3AD-06A	TCGA-EE-A3AD	17,6001121	High	Metastasis	0,414370719	1	28,68852
TCGA-EE-A3AF-06A	TCGA-EE-A3AF	15,4067806	Low	Metastasis	0,509260936	1	13,77049
TCGA-EE-A3AG-06A	TCGA-EE-A3AG	18,7952886	High	Metastasis	0,596672926	1	41,47541
TCGA-EE-A3AH-06A	TCGA-EE-A3AH	17,1923191	High	Metastasis	0,51501196	1	138,4262
TCGA-EE-A3J3-06A	TCGA-EE-A3J3	18,1653465	High	Metastasis	0,601514925	1	171,7049
TCGA-EE-A3J4-06A	TCGA-EE-A3J4	15,1952865	Low	Metastasis	0,428631177	1	126,8525
TCGA-EE-A3J5-06A	TCGA-EE-A3J5	17,7240354	High	Metastasis	0,543679996	1	36,85246
TCGA-EE-A3J7-06A	TCGA-EE-A3J7	15,0840424	Low	Metastasis	0,400064755	0	63,90164
TCGA-EE-A3J8-06A	TCGA-EE-A3J8	18,0114956	High	Metastasis	0,435394951	1	34,22951
TCGA-EE-A3JA-06A	TCGA-EE-A3JA	16,1405434	Low	Metastasis	0,471449696	1	53,04918
TCGA-EE-A3JB-06A	TCGA-EE-A3JB	19,7269552	High	Metastasis	0,702036426	0	201,2459
TCGA-EE-A3JD-06A	TCGA-EE-A3JD	17,2993996	High	Metastasis	0,578504679	1	27,27869
TCGA-EE-A3JE-06A	TCGA-EE-A3JE	19,1932872	High	Metastasis	0,714720096	0	51,21311
TCGA-EE-A3JH-06A	TCGA-EE-A3JH	17,56605	High	Metastasis	0,580882039	0	133,9672
TCGA-EE-A3JI-06A	TCGA-EE-A3JI	13,5381217	Low	Metastasis	0,264403787	1	152,3934
TCGA-ER-A193-06A	TCGA-ER-A193	19,1170788	High	Metastasis	0,628042939	1	31,31148
TCGA-ER-A194-01A	TCGA-ER-A194	15,9072955	Low	Primary Tumor	0,472047963	1	44,39344
TCGA-ER-A195-06A	TCGA-ER-A195	20,3460384	High	Metastasis	0,701545355	1	35,34426
TCGA-ER-A196-01A	TCGA-ER-A196	15,92601	Low	Primary Tumor	0,656211031	0	58,52459
TCGA-ER-A197-06A	TCGA-ER-A197	16,3789943	Low	Metastasis	0,590357029	1	13,90164
TCGA-ER-A198-06A	TCGA-ER-A198	17,3150576	High	Metastasis	0,380711368	1	50,62295
TCGA-ER-A199-06A	TCGA-ER-A199	16,3730994	Low	Metastasis	0,446271116	1	9,147541
TCGA-ER-A19A-06A	TCGA-ER-A19A	19,4877253	High	Metastasis	0,781081123	0	77,54098
TCGA-ER-A19B-06A	TCGA-ER-A19B	17,7826937	High	Metastasis	0,574771296	1	98,13115
TCGA-ER-A19C-06A	TCGA-ER-A19C	16,7952694	Low	Metastasis	0,57840406	1	48,7541
TCGA-ER-A19D-06A	TCGA-ER-A19D	17,953903	High	Metastasis	0,71529888	1	12,55738
TCGA-ER-A19E-06A	TCGA-ER-A19E	16,6074296	Low	Metastasis	0,588755778	1	12,98361
TCGA-ER-A19F-06A	TCGA-ER-A19F	16,6447498	Low	Metastasis	0,466018554	1	26,29508
TCGA-ER-A19G-06A	TCGA-ER-A19G	15,304236	Low	Metastasis	0,461887671	0	301,2459
TCGA-ER-A19H-06A	TCGA-ER-A19H	18,6751415	High	Metastasis	0,555814047	1	151,9344
TCGA-ER-A19J-06A	TCGA-ER-A19J	17,3879886	High	Metastasis	0,600207061	1	6,42623
TCGA-ER-A19K-01A	TCGA-ER-A19K	16,3513756	Low	Primary Tumor	0,449829144	1	15,37705
TCGA-ER-A19L-06A	TCGA-ER-A19L	19,1500293	High	Metastasis	0,603319044	1	131,1475
TCGA-ER-A19M-06A	TCGA-ER-A19M	18,1595144	High	Metastasis	0,637971563	1	60,88525
TCGA-ER-A19N-06A	TCGA-ER-A19N	18,2484583	High	Metastasis	0,646021181	1	43,96721
TCGA-ER-A19O-06A	TCGA-ER-A19O	18,0028223	High	Metastasis	0,640323538	1	NA
TCGA-ER-A19P-06A	TCGA-ER-A19P	17,7806056	High	Metastasis	0,523234911	1	161,6393
TCGA-ER-A19Q-06A	TCGA-ER-A19Q	17,041144	High	Metastasis	0,59068168	1	50,7541
TCGA-ER-A19S-06A	TCGA-ER-A19S	16,4415601	Low	Metastasis	0,433659954	0	49,34426
TCGA-ER-A19T-01A	TCGA-ER-A19T	16,6447364	Low	Primary Tumor	0,525627351	1	8,852459
TCGA-ER-A19T-06A	TCGA-ER-A19T	15,8451043	Low	Metastasis	0,495979488	1	8,852459
TCGA-ER-A19W-06A	TCGA-ER-A19W	16,6383956	Low	Metastasis	0,483291875	1	147,7705
TCGA-ER-A1A1-06A	TCGA-ER-A1A1	17,3774032	High	Metastasis	0,47711394	0	104,7869
TCGA-ER-A2NB-01A	TCGA-ER-A2NB	15,1868444	Low	Primary Tumor	0,538590809	1	28,09836
TCGA-ER-A2NC-06A	TCGA-ER-A2NC	16,7692853	Low	Metastasis	0,724989839	1	43,70492
TCGA-ER-A2ND-06A	TCGA-ER-A2ND	14,2656114	Low	Metastasis	0,440098348	1	23,27869
TCGA-ER-A2NE-06A	TCGA-ER-A2NE	15,3891859	Low	Metastasis	0,374332404	1	20,09836
TCGA-ER-A2NF-01A	TCGA-ER-A2NF	16,9751138	High	Primary Tumor	0,530546968	1	28,7541
TCGA-ER-A2NF-06A	TCGA-ER-A2NF	13,2177032	Low	Metastasis	0,240200429	1	28,7541
TCGA-ER-A2NG-06A	TCGA-ER-A2NG	16,2381991	Low	Metastasis	0,528761153	1	48,85246
TCGA-ER-A2NH-06A	TCGA-ER-A2NH	17,5386581	High	Metastasis	0,656846716	0	41,44262
TCGA-ER-A3ES-06A	TCGA-ER-A3ES	12,4335096	Low	Metastasis	0,203797165	1	246,3607
TCGA-ER-A3ET-06A	TCGA-ER-A3ET	17,6760042	High	Metastasis	0,630738135	1	92,7541
TCGA-ER-A3EV-06A	TCGA-ER-A3EV	15,9150823	Low	Metastasis	0,457211944	1	46,85246
TCGA-ER-A3PL-06A	TCGA-ER-A3PL	17,5111725	High	Metastasis	0,602626958	0	33,11475
TCGA-ER-A42H-01A	TCGA-ER-A42H	14,6062625	Low	Primary Tumor	0,507634618	1	13,96721
TCGA-ER-A42K-06A	TCGA-ER-A42K	18,1367817	High	Metastasis	0,613701937	1	12,91803
TCGA-ER-A42L-06A	TCGA-ER-A42L	15,9923839	Low	Metastasis	0,578481422	0	148,623
TCGA-FR-A2OS-01A	TCGA-FR-A2OS	16,5868104	Low	Primary Tumor	0,556908317	1	12,06557
TCGA-FR-A3R1-01A	TCGA-FR-A3R1	15,5668107	Low	Primary Tumor	0,420283256	0	22,45902
TCGA-FR-A3YN-06A	TCGA-FR-A3YN	14,9154147	Low	Metastasis	0,378350552	0	92,72131
TCGA-FR-A3YO-06A	TCGA-FR-A3YO	13,6915859	Low	Metastasis	0,572976587	0	NA
TCGA-FR-A44A-06A	TCGA-FR-A44A	16,4622929	Low	Metastasis	0,518267804	0	173,7377
TCGA-FR-A69P-06A	TCGA-FR-A69P	19,1633815	High	Metastasis	0,581536986	0	15,67213
TCGA-FR-A726-01A	TCGA-FR-A726	16,7558496	Low	Primary Tumor	0,55569181	1	10
TCGA-FR-A728-01A	TCGA-FR-A728	16,016755	Low	Primary Tumor	0,573269564	0	19,11475
TCGA-FR-A729-06A	TCGA-FR-A729	19,1067292	High	Metastasis	0,478406344	0	220,1967
TCGA-FR-A7U8-06A	TCGA-FR-A7U8	15,0677114	Low	Metastasis	0,428134177	0	27,77049
TCGA-FR-A7U9-06A	TCGA-FR-A7U9	18,1233886	High	Metastasis	0,712358636	0	18,72131

TCGA-FR-A7UA-06A	TCGA-FR-A7UA	17,0021872	High	Metastasis	0,549446687	0	38,16393
TCGA-FR-A8YC-06A	TCGA-FR-A8YC	17,5391535	High	Metastasis	0,346467852	1	34,72131
TCGA-FR-A8YD-06A	TCGA-FR-A8YD	13,0683739	Low	Metastasis	0,388775859	1	36,16393
TCGA-FR-A8YE-06A	TCGA-FR-A8YE	18,7164408	High	Metastasis	0,62276807	0	104,1311
TCGA-FS-A1YW-06A	TCGA-FS-A1YW	17,9709515	High	Metastasis	0,363937026	1	216,3279
TCGA-FS-A1YX-06A	TCGA-FS-A1YX	18,531566	High	Metastasis	0,591643487	1	48,45902
TCGA-FS-A1YY-06A	TCGA-FS-A1YY	18,6263854	High	Metastasis	0,612095846	1	227,9672
TCGA-FS-A1Z0-06A	TCGA-FS-A1Z0	16,7992555	High	Metastasis	0,448839213	1	202,0984
TCGA-FS-A1Z3-06A	TCGA-FS-A1Z3	16,2673113	Low	Metastasis	0,476472119	1	20,85246
TCGA-FS-A1Z4-06A	TCGA-FS-A1Z4	18,1011124	High	Metastasis	0,550738222	1	28
TCGA-FS-A1Z7-06A	TCGA-FS-A1Z7	18,6239937	High	Metastasis	0,715456615	1	7,770492
TCGA-FS-A1ZA-06A	TCGA-FS-A1ZA	15,9951634	Low	Metastasis	0,58509212	1	27,63934
TCGA-FS-A1ZB-06A	TCGA-FS-A1ZB	18,9223192	High	Metastasis	0,653233846	1	48,72131
TCGA-FS-A1ZC-06A	TCGA-FS-A1ZC	19,5232435	High	Metastasis	0,583646136	1	356,3934
TCGA-FS-A1ZD-06A	TCGA-FS-A1ZD	18,1809582	High	Metastasis	0,696041843	1	53,37705
TCGA-FS-A1ZE-06A	TCGA-FS-A1ZE	16,2299326	Low	Metastasis	0,478558556	1	46,32787
TCGA-FS-A1ZF-06A	TCGA-FS-A1ZF	18,2300716	High	Metastasis	0,549596711	1	15,40984
TCGA-FS-A1ZG-06A	TCGA-FS-A1ZG	14,3167847	Low	Metastasis	0,291938101	1	9,672131
TCGA-FS-A1ZH-06A	TCGA-FS-A1ZH	18,9144152	High	Metastasis	0,75208358	1	32,65574
TCGA-FS-A1ZJ-06A	TCGA-FS-A1ZJ	14,705383	Low	Metastasis	0,395054181	1	47,2459
TCGA-FS-A1ZK-06A	TCGA-FS-A1ZK	15,1477248	Low	Metastasis	0,422568669	1	23,86885
TCGA-FS-A1ZM-06A	TCGA-FS-A1ZM	18,6321044	High	Metastasis	0,670036043	0	100,9836
TCGA-FS-A1ZN-01A	TCGA-FS-A1ZN	18,7196833	High	Primary Tumor	0,474036455	1	23,93443
TCGA-FS-A1ZP-06A	TCGA-FS-A1ZP	16,5167016	Low	Metastasis	0,507350166	1	74,52459
TCGA-FS-A1ZQ-06A	TCGA-FS-A1ZQ	14,5132454	Low	Metastasis	0,510665048	1	133,1803
TCGA-FS-A1ZR-06A	TCGA-FS-A1ZR	18,1252536	High	Metastasis	0,542178406	1	11,37705
TCGA-FS-A1ZS-06A	TCGA-FS-A1ZS	21,5499163	High	Metastasis	0,783666939	0	148,3934
TCGA-FS-A1ZT-06A	TCGA-FS-A1ZT	15,4315161	Low	Metastasis	0,446211013	0	53,01639
TCGA-FS-A1ZU-06A	TCGA-FS-A1ZU	15,1867913	Low	Metastasis	0,388529148	1	26,4918
TCGA-FS-A1ZW-06A	TCGA-FS-A1ZW	17,5211329	High	Metastasis	0,452863143	0	49,34426
TCGA-FS-A1ZY-06A	TCGA-FS-A1ZY	18,2851662	High	Metastasis	0,397929801	1	27,01639
TCGA-FS-A1ZZ-06A	TCGA-FS-A1ZZ	15,6183973	Low	Metastasis	0,351947537	1	26,95082
TCGA-FS-A4F0-06A	TCGA-FS-A4F0	13,7533291	Low	Metastasis	0,229922616	0	77,60656
TCGA-FS-A4F2-06A	TCGA-FS-A4F2	15,1518021	Low	Metastasis	0,47093605	1	50
TCGA-FS-A4F4-06A	TCGA-FS-A4F4	17,0326146	High	Metastasis	0,596273954	1	66,4918
TCGA-FS-A4F5-06A	TCGA-FS-A4F5	16,637603	Low	Metastasis	0,510412684	1	28,65574
TCGA-FS-A4F8-06A	TCGA-FS-A4F8	18,7972189	High	Metastasis	0,630594447	1	174,3607
TCGA-FS-A4F9-06A	TCGA-FS-A4F9	14,4447682	Low	Metastasis	0,377485219	0	33,93443
TCGA-FS-A4FB-06A	TCGA-FS-A4FB	18,2158929	High	Metastasis	0,524324376	1	26,65574
TCGA-FS-A4FC-06A	TCGA-FS-A4FC	16,3001229	Low	Metastasis	0,58424765	1	54,2623
TCGA-FS-A4FD-06A	TCGA-FS-A4FD	16,2044796	Low	Metastasis	0,470828734	1	80,45902
TCGA-FW-A3I3-06A	TCGA-FW-A3I3	16,5559472	Low	Metastasis	0,417430169	0	17,40984
TCGA-FW-A3R5-06A	TCGA-FW-A3R5	16,9273276	High	Metastasis	0,560965265	0	36,85246
TCGA-FW-A3TU-06A	TCGA-FW-A3TU	11,4280021	Low	Metastasis	0,344677485	1	55,44262
TCGA-FW-A3TV-06A	TCGA-FW-A3TV	15,9841905	Low	Metastasis	0,483570938	0	13,47541
TCGA-FW-A5DX-01A	TCGA-FW-A5DX	15,9985412	Low	Primary Tumor	0,427145838	0	20,98361
TCGA-FW-A5DY-06A	TCGA-FW-A5DY	16,9348787	High	Metastasis	0,492037939	0	19,2459
TCGA-GF-A2C7-01A	TCGA-GF-A2C7	16,4232319	Low	Primary Tumor	0,50113678	0	0,688525
TCGA-GF-A3OT-06A	TCGA-GF-A3OT	19,3250636	High	Metastasis	0,837966465	0	9,868852
TCGA-GF-A4EO-06A	TCGA-GF-A4EO	16,1250398	Low	Metastasis	0,484787042	0	19,37705
TCGA-GF-A6C8-06A	TCGA-GF-A6C8	17,6395972	High	Metastasis	0,467276659	0	2,032787
TCGA-GF-A6C9-06A	TCGA-GF-A6C9	17,3616456	High	Metastasis	0,653357711	0	15,7377
TCGA-GF-A769-01A	TCGA-GF-A769	17,6767021	High	Primary Tumor	0,50197959	1	35,08197
TCGA-GN-A262-06A	TCGA-GN-A262	18,1769059	High	Metastasis	0,319054202	0	139,5082
TCGA-GN-A263-01A	TCGA-GN-A263	18,1743389	High	Primary Tumor	0,581715302	1	15,31148
TCGA-GN-A264-06A	TCGA-GN-A264	16,6365319	Low	Metastasis	0,586222123	1	117,6066
TCGA-GN-A265-06A	TCGA-GN-A265	18,6478643	High	Metastasis	0,789811058	0	96,65574
TCGA-GN-A266-06A	TCGA-GN-A266	19,9621905	High	Metastasis	0,783780136	1	10,09836
TCGA-GN-A267-06A	TCGA-GN-A267	16,4284588	Low	Metastasis	0,445139502	1	64,2623
TCGA-GN-A268-06A	TCGA-GN-A268	14,5455776	Low	Metastasis	0,336254205	1	62,62295
TCGA-GN-A26A-06A	TCGA-GN-A26A	20,4227072	High	Metastasis	0,726920915	1	32,39344
TCGA-GN-A26C-01A	TCGA-GN-A26C	17,0091303	High	Primary Tumor	0,685748579	1	26,91803
TCGA-GN-A26D-06A	TCGA-GN-A26D	14,4058319	Low	Metastasis	0,44974353	1	47,86885
TCGA-GN-A4U3-06A	TCGA-GN-A4U3	14,854681	Low	Metastasis	0,34839336	0	121,5738
TCGA-GN-A4U4-06A	TCGA-GN-A4U4	14,882976	Low	Metastasis	0,308401438	0	39,2459
TCGA-GN-A4U5-01A	TCGA-GN-A4U5	18,3976132	High	Primary Tumor	0,510138054	0	37,90164
TCGA-GN-A4U7-06A	TCGA-GN-A4U7	13,9638474	Low	Metastasis	0,375301353	1	10,39344
TCGA-GN-A4U8-06A	TCGA-GN-A4U8	18,1689144	High	Metastasis	0,403687107	0	48,7541
TCGA-GN-A4U9-06A	TCGA-GN-A4U9	17,6758796	High	Metastasis	0,506647192	1	22,06557
TCGA-GN-A8LK-06A	TCGA-GN-A8LK	13,2068273	Low	Metastasis	0,296384934	1	49,96721

TCGA-GN-A8LL-06A	TCGA-GN-A8LL	13,0544307	Low	Metastasis	0,352874439	1	21,31148
TCGA-GN-A8LN-01A	TCGA-GN-A8LN	19,23281	High	Primary Tumor	0,540264699	0	25,31148
TCGA-GN-A9SD-06A	TCGA-GN-A9SD	17,8850373	High	Metastasis	0,592239352	1	59,2459
TCGA-HR-A2OG-06A	TCGA-HR-A2OG	19,4432932	High	Metastasis	0,799270749	0	0,229508
TCGA-HR-A2OH-06A	TCGA-HR-A2OH	15,7024455	Low	Metastasis	0,465153358	1	65,70492
TCGA-HR-A5NC-01A	TCGA-HR-A5NC	17,9435993	High	Primary Tumor	0,538964543	0	32,78689
TCGA-IH-A3EA-01A	TCGA-IH-A3EA	14,0038567	Low	Primary Tumor	0,444842936	0	17,18033
TCGA-LH-A9QB-06A	TCGA-LH-A9QB	17,4041785	High	Metastasis	0,531573639	0	367,7705
TCGA-OD-A75X-06A	TCGA-OD-A75X	16,200485	Low	Metastasis	0,434715767	1	297,082
TCGA-QB-A6FS-06A	TCGA-QB-A6FS	14,8086295	Low	Metastasis	0,513177183	0	7,213115
TCGA-QB-AA9O-06A	TCGA-QB-AA9O	17,1538724	High	Metastasis	0,414451515	1	18
TCGA-RP-A690-06A	TCGA-RP-A690	12,9701683	Low	Metastasis	0,312438406	0	0,196721
TCGA-RP-A693-06A	TCGA-RP-A693	15,2705436	Low	Metastasis	0,513265897	0	0,327869
TCGA-RP-A694-06A	TCGA-RP-A694	16,1184552	Low	Metastasis	0,432978857	0	0,688525
TCGA-RP-A695-06A	TCGA-RP-A695	13,6837156	Low	Metastasis	0,37817953	0	NA
TCGA-RP-A6K9-06A	TCGA-RP-A6K9	16,3386555	Low	Metastasis	0,469426393	0	NA
TCGA-W3-A824-06A	TCGA-W3-A824	16,6975774	Low	Metastasis	0,389539353	0	227,541
TCGA-W3-A825-06A	TCGA-W3-A825	16,0678277	Low	Metastasis	0,721332311	1	62,85246
TCGA-W3-A828-06A	TCGA-W3-A828	21,1572105	High	Metastasis	0,863209886	1	120,7541
TCGA-W3-AA1O-06A	TCGA-W3-AA1O	16,0357772	Low	Metastasis	0,457363617	1	4
TCGA-W3-AA1Q-06A	TCGA-W3-AA1Q	15,2985583	Low	Metastasis	0,40397113	1	68,88525
TCGA-W3-AA1R-06A	TCGA-W3-AA1R	14,520857	Low	Metastasis	0,342097854	1	110,7869
TCGA-W3-AA1V-06B	TCGA-W3-AA1V	15,7998473	Low	Metastasis	0,599941616	1	41,96721
TCGA-W3-AA1W-06A	TCGA-W3-AA1W	15,0768793	Low	Metastasis	0,386946297	0	218,5574
TCGA-W3-AA21-06A	TCGA-W3-AA21	15,8970577	Low	Metastasis	0,366438352	1	104,7541
TCGA-WE-A8JZ-06A	TCGA-WE-A8JZ	16,493866	Low	Metastasis	0,468052119	0	23,96721
TCGA-WE-A8K1-06A	TCGA-WE-A8K1	15,7979181	Low	Metastasis	0,441636094	0	48,91803
TCGA-WE-A8K4-01A	TCGA-WE-A8K4	14,0122318	Low	Primary Tumor	0,359896543	0	20,13115
TCGA-WE-A8K5-06A	TCGA-WE-A8K5	18,0379879	High	Metastasis	0,559114367	1	60,98361
TCGA-WE-A8K6-06A	TCGA-WE-A8K6	20,4876601	High	Metastasis	0,550499367	0	17,90164
TCGA-WE-A8ZM-06A	TCGA-WE-A8ZM	16,4892698	Low	Metastasis	0,59006225	0	101,0492
TCGA-WE-A8ZN-06A	TCGA-WE-A8ZN	17,7214196	High	Metastasis	0,565434884	0	58,81967
TCGA-WE-A8ZO-06A	TCGA-WE-A8ZO	16,9371973	High	Metastasis	0,408239789	0	70,32787
TCGA-WE-A8ZQ-06A	TCGA-WE-A8ZQ	17,1812071	High	Metastasis	0,539819677	0	63,04918
TCGA-WE-A8ZR-06A	TCGA-WE-A8ZR	15,4735054	Low	Metastasis	0,444137042	1	8,983607
TCGA-WE-A8ZT-06A	TCGA-WE-A8ZT	16,9903957	High	Metastasis	0,360113046	0	11,77049
TCGA-WE-A8ZX-06A	TCGA-WE-A8ZX	14,686295	Low	Metastasis	0,445039184	0	35,70492
TCGA-WE-A8ZY-06A	TCGA-WE-A8ZY	18,2145482	High	Metastasis	0,458587202	1	49,37705
TCGA-WE-AA9Y-06A	TCGA-WE-AA9Y	17,8529771	High	Metastasis	0,741556113	0	12,13115
TCGA-WE-AAA0-06A	TCGA-WE-AAA0	15,3211079	Low	Metastasis	0,41839055	0	40,29508
TCGA-WE-AAA3-06A	TCGA-WE-AAA3	17,4013298	High	Metastasis	0,56478104	0	21,34426
TCGA-WE-AAA4-06A	TCGA-WE-AAA4	15,5715254	Low	Metastasis	0,557941061	0	24,91803
TCGA-XV-A9VZ-01A	TCGA-XV-A9VZ	16,7527391	Low	Primary Tumor	0,582581769	0	0,360656
TCGA-XV-A9W2-01A	TCGA-XV-A9W2	15,4473027	Low	Primary Tumor	0,433945403	0	13,67213
TCGA-XV-A9W5-01A	TCGA-XV-A9W5	17,4254087	High	Primary Tumor	0,646445434	0	12,85246
TCGA-XV-AAZV-01A	TCGA-XV-AAZV	17,7878716	High	Primary Tumor	0,60857598	0	13,5082
TCGA-XV-AAZW-01A	TCGA-XV-AAZW	18,8620821	High	Primary Tumor	0,572514451	1	12,88525
TCGA-XV-AAZY-01A	TCGA-XV-AAZY	15,5200819	Low	Primary Tumor	0,46738645	0	13,27869
TCGA-XV-AB01-06A	TCGA-XV-AB01	14,9979758	Low	Metastasis	0,538053836	0	13,21311
TCGA-YD-A89C-06A	TCGA-YD-A89C	14,7452589	Low	Metastasis	0,460750197	0	6,885246
TCGA-YD-A9TA-06A	TCGA-YD-A9TA	16,8428681	High	Metastasis	0,674299583	0	49,04918
TCGA-YD-A9TB-06A	TCGA-YD-A9TB	17,0904276	High	Metastasis	0,607940194	0	NA
TCGA-YG-AA3N-01A	TCGA-YG-AA3N	13,8856109	Low	Primary Tumor	0,40572464	0	10,03279
TCGA-YG-AA3O-06A	TCGA-YG-AA3O	16,0705086	Low	Metastasis	0,469634355	1	37,83607
TCGA-YG-AA3P-06A	TCGA-YG-AA3P	13,742929	Low	Metastasis	0,257476626	0	14,39344
TCGA-Z2-A8RT-06A	TCGA-Z2-A8RT	15,6964127	Low	Metastasis	0,430050716	0	27,5082
TCGA-Z2-AA3S-06A	TCGA-Z2-AA3S	14,4773162	Low	Metastasis	0,233692368	0	96,72131
TCGA-Z2-AA3V-06A	TCGA-Z2-AA3V	14,3587498	Low	Metastasis	0,457157562	0	15,93443
