# Supplementary material for: PRRX1 silencing is required for metastatic outgrowth in melanoma and is an independent prognostic of reduced survival in patients
Source: Mol Oncol. 2024 Jul 8;18(10):2471–94. doi: 10.1002/1878-0261.13688 (PMC11459042; doi:10.1002/1878-0261.13688)
Supplement: Supplementary file 6 — Table S8. Hallmarks Gene sets from pre‐ranked GSEA in TCGA‐SKCM (invasive vs non invasive). [file MOL2-18-2471-s004.pdf]

SUPPLEMENTARY TABLE 8

RESULTS RELATED TO FIGURE 1F-1G

Gene sets hallmarks .all.v2023.2.Hs.symbols.gmt

GEO **PRE-RANKED GSEA TCGA-SKCM**

phenotype **(INVASIVE vs NON INVASIVE GENES)**

ranked by Invasiveness_score_n=200 genes

Table: Gene sets enriched in phenotype (**POSITIVELY** ENRICHED)

	GS follow link to MSigDB	GS DETAILS	SIZE	ES	NES	NOM p-val	FDR q-val	FWER p-val	RANK AT MAX	LEADING EDGE
1	HALLMARK_EPITHELIAL_MESENCHYMAL_TRANSITION	Details ...	200	0.80	3.03	0.000	0.000	0.000	2444	tags=77%, list=13%, signal=87%
2	HALLMARK_INFLAMMATORY_RESPONSE	Details ...	199	0.66	2.48	0.000	0.000	0.000	3244	tags=59%, list=17%, signal=70%
3	HALLMARK_KRAS_SIGNALING_UP	Details ...	200	0.65	2.47	0.000	0.000	0.000	3733	tags=58%, list=20%, signal=71%
4	HALLMARK_ANGIOGENESIS	Details ...	36	0.80	2.41	0.000	0.000	0.000	2768	tags=72%, list=14%, signal=84%
5	HALLMARK_TNFA_SIGNALING_VIA_NFKB	Details ...	199	0.61	2.31	0.000	0.000	0.000	4591	tags=61%, list=24%, signal=79%
6	HALLMARK_IL6_JAK_STAT3_SIGNALING	Details ...	87	0.65	2.29	0.000	0.000	0.000	3762	tags=63%, list=20%, signal=78%
7	HALLMARK_APICAL_JUNCTION	Details ...	199	0.58	2.22	0.000	0.000	0.000	3140	tags=46%, list=16%, signal=54%
8	HALLMARK_COAGULATION	Details ...	138	0.60	2.21	0.000	0.000	0.000	4155	tags=50%, list=22%, signal=63%
9	HALLMARK_TGF_BETA_SIGNALING	Details ...	54	0.64	2.10	0.000	0.000	0.000	3138	tags=46%, list=16%, signal=55%
10	HALLMARK_MYOGENESIS	Details ...	199	0.55	2.09	0.000	0.000	0.000	4593	tags=54%, list=24%, signal=71%
11	HALLMARK_COMPLEMENT	Details ...	200	0.54	2.06	0.000	0.000	0.000	4734	tags=51%, list=25%, signal=66%
12	HALLMARK_UV_RESPONSE_DN	Details ...	144	0.55	2.03	0.000	0.000	0.000	2619	tags=37%, list=14%, signal=42%
13	HALLMARK_IL2_STAT5_SIGNALING	Details ...	198	0.53	2.03	0.000	0.000	0.000	3754	tags=47%, list=20%, signal=58%
14	HALLMARK_ALLOGRAFT_REJECTION	Details ...	200	0.52	1.98	0.000	0.000	0.000	5298	tags=58%, list=28%, signal=79%
15	HALLMARK_HYPOXIA	Details ...	199	0.51	1.96	0.000	0.000	0.000	3705	tags=44%, list=19%, signal=54%
16	HALLMARK_INTERFERON_GAMMA_RESPONSE	Details ...	198	0.50	1.89	0.000	0.000	0.001	4879	tags=49%, list=26%, signal=65%
17	HALLMARK_APOPTOSIS	Details ...	160	0.47	1.77	0.000	0.001	0.013	4742	tags=44%, list=25%, signal=59%
18	HALLMARK_NOTCH_SIGNALING	Details ...	32	0.56	1.70	0.005	0.001	0.028	1605	tags=34%, list=8%, signal=37%
19	HALLMARK_ESTROGEN_RESPONSE_LATE	Details ...	199	0.43	1.64	0.000	0.002	0.058	4744	tags=42%, list=25%, signal=56%
20	HALLMARK_KRAS_SIGNALING_DN	Details ...	199	0.42	1.62	0.000	0.003	0.079	5025	tags=43%, list=26%, signal=58%
21	HALLMARK_APICAL_SURFACE		44	0.50	1.60	0.005	0.003	0.093	3368	tags=41%, list=18%, signal=50%
22	HALLMARK_ESTROGEN_RESPONSE_EARLY		199	0.42	1.60	0.000	0.003	0.094	4378	tags=41%, list=23%, signal=53%
23	HALLMARK_XENOBIOTIC_METABOLISM		200	0.40	1.54	0.001	0.007	0.185	2701	tags=28%, list=14%, signal=32%
24	HALLMARK_P53_PATHWAY		199	0.38	1.43	0.002	0.021	0.485	4349	tags=35%, list=23%, signal=45%

RESULTS RELATED TO FIGURE 1F

Gene sets database hallmarks .all.v2023.2.Hs.symbols.gmt

GEO **PRE-RANKED GSEA TCGA-SKCM**

phenotype **(INVASIVE vs NON INVASIVE GENES)**

ranked by Invasiveness_score_n=200 genes

Table: Gene sets enriched in phenotype **NEGATIVELY**

	GS follow link to MSigDB	GS DETAILS	SIZE	ES	NES	NOM p-val	FDR q-val	FWER p-val	RANK AT MAX	LEADING EDGE
1	HALLMARK_MYC_TARGETS_V1	Details ...	200	-0.63	-3.34	0.000	0.000	0.000	5223	tags=75%, list=27%, signal=101%
2	HALLMARK_OXIDATIVE_PHOSPHORYLATION	Details ...	200	-0.63	-3.32	0.000	0.000	0.000	4309	tags=67%, list=23%, signal=86%
3	HALLMARK_E2F_TARGETS	Details ...	200	-0.60	-3.15	0.000	0.000	0.000	5627	tags=70%, list=29%, signal=98%
4	HALLMARK_MYC_TARGETS_V2	Details ...	58	-0.69	-2.96	0.000	0.000	0.000	5276	tags=83%, list=28%, signal=114%
5	HALLMARK_G2M_CHECKPOINT	Details ...	199	-0.49	-2.55	0.000	0.000	0.000	5536	tags=60%, list=29%, signal=84%
6	HALLMARK_DNA_REPAIR	Details ...	150	-0.43	-2.16	0.000	0.000	0.000	4075	tags=41%, list=21%, signal=51%
7	HALLMARK_UNFOLDED_PROTEIN_RESPONSE	Details ...	112	-0.31	-1.47	0.000	0.033	0.046	3015	tags=29%, list=16%, signal=35%
8	HALLMARK_FATTY_ACID_METABOLISM	Details ...	157	-0.28	-1.42	0.000	0.053	0.077	3778	tags=32%, list=20%, signal=40%
9	HALLMARK_MTORC1_SIGNALING	Details ...	199	-0.24	-1.29	0.000	0.102	0.176	4326	tags=35%, list=23%, signal=45%
10	HALLMARK_PROTEIN_SECRETION	Details ...	96	-0.26	-1.25	0.067	0.114	0.219	3189	tags=25%, list=17%, signal=30%
11	HALLMARK_PEROXISOME	Details ...	103	-0.24	-1.19	0.065	0.170	0.331	4218	tags=32%, list=22%, signal=41%
12	HALLMARK_MITOTIC_SPINDLE	Details ...	199	-0.21	-1.07	0.231	0.356	0.617	4514	tags=35%, list=24%, signal=46%
13	HALLMARK_HEME_METABOLISM	Details ...	197	-0.20	-1.05	0.316	0.396	0.695	2883	tags=21%, list=15%, signal=25%
14	HALLMARK_PI3K_AKT_MTOR_SIGNALING	Details ...	105	-0.19	-0.90	0.731	0.789	0.922	4239	tags=30%, list=22%, signal=39%
15	HALLMARK_SPERMATOGENESIS	Details ...	133	-0.18	-0.87	0.850	0.787	0.938	2918	tags=19%, list=15%, signal=22%
