# Supplementary material for: PRRX1 silencing is required for metastatic outgrowth in melanoma and is an independent prognostic of reduced survival in patients
Source: Mol Oncol. 2024 Jul 8;18(10):2471–94. doi: 10.1002/1878-0261.13688 (PMC11459042; doi:10.1002/1878-0261.13688)
Supplement: Supplementary file 7 — Table S9. EMT genes from pre‐ranked GSEA in TCGA‐SKCM (invasive vs non invasive) and Hallmarks Gene sets from GSEA (PRRX1Pearson correlation) in GSE22155; GSE65904; GSE116237. [file MOL2-18-2471-s006.pdf]

Supplementary Table 9: Gene Set Members on the Rank ordered list

Enrichment plot	HALLMARK_EPITHELIAL MESENCHYMAL TRANSITION

PRE-RANKED GSEA TCGA-SKCM (INVASIVE vs NON INVASIVE GENES)

Enrichment Score (ES)	0.7386205
Normalized Enrichment	3.181222
Nominal p-value	0.0
FDR q-value	0.0
FWER p-Value	0.0

	SYMBOL	ANK IN GENE LI	RANK METRIC SCORE	RUNNING ES	ORE ENRICHME
1	CCN2	1	9.235	0.0137	Yes
2	COL12A1	2	8.422	0.0263	Yes
3	COL1A2	3	8.244	0.0386	Yes
4	COL5A2	4	7.954	0.0504	Yes
5	CDH11	6	7.749	0.0619	Yes
6	VCAN	7	7.679	0.0734	Yes
7	NTM	9	7.309	0.0842	Yes
8	INHBA	12	7.204	0.0949	Yes
9	NID2	13	7.199	0.1056	Yes
10	PDGFRB	19	6.957	0.1157	Yes
11	LOXL2	28	6.834	0.1255	Yes
12	FBN1	31	6.792	0.1355	Yes
13	COL6A3	36	6.676	0.1453	Yes
14	COL6A2	39	6.609	0.1550	Yes
15	TGFBI	41	6.589	0.1648	Yes
16	COL4A1	43	6.562	0.1745	Yes
17	THBS1	46	6.513	0.1841	Yes
18	COL5A1	47	6.483	0.1938	Yes
19	DCN	49	6.460	0.2034	Yes
20	ANPEP	55	6.269	0.2125	Yes
21	MXRA5	60	6.221	0.2215	Yes
22	COL3A1	67	6.140	0.2304	Yes
23	LAMA2	70	6.077	0.2393	Yes
24	SPARC	73	6.054	0.2483	Yes
25	PMEPA1	77	6.002	0.2570	Yes
26	ITGA5	78	5.994	0.2660	Yes
27	COL8A2	87	5.874	0.2743	Yes
28	SLIT3	89	5.865	0.2830	Yes
29	FMOD	93	5.771	0.2915	Yes
30	THY1	95	5.752	0.3000	Yes
31	**PRRX1**	98	5.729	0.3084	Yes
32	FBN2	101	5.723	0.3169	Yes
33	COL1A1	102	5.709	0.3254	Yes
34	ECM2	103	5.700	0.3339	Yes
35	WNT5A	108	5.630	0.3421	Yes
36	FBLN2	116	5.566	0.3500	Yes
37	SERPINE1	132	5.453	0.3573	Yes
38	COL5A3	144	5.326	0.3647	Yes
39	TAGLN	156	5.234	0.3719	Yes
40	THBS2	157	5.227	0.3797	Yes
41	COL4A2	159	5.214	0.3874	Yes
42	LUM	167	5.174	0.3948	Yes
43	GLIPR1	187	5.095	0.4014	Yes
44	NT5E	202	5.038	0.4081	Yes
45	GJA1	227	4.918	0.4142	Yes
46	IGFBP4	229	4.913	0.4215	Yes
47	MYLK	243	4.803	0.4279	Yes
48	NNMT	253	4.764	0.4346	Yes
49	LRP1	272	4.695	0.4406	Yes
50	DPYSL3	280	4.673	0.4472	Yes
51	FOXC2	301	4.607	0.4530	Yes
52	BGN	305	4.593	0.4597	Yes
53	EFEMP2	312	4.575	0.4662	Yes
54	LRRC15	336	4.490	0.4717	Yes
55	EDIL3	371	4.394	0.4764	Yes
56	VEGFC	383	4.367	0.4824	Yes
57	VCAM1	393	4.344	0.4884	Yes
58	CDH6	404	4.316	0.4943	Yes
59	RGS4	437	4.234	0.4989	Yes
60	FAP	443	4.218	0.5049	Yes
61	CADM1	446	4.217	0.5111	Yes
62	HTRA1	464	4.175	0.5164	Yes
63	ADAM12	497	4.095	0.5208	Yes
64	FSTL1	498	4.092	0.5269	Yes
65	TPM1	506	4.071	0.5326	Yes
66	FGF2	537	4.021	0.5370	Yes
67	MFAP5	555	3.992	0.5421	Yes
68	MGP	563	3.983	0.5477	Yes
69	COL7A1	603	3.918	0.5514	Yes
70	ABI3BP	627	3.885	0.5560	Yes
71	POSTN	644	3.856	0.5609	Yes
72	FBLN1	647	3.852	0.5666	Yes
73	GREM1	674	3.801	0.5708	Yes
74	ACTA2	680	3.795	0.5762	Yes
75	GPC1	688	3.786	0.5815	Yes
76	TPM4	726	3.738	0.5851	Yes
77	SFRP1	736	3.731	0.5902	Yes
78	CCN1	781	3.672	0.5933	Yes
79	LOX	798	3.653	0.5979	Yes
80	CXCL12	799	3.651	0.6034	Yes
81	MATN3	801	3.647	0.6088	Yes
82	SLIT2	837	3.595	0.6123	Yes
83	AREG	852	3.581	0.6169	Yes
84	TFPI2	860	3.574	0.6218	Yes
85	BASP1	875	3.553	0.6264	Yes
86	COL11A1	896	3.525	0.6306	Yes
87	GEM	964	3.454	0.6322	Yes
88	SDC1	967	3.447	0.6372	Yes
89	LOXL1	985	3.415	0.6414	Yes
90	MMP2	986	3.414	0.6465	Yes

91	PLAUR	1020	3.370	0.6498	Yes
92	MEST	1021	3.370	0.6548	Yes
93	SERPINH1	1028	3.364	0.6595	Yes
94	MATN2	1034	3.358	0.6642	Yes
95	PCOLCE	1043	3.347	0.6688	Yes
96	APLP1	1127	3.261	0.6693	Yes
97	ITGA2	1130	3.258	0.6740	Yes
98	TNFRSF11B	1139	3.250	0.6784	Yes
99	FN1	1141	3.249	0.6832	Yes
100	IGFBP2	1209	3.188	0.6844	Yes
101	PTX3	1283	3.120	0.6852	Yes
102	PTHLH	1354	3.059	0.6861	Yes
103	GADD45B	1441	2.979	0.6859	Yes
104	MMP3	1468	2.958	0.6890	Yes
105	TNC	1494	2.935	0.6920	Yes
106	ITGB3	1586	2.866	0.6915	Yes
107	LAMA3	1611	2.845	0.6944	Yes
108	TGFB1	1625	2.833	0.6980	Yes
109	IGFBP3	1752	2.752	0.6954	Yes
110	TIMP1	1784	2.730	0.6978	Yes
111	ELN	1788	2.728	0.7017	Yes
112	CRLF1	1798	2.721	0.7053	Yes
113	GPX7	1818	2.703	0.7083	Yes
114	QSOX1	1910	2.653	0.7074	Yes
115	SPOCK1	1925	2.643	0.7106	Yes
116	SNTB1	2009	2.587	0.7101	Yes
117	ITGB5	2070	2.556	0.7107	Yes
118	LAMC1	2100	2.542	0.7130	Yes
119	COMP	2143	2.522	0.7145	Yes
120	IL6	2154	2.517	0.7177	Yes
121	CTHRC1	2200	2.486	0.7190	Yes
122	LAMC2	2216	2.477	0.7219	Yes
123	SCG2	2217	2.475	0.7256	Yes
124	BDNF	2227	2.471	0.7288	Yes
125	PDLIM4	2322	2.423	0.7275	Yes
126	FUCA1	2337	2.417	0.7303	Yes
127	FAS	2343	2.413	0.7337	Yes
128	SGCB	2383	2.396	0.7352	Yes
129	FBLN5	2386	2.395	0.7386	Yes

Gene sets database hallmarks .all.v2023.2.Hs.symbols.gmt

**GEO**	**GSE22155**
**phenotype**	PRRX1
**ranked by**	pearson correlation

POSITIVELY CORRELATED

GS<br>follow link to MSigDB	GS DETAILS	SIZE	ES	NES	NOM p-val	FDR q-val	FWER p-val	RANK AT MAX	LEADING EDGE
HALLMARK_EPITHELIAL_MESENCHYMAL_TRANSITION	Details ...	177	0.69	3.36	0.000	0.000	0.000	1999	tags=53%, list=12%, signal=60%
HALLMARK_UV_RESPONSE_DN	Details ...	128	0.57	2.65	0.000	0.000	0.000	2219	tags=41%, list=13%, signal=46%
HALLMARK_KRAS_SIGNALING_UP	Details ...	165	0.48	2.30	0.000	0.000	0.000	1873	tags=28%, list=11%, signal=31%
HALLMARK_MYOGENESIS	Details ...	173	0.47	2.29	0.000	0.000	0.000	2322	tags=29%, list=14%, signal=34%
HALLMARK_ANGIOGENESIS	Details ...	30	0.65	2.28	0.000	0.000	0.000	2524	tags=47%, list=15%, signal=55%
HALLMARK_TNFA_SIGNALING_VIA_NFKB	Details ...	167	0.46	2.21	0.000	0.000	0.000	4377	tags=47%, list=26%, signal=63%
HALLMARK_APICAL_JUNCTION	Details ...	170	0.45	2.20	0.000	0.000	0.000	3617	tags=39%, list=21%, signal=49%
HALLMARK_COAGULATION	Details ...	123	0.47	2.17	0.000	0.000	0.000	2505	tags=35%, list=15%, signal=41%
HALLMARK_TGF_BETA_SIGNALING	Details ...	46	0.54	2.09	0.000	0.000	0.000	3964	tags=52%, list=23%, signal=68%
HALLMARK_INFLAMMATORY_RESPONSE	Details ...	166	0.39	1.88	0.000	0.002	0.013	4409	tags=39%, list=26%, signal=51%
HALLMARK_INTERFERON_GAMMA_RESPONSE	Details ...	161	0.38	1.87	0.000	0.002	0.016	3923	tags=38%, list=23%, signal=49%
HALLMARK_APICAL_SURFACE	Details ...	38	0.48	1.80	0.004	0.002	0.023	3387	tags=37%, list=20%, signal=46%
HALLMARK_IL6_JAK_STAT3_SIGNALING	Details ...	72	0.42	1.79	0.002	0.002	0.025	4509	tags=51%, list=26%, signal=69%
HALLMARK_COMPLEMENT	Details ...	169	0.38	1.79	0.000	0.003	0.029	3708	tags=32%, list=22%, signal=40%
HALLMARK_ALLOGRAFT_REJECTION	Details ...	176	0.36	1.73	0.000	0.004	0.041	3936	tags=39%, list=23%, signal=50%

NEGATIVELY CORRELATED

GS<br>follow link to MSigDB	GS DETAILS	SIZE	ES	NES	NOM p-val	FDR q-val	FWER p-val	RANK AT MAX	LEADING EDGE
HALLMARK_INTERFERON_ALPHA_RESPONSE		80	0.31	1.37	0.046	0.052	0.579	3923	tags=38%, list=23%, signal=48%
HALLMARK_HEDGEHOG_SIGNALING		30	0.38	1.35	0.072	0.060	0.637	1576	tags=30%, list=9%, signal=33%
HALLMARK_HYPOXIA		170	0.27	1.31	0.042	0.077	0.757	2840	tags=24%, list=17%, signal=29%
HALLMARK_P53_PATHWAY		162	0.26	1.22	0.105	0.152	0.945	3030	tags=25%, list=18%, signal=30%
HALLMARK_BILE_ACID_METABOLISM		91	0.26	1.14	0.206	0.254	0.997	3944	tags=30%, list=23%, signal=38%
HALLMARK_FATTY_ACID_METABOLISM		124	0.24	1.12	0.223	0.278	0.999	4003	tags=30%, list=23%, signal=39%
HALLMARK_ESTROGEN_RESPONSE_EARLY		171	0.23	1.09	0.289	0.334	1.000	2020	tags=15%, list=12%, signal=16%
HALLMARK_MITOTIC_SPINDLE		167	0.23	1.08	0.274	0.329	1.000	2938	tags=20%, list=17%, signal=24%
HALLMARK_KRAS_SIGNALING_DN		163	0.20	0.98	0.506	0.549	1.000	2361	tags=15%, list=14%, signal=18%
HALLMARK_HEME_METABOLISM		173	0.20	0.94	0.623	0.620	1.000	2977	tags=20%, list=17%, signal=24%
HALLMARK_WNT_BETA_CATENIN_SIGNALING		38	0.24	0.89	0.624	0.731	1.000	3470	tags=26%, list=20%, signal=33%
HALLMARK_CHOLESTEROL_HOMEOSTASIS		64	0.19	0.77	0.887	0.927	1.000	3458	tags=25%, list=20%, signal=31%

Enrichment plot: HALLMARK_EPITHELIAL_MESENCHYMAL_TRANSITION
Profile of the Running ES Score & Positions of GeneSet Members on the Rank Ordered List

	SYMBOL	TITLE	RANK IN	RANK MI	RUNNING E	CORE ENRICHMENT
1	PRRX1	paired related	0	1000	0.0202	Yes
2	PDGFRB	platelet derive	3	0.700	0.0342	Yes
3	CDH11	cadherin 11 [S	7	0.669	0.0475	Yes
4	COL6A2	collagen type	8	0.667	0.0610	Yes
5	NID2	nidogen 2 [So	19	0.634	0.0732	Yes
6	MMP2	matrix metallo	33	0.614	0.0848	Yes
7	FBN1	fibrillin 1 [Sour	38	0.602	0.0967	Yes
8	COL1A2	collagen type	43	0.599	0.1086	Yes
9	COL6A3	collagen type	44	0.596	0.1206	Yes
10	ELN	elastin [Source	45	0.595	0.1327	Yes
11	LUM	lumican [Sourc	49	0.592	0.1444	Yes
12	CXCL12	C-X-C motif ch	51	0.591	0.1563	Yes

13	CALD1	caldesmon 1 [	54	0.589	0.1681	Yes
14	COL3A1	collagen type	59	0.584	0.1796	Yes
15	ABI3BP	ABI family mer	66	0.580	0.1910	Yes
16	COL12A1	collagen type	69	0.577	0.2025	Yes
17	ECM2	extracellular m	80	0.569	0.2134	Yes
18	TPM1	tropomyosin 1	85	0.565	0.2246	Yes
19	FBLN1	fibulin 1 [Sour	94	0.560	0.2354	Yes
20	FBLN2	fibulin 2 [Sour	98	0.552	0.2464	Yes
21	TPM2	tropomyosin 2	111	0.547	0.2567	Yes
22	ID2	inhibitor of DN	112	0.544	0.2677	Yes
23	COL1A1	collagen type	117	0.543	0.2784	Yes
24	HTRA1	HtrA serine pe	133	0.534	0.2883	Yes
25	LOXL1	lysyl oxidase li	148	0.525	0.2981	Yes
26	DPYSL3	dihydropyrimid	153	0.523	0.3084	Yes
27	EFEMP2	EGF containin	157	0.521	0.3188	Yes
28	DCN	decorin [Sourc	159	0.520	0.3292	Yes
29	EDIL3	EGF like repea	171	0.512	0.3389	Yes
30	MXRA5	matrix remode	186	0.505	0.3482	Yes
31	MFAP5	microfibril assc	192	0.504	0.3581	Yes
32	SPARC	secreted prote	208	0.499	0.3673	Yes
33	MYL9	myosin light ch	228	0.492	0.3761	Yes
34	IGFBP4	insulin like gro	240	0.484	0.3852	Yes
35	ITGB1	integrin subun	253	0.479	0.3942	Yes
36	COL5A1	collagen type	259	0.478	0.4035	Yes
37	COL5A2	collagen type	270	0.473	0.4125	Yes
38	TAGLN	transgelin [Sou	302	0.459	0.4199	Yes
39	PMEPA1	"prostate trans	311	0.457	0.4287	Yes
40	SFRP4	secreted frizzle	332	0.452	0.4366	Yes
41	BASP1	brain abundar	358	0.444	0.4441	Yes
42	ACTA2	"actin alpha 2	361	0.443	0.4529	Yes
43	MYLK	myosin light ch	379	0.437	0.4607	Yes
44	THY1	Thy-1 cell surfa	383	0.435	0.4693	Yes
45	SLIT3	slit guidance li	402	0.430	0.4770	Yes
46	FBLN5	fibulin 5 [Sour	437	0.422	0.4835	Yes
47	FLNA	filamin A [Sour	455	0.417	0.4909	Yes
48	MSX1	msh homeobo	484	0.408	0.4975	Yes
49	LRRC15	leucine rich re	514	0.401	0.5039	Yes
50	LRP1	LDL receptor r	530	0.396	0.5110	Yes
51	THBS1	thrombospond	553	0.391	0.5176	Yes
52	NNMT	nicotinamide N	603	0.379	0.5223	Yes
53	GLIPR1	GLI pathogen	616	0.377	0.5292	Yes
54	FERMT2	fermitin family	627	0.375	0.5362	Yes
55	LAMA2	laminin subuni	636	0.372	0.5432	Yes
56	MATN3	matrilin 3 [Sou	647	0.370	0.5501	Yes
57	SCG2	secretogranin	664	0.365	0.5566	Yes
58	FOXC2	forkhead box	667	0.365	0.5638	Yes
59	GEM	GTP binding p	681	0.362	0.5703	Yes
60	ITGA5	integrin subun	726	0.353	0.5749	Yes
61	MATN2	matrilin 2 [Sou	729	0.352	0.5819	Yes
62	FMOD	fibromodulin [S	737	0.350	0.5885	Yes
63	GAS1	growth arrest s	763	0.346	0.5940	Yes
64	SPOCK1	"SPARC (oste	850	0.333	0.5957	Yes
65	VCAM1	vascular cell a	875	0.328	0.6009	Yes
66	FGF2	fibroblast grow	903	0.323	0.6058	Yes
67	CDH6	cadherin 6 [Sc	904	0.323	0.6123	Yes
68	VCAN	versican [Sour	927	0.318	0.6174	Yes
69	MGP	matrix Gla prot	985	0.311	0.6204	Yes
70	VEGFC	vascular endo	1042	0.303	0.6232	Yes
71	ADAM12	ADAM metallo	1129	0.292	0.6240	Yes
72	COL8A2	collagen type	1169	0.288	0.6275	Yes
73	COMP	cartilage oligo	1189	0.286	0.6322	Yes

74	SLIT2	slit guidance li	1234	0.282	0.6353	Yes
75	ANPEP	"alanyl aminop	1253	0.280	0.6398	Yes
76	DAB2	DAB adaptor p	1291	0.275	0.6432	Yes
77	NT5E	5'-nucleotidase	1293	0.275	0.6487	Yes
78	TGFBR3	transforming g	1306	0.273	0.6535	Yes
79	SGCG	sarcoglycan g	1328	0.271	0.6577	Yes
80	GJA1	gap junction p	1419	0.261	0.6577	Yes
81	FZD8	frizzled class r	1482	0.256	0.6592	Yes
82	IL32	interleukin 32	1490	0.255	0.6639	Yes
83	GPX7	glutathione pe	1622	0.244	0.6611	Yes
84	IL15	interleukin 15	1654	0.241	0.6641	Yes
85	MMP14	matrix metallop	1672	0.238	0.6679	Yes
86	PTHLH	parathyroid hc	1697	0.236	0.6713	Yes
87	COL4A1	collagen type	1701	0.235	0.6758	Yes
88	CTHRC1	collagen triple	1757	0.231	0.6773	Yes
89	IL6	interleukin 6 [S	1915	0.220	0.6724	Yes
90	TGFBI	transforming g	1936	0.218	0.6756	Yes
91	COL16A1	collagen type	1965	0.217	0.6784	Yes
92	COL4A2	collagen type	1966	0.217	0.6827	Yes
93	NTM	neurotrimin [S	1981	0.215	0.6863	Yes
94	VIM	vimentin [Sour	1999	0.214	0.6896	Yes

Gene sets database hallmarks .all.v2023.2.Hs.symbols.gmt

**GEO**	**GSE65904**
**phenotype**	PRRX1
**ranked by**	pearson correlation

POSITIVELY CORRELATED

NAME	GS<br>follow link to MSigDB	GS DET/	SIZE	ES	NES	NOM p-v	FDR q-val	FWER p-	RANK A	LEADING EDGE
HALLMARK_EPITHELIAL_MESENCHYMAL_TRA	HALLMARK_EPITHELIAL_MESENCHYMAL_	see Deta	200	0,7932129	2,30739	0	0	0	2885	tags=72%, list=13%, signal=82%
HALLMARK_APICAL_JUNCTION	HALLMARK_APICAL_JUNCTION	Details ..	199	0,6036681	2,26113	0	0	0	3511	tags=47%, list=16%, signal=55%
HALLMARK_KRAS_SIGNALING_UP	HALLMARK_KRAS_SIGNALING_UP	Details ..	200	0,60389316	2,13405	0	0,00176945	0,004	3619	tags=48%, list=17%, signal=57%
HALLMARK_COAGULATION	HALLMARK_COAGULATION	Details ..	138	0,5808423	2,09214	0,002	0,00301252	0,007	3555	tags=42%, list=16%, signal=50%
HALLMARK_UV_RESPONSE_DN	HALLMARK_UV_RESPONSE_DN	Details ..	144	0,59470356	2,07084	0	0,00241001	0,007	2953	tags=45%, list=13%, signal=52%
HALLMARK_TGF_BETA_SIGNALING	HALLMARK_TGF_BETA_SIGNALING	Details ..	54	0,6487992	2,03864	0	0,00335457	0,013	2386	tags=46%, list=11%, signal=52%
HALLMARK_ANGIOGENESIS	HALLMARK_ANGIOGENESIS	Details ..	36	0,7373298	2,0116	0	0,0037166	0,016	2654	tags=64%, list=12%, signal=73%
HALLMARK_MYOGENESIS	HALLMARK_MYOGENESIS	Details ..	200	0,6192291	2,01059	0,002	0,00325202	0,016	5240	tags=67%, list=24%, signal=87%
HALLMARK_TNFA_SIGNALING_VIA_NFKB	HALLMARK_TNFA_SIGNALING_VIA_NFKB	Details ..	200	0,6438638	1,99791	0	0,00355062	0,02	4208	tags=58%, list=19%, signal=71%
HALLMARK_HYPOXIA	HALLMARK_HYPOXIA	Details ..	200	0,51865345	1,95228	0	0,00598576	0,034	2558	tags=38%, list=12%, signal=43%
HALLMARK_IL2_STAT5_SIGNALING	HALLMARK_IL2_STAT5_SIGNALING	Details ..	197	0,50441	1,90786	0	0,00856158	0,05	3861	tags=43%, list=18%, signal=51%
HALLMARK_APOPTOSIS	HALLMARK_APOPTOSIS	Details ..	161	0,4925371	1,90251	0,0019	0,00843215	0,054	4026	tags=43%, list=18%, signal=53%
HALLMARK_INFLAMMATORY_RESPONSE	HALLMARK_INFLAMMATORY_RESPONSE	Details ..	200	0,59221053	1,873	0,0121	0,01062021	0,07	4281	tags=54%, list=20%, signal=67%
HALLMARK_APICAL_SURFACE	HALLMARK_APICAL_SURFACE	Details ..	44	0,52433676	1,80861	0,0075	0,02024944	0,134	3582	tags=41%, list=16%, signal=49%
HALLMARK_XENOBIOTIC_METABOLISM	HALLMARK_XENOBIOTIC_METABOLISM	Details ..	199	0,43136808	1,80177	0,0076	0,02073458	0,139	2878	tags=30%, list=13%, signal=34%
HALLMARK_IL6_JAK_STAT3_SIGNALING	HALLMARK_IL6_JAK_STAT3_SIGNALING	Details ..	87	0,594533	1,80039	0,006	0,02000525	0,14	4036	tags=51%, list=18%, signal=62%
HALLMARK_COMPLEMENT	HALLMARK_COMPLEMENT	Details ..	200	0,5045737	1,7881	0,0098	0,02136236	0,152	4064	tags=37%, list=19%, signal=44%
HALLMARK_P53_PATHWAY	HALLMARK_P53_PATHWAY	Details ..	200	0,4251321	1,76687	0,0059	0,02452515	0,174	3633	tags=39%, list=17%, signal=46%
HALLMARK_ESTROGEN_RESPONSE_EARLY	HALLMARK_ESTROGEN_RESPONSE_EAR	Details ..	199	0,44635698	1,75932	0,0097	0,02488534	0,183	3916	tags=39%, list=18%, signal=47%
HALLMARK_ESTROGEN_RESPONSE_LATE	HALLMARK_ESTROGEN_RESPONSE_LAT	Details ..	200	0,4062812	1,68985	0,0186	0,03952039	0,27	3410	tags=34%, list=16%, signal=39%

NEGATIVELY CORRELATED

NAME	GS<br>follow link to MSigDB	GS DET/	SIZE	ES	NES	NOM p-v	FDR q-val	FWER p-	RANK A	LEADING EDGE
HALLMARK_OXIDATIVE_PHOSPHORYLATION	HALLMARK_OXIDATIVE_PHOSPHORYLATI	Details ...	200	-0,61569154	-1,92803	0,0101	0,02480643	0,038	3934	tags=60%, list=18%, signal=72%
HALLMARK_MYC_TARGETS_V1	HALLMARK_MYC_TARGETS_V1	Details ...	200	-0,52709097	-1,65044	0,0331	0,13972177	0,306	6302	tags=62%, list=29%, signal=86%
HALLMARK_MYC_TARGETS_V2	HALLMARK_MYC_TARGETS_V2	Details ...	57	-0,59754753	-1,64201	0,0466	0,09891047	0,321	3999	tags=54%, list=18%, signal=66%
HALLMARK_E2F_TARGETS	HALLMARK_E2F_TARGETS	Details ...	199	-0,54685044	-1,59928	0,0974	0,09630557	0,377	4719	tags=53%, list=22%, signal=67%
HALLMARK_DNA_REPAIR	HALLMARK_DNA_REPAIR	Details ...	150	-0,42461088	-1,55984	0,0559	0,09762172	0,429	4926	tags=43%, list=23%, signal=56%
HALLMARK_SPERMATOGENESIS	HALLMARK_SPERMATOGENESIS	Details ...	134	-0,31127104	-1,38244	0,0602	0,19449602	0,694	6875	tags=42%, list=31%, signal=61%
HALLMARK_G2M_CHECKPOINT	HALLMARK_G2M_CHECKPOINT	Details ...	200	-0,4181827	-1,318	0,2371	0,21839708	0,772	3074	tags=34%, list=14%, signal=39%
HALLMARK_MTORC1_SIGNALING	HALLMARK_MTORC1_SIGNALING	Details ...	200	-0,32142234	-1,26307	0,1853	0,23820195	0,836	4378	tags=33%, list=20%, signal=41%
HALLMARK_UNFOLDED_PROTEIN_RESPONSE	HALLMARK_UNFOLDED_PROTEIN_RESPO	Details ...	113	-0,27741578	-1,09025	0,3433	0,39170173	0,96	4430	tags=27%, list=20%, signal=34%
HALLMARK_FATTY_ACID_METABOLISM	HALLMARK_FATTY_ACID_METABOLISM	Details ...	158	-0,26034832	-1,00918	0,4291	0,45015064	0,988	3954	tags=30%, list=18%, signal=36%
HALLMARK_INTERFERON_ALPHA_RESPONSE	HALLMARK_INTERFERON_ALPHA_RESPO	Details ...	97	-0,18069965	-0,46087	0,913	0,98586863	1	6079	tags=31%, list=28%, signal=43%

Enrichment plot: HALLMARK_EPITHELIAL_MESENCHYMAL_TRANSITION
Profile of the Running ES Score & Positions of GeneSet Members on the Rank Ordered List

	SYMBOL	RANK IN	RANK METRI	RUNNING ES	CORE ENRICHM
1	PRRX1	0	1.000	0.0188	Yes
2	COL6A2	3	0.610	0.0302	Yes
3	FAP	7	0.569	0.0408	Yes
4	IGFBP4	8	0.562	0.0513	Yes
5	TPM4	19	0.539	0.0612	Yes
6	COL6A3	21	0.535	0.0712	Yes
7	CDH11	33	0.518	0.0806	Yes
8	FBN1	35	0.516	0.0902	Yes
9	COL5A1	38	0.515	0.0999	Yes
10	LOXL1	40	0.514	0.1095	Yes
11	HTRA1	42	0.510	0.1190	Yes
12	COL5A2	43	0.510	0.1286	Yes
13	NID2	47	0.505	0.1380	Yes
14	COL3A1	49	0.505	0.1474	Yes
15	PDGFRB	50	0.504	0.1569	Yes
16	COL1A1	53	0.502	0.1663	Yes
17	ITGA5	57	0.499	0.1756	Yes
18	MXRA5	58	0.498	0.1849	Yes
19	MMP2	60	0.496	0.1942	Yes
20	THY1	63	0.495	0.2034	Yes
21	PCOLCE	65	0.493	0.2127	Yes
22	VCAN	68	0.492	0.2219	Yes
23	FBLN1	80	0.485	0.2307	Yes
24	COL1A2	84	0.483	0.2396	Yes
25	COL4A1	87	0.482	0.2486	Yes
26	SLIT3	92	0.476	0.2574	Yes
27	COL8A2	93	0.476	0.2664	Yes
28	ANPEP	96	0.475	0.2752	Yes
29	NNMT	104	0.472	0.2839	Yes
30	LRP1	106	0.471	0.2927	Yes
31	SPARC	118	0.461	0.3011	Yes
32	COL4A2	120	0.460	0.3097	Yes
33	LUM	126	0.457	0.3181	Yes
34	MFAP5	132	0.455	0.3265	Yes
35	COMP	135	0.454	0.3350	Yes
36	BGN	139	0.450	0.3433	Yes
37	FMOD	141	0.449	0.3517	Yes
38	FSTL1	144	0.447	0.3601	Yes
39	PMEPA1	151	0.446	0.3683	Yes
40	NTM	153	0.445	0.3766	Yes
41	ECM2	166	0.441	0.3845	Yes
42	LAMA2	168	0.440	0.3928	Yes
43	NT5E	176	0.437	0.4008	Yes
44	MYL9	180	0.435	0.4088	Yes
45	TGFBI	183	0.434	0.4169	Yes
46	DPYSL3	187	0.433	0.4250	Yes
47	SFRP4	191	0.432	0.4330	Yes
48	CXCL12	194	0.432	0.4411	Yes
49	FBLN2	195	0.431	0.4492	Yes

50	DCN	196	0.431	0.4573	Yes
51	ELN	210	0.427	0.4649	Yes
52	LOX	212	0.426	0.4729	Yes
53	MMP3	214	0.426	0.4808	Yes
54	ITGB1	215	0.425	0.4888	Yes
55	TAGLN	219	0.424	0.4967	Yes
56	GEM	246	0.417	0.5038	Yes
57	THBS1	269	0.409	0.5108	Yes
58	GJA1	271	0.408	0.5184	Yes
59	SERPINE1	286	0.401	0.5255	Yes
60	CALD1	297	0.398	0.5327	Yes
61	COL5A3	320	0.389	0.5394	Yes
62	TPM1	330	0.386	0.5464	Yes
63	COL16A1	333	0.385	0.5536	Yes
64	LRRC15	353	0.380	0.5602	Yes
65	EFEMP2	381	0.375	0.5664	Yes
66	PLAUR	402	0.370	0.5728	Yes
67	GADD45A	407	0.368	0.5796	Yes
68	ACTA2	467	0.358	0.5846	Yes
69	THBS2	477	0.355	0.5910	Yes
70	BASP1	524	0.346	0.5961	Yes
71	FBLN5	630	0.329	0.5993	Yes
72	TIMP3	647	0.328	0.6050	Yes
73	VEGFC	651	0.327	0.6110	Yes
74	IL6	666	0.325	0.6167	Yes
75	GLIPR1	716	0.316	0.6212	Yes
76	PTX3	758	0.310	0.6259	Yes
77	GAS1	760	0.310	0.6316	Yes
78	FN1	782	0.307	0.6368	Yes
79	LAMC1	784	0.306	0.6425	Yes
80	MEST	804	0.304	0.6477	Yes
81	ABI3BP	805	0.304	0.6534	Yes
82	JUN	841	0.300	0.6580	Yes
83	MMP1	869	0.297	0.6628	Yes
84	ITGAV	891	0.294	0.6677	Yes
85	SLIT2	896	0.294	0.6731	Yes
86	SCG2	927	0.289	0.6777	Yes
87	FERMT2	936	0.287	0.6829	Yes
88	GPC1	946	0.286	0.6880	Yes
89	CRLF1	965	0.284	0.6928	Yes
90	MSX1	980	0.283	0.6977	Yes
91	LAMA3	985	0.282	0.7029	Yes
92	DAB2	1063	0.275	0.7058	Yes
93	GADD45B	1075	0.273	0.7106	Yes
94	COL7A1	1094	0.271	0.7152	Yes
95	SGCG	1120	0.269	0.7195	Yes
96	FOXC2	1121	0.269	0.7246	Yes
97	EDIL3	1124	0.269	0.7296	Yes
98	SPOCK1	1125	0.269	0.7346	Yes
99	RHOB	1273	0.256	0.7351	Yes
100	POSTN	1313	0.252	0.7387	Yes
101	MYLK	1385	0.246	0.7413	Yes
102	CTHRC1	1405	0.245	0.7453	Yes
103	TIMP1	1415	0.244	0.7497	Yes
104	PCOLCE2	1421	0.243	0.7541	Yes
105	SGCB	1432	0.242	0.7583	Yes
106	ITGA2	1454	0.240	0.7622	Yes
107	MATN3	1531	0.233	0.7644	Yes
108	WNT5A	1557	0.232	0.7680	Yes
109	PTHLH	1569	0.231	0.7721	Yes
110	LGALS1	1579	0.230	0.7761	Yes
111	ITGB5	1628	0.227	0.7790	Yes
112	GPX7	1698	0.223	0.7812	Yes
113	FLNA	1746	0.220	0.7839	Yes
114	IGFBP3	1750	0.220	0.7880	Yes
115	RGS4	1785	0.218	0.7911	Yes
116	WIPF1	1915	0.211	0.7913	Yes
117	TNFRSF12A	2045	0.203	0.7914	Yes
118	ECM1	2061	0.202	0.7947	Yes
119	MMP14	2106	0.199	0.7972	Yes
120	MGP	2120	0.199	0.8006	Yes
121	SDC1	2316	0.188	0.7984	Yes
122	VCAM1	2332	0.187	0.8015	Yes
123	PDLIM4	2334	0.187	0.8050	Yes
124	SERPINH1	2358	0.185	0.8078	Yes
125	CXCL6	2359	0.185	0.8113	Yes
126	PLOD2	2565	0.176	0.8086	Yes
127	TPM2	2608	0.175	0.8107	Yes
128	INHBA	2619	0.175	0.8137	Yes
129	TNC	2662	0.173	0.8157	Yes
130	PRSS2	2877	0.166	0.8126	Yes
131	FAS	2957	0.162	0.8134	Yes
132	COL11A1	3014	0.160	0.8148	Yes
133	TGFBR3	3025	0.160	0.8175	Yes
134	TNFRSF11B	3110	0.156	0.8180	Yes
135	ID2	3120	0.156	0.8207	Yes
136	BDNF	3255	0.152	0.8196	Yes
137	GREM1	3262	0.151	0.8223	Yes
138	PMP22	3342	0.148	0.8228	Yes
139	IGFBP2	3389	0.146	0.8242	Yes

140	FGF2	3416	0.146	0.8262	Yes
141	MATN2	3445	0.145	0.8281	Yes

Gene sets database hallmarks .all.v2023.2.Hs.symbols.gmt

**GEO**	**GSE116237**
**phenotype**	PRRX1
**ranked by**	pearson correlation

POSITIVELY CORRELATED

NAME	GS<br>follow link to MSigDB	GS DETAIL	SIZE	ES	NES	NOM p-v	FDR q-val	FWER p	RANK AT MA	LEADING EDGE
HALLMARK_UV_RESPONSE_DN	HALLMARK_UV_RESPONSE_DN	Details ...	138	0,2777	2,08272	0	0	0	2643	tags=39%, list=19%, signal=48%
HALLMARK_NOTCH_SIGNALING	HALLMARK_NOTCH_SIGNALING	Details ...	32	0,4058	2,0447	0	0	0	3324	tags=63%, list=24%, signal=82%
HALLMARK_EPITHELIAL_MESENCHYMAL_TRA	HALLMARK_EPITHELIAL_MESENCHYMAL_	see details	192	0,2553	1,9941	0	0,00113447	0,001	2624	tags=37%, list=19%, signal=45%
HALLMARK_TGF_BETA_SIGNALING	HALLMARK_TGF_BETA_SIGNALING	Details ...	53	0,3066	1,77527	0	0,00680512	0,009	2244	tags=36%, list=16%, signal=43%
HALLMARK_UV_RESPONSE_UP	HALLMARK_UV_RESPONSE_UP	Details ...	146	0,227	1,67869	0	0,01026529	0,02	2553	tags=33%, list=18%, signal=40%
HALLMARK_PI3K_AKT_MTOR_SIGNALING	HALLMARK_PI3K_AKT_MTOR_SIGNALING	Details ...	100	0,2463	1,67775	0	0,00855441	0,02	3168	tags=38%, list=23%, signal=49%
HALLMARK_HYPOXIA	HALLMARK_HYPOXIA	Details ...	181	0,2146	1,6717	0	0,0079401	0,021	2057	tags=28%, list=15%, signal=32%
HALLMARK_TNFA_SIGNALING_VIA_NFKB	HALLMARK_TNFA_SIGNALING_VIA_NFKB	Details ...	180	0,193	1,49256	0	0,04011212	0,117	2025	tags=27%, list=15%, signal=31%
HALLMARK_APICAL_JUNCTION	HALLMARK_APICAL_JUNCTION	Details ...	166	0,1883	1,48423	0,012	0,02789815	0,124	3101	tags=33%, list=22%, signal=42%

NEGATIVELY CORRELATED

NAME	GS<br>follow link to MSigDB	GS DETAIL	SIZE	ES	NES	NOM p-v	FDR q-val	FWER p	RANK AT MA	LEADING EDGE
HALLMARK_MYC_TARGETS_V2	HALLMARK_MYC_TARGETS_V2	Details ...	57	-0,474	-2,2539	0	0	0	4182	tags=58%, list=30%, signal=83%
HALLMARK_OXIDATIVE_PHOSPHORYLATION	HALLMARK_OXIDATIVE_PHOSPHORYLATI	Details ...	185	-0,349	-2,0747	0	0,0003587	0,001	3704	tags=49%, list=27%, signal=66%
HALLMARK_INTERFERON_ALPHA_RESPONSE	HALLMARK_INTERFERON_ALPHA_RESPO	Details ...	90	-0,338	-1,7686	0,002	0,01199789	0,046	5061	tags=59%, list=37%, signal=92%
HALLMARK_MYC_TARGETS_V1	HALLMARK_MYC_TARGETS_V1	Details ...	194	-0,297	-1,768	0	0,00919485	0,047	3928	tags=46%, list=28%, signal=63%
HALLMARK_DNA_REPAIR	HALLMARK_DNA_REPAIR	Details ...	149	-0,302	-1,7536	0	0,00914847	0,058	4974	tags=52%, list=36%, signal=80%
HALLMARK_FATTY_ACID_METABOLISM	HALLMARK_FATTY_ACID_METABOLISM	Details ...	142	-0,282	-1,6255	0,004	0,03065707	0,215	3704	tags=42%, list=27%, signal=56%
HALLMARK_CHOLESTEROL_HOMEOSTASIS	HALLMARK_CHOLESTEROL_HOMEOSTAS	Details ...	70	-0,311	-1,5627	0,017	0,04973715	0,387	3418	tags=44%, list=25%, signal=59%
HALLMARK_E2F_TARGETS	HALLMARK_E2F_TARGETS	Details ...	196	-0,257	-1,5294	0,01	0,05816822	0,473	5780	tags=53%, list=42%, signal=90%
HALLMARK_UNFOLDED_PROTEIN_RESPONSE	HALLMARK_UNFOLDED_PROTEIN_RESPO	Details ...	105	-0,255	-1,3888	0,054	0,17326546	0,887	3287	tags=35%, list=24%, signal=46%
HALLMARK_ANDROGEN_RESPONSE	HALLMARK_ANDROGEN_RESPONSE	Details ...	94	-0,251	-1,3358	0,076	0,23554717	0,958	3439	tags=34%, list=25%, signal=45%
HALLMARK_IL6_JAK_STAT3_SIGNALING	HALLMARK_IL6_JAK_STAT3_SIGNALING	Details ...	62	-0,272	-1,3237	0,102	0,23537213	0,975	4862	tags=47%, list=35%, signal=72%
HALLMARK_INTERFERON_GAMMA_RESPONSE	HALLMARK_INTERFERON_GAMMA_RESPO	Details ...	172	-0,218	-1,2794	0,097	0,29992983	0,995	6039	tags=56%, list=44%, signal=99%
HALLMARK_P53_PATHWAY	HALLMARK_P53_PATHWAY	Details ...	187	-0,192	-1,1399	0,233	0,64386934	1	5617	tags=47%, list=41%, signal=78%
HALLMARK_APOPTOSIS	HALLMARK_APOPTOSIS	Details ...	147	-0,198	-1,1304	0,253	0,6296302	1	4106	tags=37%, list=30%, signal=52%
HALLMARK_COAGULATION	HALLMARK_COAGULATION	Details ...	107	-0,207	-1,1254	0,266	0,6026569	1	4254	tags=36%, list=31%, signal=52%
HALLMARK_G2M_CHECKPOINT	HALLMARK_G2M_CHECKPOINT	Details ...	189	-0,187	-1,1167	0,278	0,58987397	1	5780	tags=47%, list=42%, signal=79%
HALLMARK_MTORC1_SIGNALING	HALLMARK_MTORC1_SIGNALING	Details ...	197	-0,188	-1,116	0,263	0,556862	1	5780	tags=52%, list=42%, signal=88%
HALLMARK_ADIPOGENESIS	HALLMARK_ADIPOGENESIS	Details ...	186	-0,175	-1,0229	0,426	0,79975045	1	3676	tags=33%, list=27%, signal=45%
HALLMARK_REACTIVE_OXYGEN_SPECIES_PA	HALLMARK_REACTIVE_OXYGEN_SPECIE	Details ...	46	-0,22	-1,007	0,454	0,80726767	1	2686	tags=33%, list=19%, signal=40%
HALLMARK_HEDGEHOG_SIGNALING	HALLMARK_HEDGEHOG_SIGNALING	Details ...	32	-0,235	-0,9741	0,515	0,85935557	1	1998	tags=25%, list=14%, signal=29%
HALLMARK_PROTEIN_SECRETION	HALLMARK_PROTEIN_SECRETION		92	-0,183	-0,9718	0,518	0,8246497	1	3779	tags=36%, list=27%, signal=49%
HALLMARK_COMPLEMENT	HALLMARK_COMPLEMENT		169	-0,166	-0,9687	0,53	0,79516894	1	4364	tags=35%, list=32%, signal=50%
HALLMARK_APICAL_SURFACE	HALLMARK_APICAL_SURFACE		37	-0,214	-0,9319	0,554	0,85270643	1	7147	tags=62%, list=52%, signal=128
HALLMARK_PEROXISOME	HALLMARK_PEROXISOME		94	-0,175	-0,9311	0,584	0,81893563	1	3165	tags=30%, list=23%, signal=38%
HALLMARK_KRAS_SIGNALING_UP	HALLMARK_KRAS_SIGNALING_UP		157	-0,16	-0,9239	0,644	0,8030762	1	2768	tags=22%, list=20%, signal=28%
HALLMARK_BILE_ACID_METABOLISM	HALLMARK_BILE_ACID_METABOLISM		98	-0,167	-0,8911	0,664	0,8431417	1	5342	tags=40%, list=39%, signal=64%
HALLMARK_PANCREAS_BETA_CELLS	HALLMARK_PANCREAS_BETA_CELLS		22	-0,23	-0,8756	0,612	0,84361595	1	2269	tags=23%, list=16%, signal=27%
HALLMARK_IL2_STAT5_SIGNALING	HALLMARK_IL2_STAT5_SIGNALING		168	-0,143	-0,8382	0,761	0,88619363	1	4787	tags=36%, list=35%, signal=55%
HALLMARK_ESTROGEN_RESPONSE_LATE	HALLMARK_ESTROGEN_RESPONSE_LATE		174	-0,129	-0,7539	0,892	0,99158174	1	4521	tags=32%, list=33%, signal=46%
HALLMARK_HEME_METABOLISM	HALLMARK_HEME_METABOLISM		174	-0,126	-0,7388	0,893	0,9767914	1	2763	tags=21%, list=20%, signal=26%
HALLMARK_ESTROGEN_RESPONSE_EARLY	HALLMARK_ESTROGEN_RESPONSE_EARLY		177	-0,123	-0,7325	0,92	0,9532994	1	4637	tags=33%, list=34%, signal=49%
HALLMARK_MITOTIC_SPINDLE	HALLMARK_MITOTIC_SPINDLE		195	-0,111	-0,6711	0,97	0,9781008	1	5780	tags=39%, list=42%, signal=66%
HALLMARK_SPERMATOGENESIS	HALLMARK_SPERMATOGENESIS		92	-0,103	-0,5508	0,992	0,99312323	1	3863	tags=25%, list=28%, signal=34%

Enrichment plot: HALLMARK_EPITHELIAL_MESENCHYMAL_TRANSITION
Profile of the Running ES Score & Positions of GeneSet Members on the Rank Ordered List

	SYMBOL	TITLE	K IN GENE	METRIC	JNNING I	ENRICHMENT
1	PRRX1	paired rela	0	1.000	0.0077	Yes
2	BGN	biglycan [S	26	0.986	0.0136	Yes
3	FBLN5	fibulin 5 [S	40	0.981	0.0203	Yes
4	LOXL1	lysyl oxida	53	0.978	0.0270	Yes
5	EFEMP2	EGF conta	71	0.975	0.0334	Yes
6	WNT5A	Wnt family	74	0.974	0.0408	Yes
7	THBS2	thrombosp	89	0.970	0.0473	Yes
8	MMP14	matrix met	101	0.967	0.0540	Yes
9	FSTL1	follistatin li	105	0.967	0.0613	Yes
10	MFAP5	microfibril a	127	0.963	0.0673	Yes
11	MYL9	myosin ligh	184	0.953	0.0708	Yes
12	FUCA1	alpha-L-fuc	203	0.950	0.0769	Yes
13	CRLF1	cytokine re	209	0.949	0.0839	Yes
14	MSX1	msh home	212	0.949	0.0911	Yes
15	INHBA	inhibin sub	219	0.947	0.0980	Yes
16	PTX3	pentraxin 3	226	0.947	0.1049	Yes
17	PMEPA1	prostate tra	381	0.924	0.1015	Yes
18	COL6A2	collagen ty	410	0.920	0.1066	Yes
19	FLNA	filamin A [S	413	0.920	0.1136	Yes
20	SDC1	syndecan	427	0.918	0.1198	Yes
21	MAGEE1	MAGE fam	432	0.917	0.1266	Yes
22	TGFBR3	transformin	442	0.916	0.1331	Yes
23	CCN1	cellular cor	490	0.909	0.1369	Yes
24	FERMT2	FERM dom	536	0.902	0.1407	Yes
25	HTRA1	HtrA serine	550	0.901	0.1468	Yes
26	COL12A1	collagen ty	555	0.900	0.1535	Yes
27	NID2	nidogen 2	576	0.898	0.1591	Yes
28	PCOLCE	procollage	621	0.890	0.1629	Yes
29	TNFRSF11B	TNF recep	622	0.890	0.1698	Yes
30	CAPG	capping ac	646	0.887	0.1751	Yes
31	JUN	Jun proto-o	674	0.884	0.1800	Yes
32	VEGFA	vascular en	675	0.884	0.1869	Yes
33	LOX	lysyl oxida	686	0.882	0.1930	Yes
34	VCAM1	vascular ce	761	0.872	0.1946	Yes
35	FAP	fibroblast a	803	0.868	0.1985	Yes
36	COL6A3	collagen ty	917	0.850	0.1974	Yes
37	SNAI2	snail family	1004	0.837	0.1980	Yes
38	CXCL6	C-X-C mot	1057	0.829	0.2008	Yes
39	DPYSL3	dihydropyr	1111	0.819	0.2035	Yes
40	CDH11	cadherin 1	1195	0.806	0.2040	Yes
41	BASP1	brain abun	1229	0.801	0.2080	Yes
42	SLIT3	slit guidanc	1234	0.800	0.2139	Yes
43	FBLN2	fibulin 2 [S	1240	0.799	0.2197	Yes
44	CDH2	cadherin 2	1361	0.779	0.2175	Yes
45	BDNF	brain deriv	1458	0.763	0.2168	Yes
46	EMP3	epithelial n	1507	0.754	0.2193	Yes
47	NNMT	nicotinamid	1545	0.749	0.2226	Yes
48	FBN1	fibrillin 1 [S	1547	0.749	0.2283	Yes
49	BMP1	bone morp	1606	0.739	0.2300	Yes
50	ECM2	extracellula	1610	0.738	0.2355	Yes
51	DAB2	DAB adapt	1730	0.716	0.2329	Yes
52	TPM2	tropomyos	1770	0.708	0.2357	Yes
53	TPM1	tropomyos	1845	0.695	0.2360	Yes
54	COL3A1	collagen ty	1851	0.694	0.2410	Yes
55	COL5A1	collagen ty	1946	0.673	0.2398	Yes
