# Supplementary material for: PRRX1 silencing is required for metastatic outgrowth in melanoma and is an independent prognostic of reduced survival in patients
Source: Mol Oncol. 2024 Jul 8;18(10):2471–94. doi: 10.1002/1878-0261.13688 (PMC11459042; doi:10.1002/1878-0261.13688)
Supplement: Supplementary file 8 — Table S10. KRAS _UP genes from pre‐ranked GSEA in TCGA‐SKCM (PRRX1 high vs PRRX1 low) and MAPK related Hallmarks Gene sets from GSEA (PRRX1 Pearson correlation) in GSE22155; GSE65904; GSE116237. [file MOL2-18-2471-s002.pdf]

Supplementary Table 10: Gene Set Members on the Rank ordered list

Enrichment plot HALLMARK_KRAS_SIGNALING_UP

PRE-RANKED GSEA TCGA-SKCM (INVASIVE vs NON INVASIVE GENES)

Enrichment Score (ES)	0.5751757
Normalized Enrichment Score (NES)	2.45718
Nominal p-value	0.0
FDR q-value	0.0
FWER p-Value	0.0

	SYMBOL	RANK IN GENE LIST	RANK METRIC SCORE	RUNNING ES	RE ENRICHMENT
1	INHBA	12	7.204	0.0162	Yes
2	NRP1	16	7.013	0.0325	Yes
3	PLAU	32	6.748	0.0475	Yes
4	GUCY1A1	37	6.632	0.0628	Yes
5	BMP2	69	6.098	0.0754	Yes
6	CPE	86	5.896	0.0884	Yes
7	SPON1	91	5.794	0.1018	Yes
8	**PRRX1**	98	5.729	0.1148	Yes
9	ANO1	126	5.480	0.1262	Yes
10	F13A1	134	5.409	0.1385	Yes
11	PRDM1	162	5.204	0.1493	Yes
12	EPHB2	166	5.177	0.1612	Yes
13	TFPI	208	5.006	0.1708	Yes
14	PECAM1	223	4.943	0.1816	Yes
15	ANKH	262	4.734	0.1907	Yes
16	F2RL1	306	4.589	0.1991	Yes
17	MMP11	362	4.416	0.2066	Yes
18	ADGRL4	388	4.361	0.2154	Yes
19	VWA5A	405	4.314	0.2247	Yes
20	MAFB	424	4.275	0.2338	Yes
21	CMKLR1	477	4.140	0.2407	Yes
22	HBEGF	484	4.125	0.2500	Yes
23	MPZL2	508	4.068	0.2583	Yes
24	CLEC4A	545	4.001	0.2658	Yes
25	RGS16	589	3.944	0.2727	Yes
26	FLT4	725	3.740	0.2743	Yes
27	PLVAP	737	3.731	0.2825	Yes
28	CA2	787	3.667	0.2885	Yes
29	IL1RL2	829	3.611	0.2947	Yes
30	ACE	959	3.467	0.2960	Yes
31	NGF	1016	3.377	0.3009	Yes
32	PLAUR	1020	3.370	0.3087	Yes
33	SPARCL1	1031	3.360	0.3160	Yes
34	MAP7	1084	3.304	0.3210	Yes
35	LAT2	1085	3.304	0.3287	Yes
36	PEG3	1096	3.297	0.3359	Yes
37	ALDH1A3	1097	3.294	0.3436	Yes
38	TNFRSF1B	1122	3.262	0.3500	Yes
39	ITGA2	1130	3.258	0.3572	Yes
40	IL1B	1169	3.216	0.3627	Yes
41	MALL	1222	3.178	0.3674	Yes
42	KLF4	1226	3.175	0.3747	Yes
43	ITGBL1	1233	3.161	0.3818	Yes
44	CFH	1262	3.139	0.3876	Yes
45	C3AR1	1304	3.100	0.3927	Yes
46	TMEM158	1364	3.052	0.3967	Yes
47	TMEM176A	1379	3.037	0.4031	Yes
48	MMP10	1440	2.979	0.4069	Yes
49	TMEM100	1587	2.864	0.4058	Yes
50	IGF2	1599	2.855	0.4119	Yes
51	TMEM176B	1639	2.822	0.4165	Yes
52	CCND2	1650	2.818	0.4225	Yes
53	KIF5C	1683	2.793	0.4274	Yes
54	BTC	1694	2.785	0.4334	Yes
55	MMP9	1728	2.766	0.4381	Yes
56	PDCD1LG2	1742	2.760	0.4439	Yes
57	IGFBP3	1752	2.752	0.4498	Yes
58	LIF	1754	2.751	0.4562	Yes

59	IL7R	1776	2.739	0.4615	Yes
60	TSPAN1	1786	2.729	0.4674	Yes
61	GNG11	1815	2.705	0.4723	Yes
62	FCER1G	1851	2.687	0.4767	Yes
63	SCG5	1881	2.671	0.4814	Yes
64	JUP	1906	2.654	0.4864	Yes
65	ADAM17	2083	2.551	0.4830	Yes
66	TLR8	2114	2.533	0.4873	Yes
67	NAP1L2	2128	2.529	0.4926	Yes
68	CXCR4	2131	2.527	0.4984	Yes
69	SERPINA3	2163	2.512	0.5026	Yes
70	ANGPTL4	2178	2.503	0.5077	Yes
71	GLRX	2280	2.440	0.5081	Yes
72	ENG	2318	2.425	0.5118	Yes
73	ITGB2	2330	2.419	0.5169	Yes
74	FUCA1	2337	2.417	0.5222	Yes
75	ADGRA2	2373	2.400	0.5260	Yes
76	TSPAN13	2390	2.393	0.5307	Yes
77	LAPTM5	2540	2.317	0.5282	Yes
78	G0S2	2564	2.304	0.5324	Yes
79	AKAP12	2579	2.298	0.5371	Yes
80	IL10RA	2665	2.259	0.5378	Yes
81	SLPI	2788	2.204	0.5365	Yes
82	DOCK2	2883	2.166	0.5366	Yes
83	PTGS2	2906	2.154	0.5405	Yes
84	IRF8	2961	2.128	0.5426	Yes
85	TRAF1	3006	2.113	0.5452	Yes
86	ARG1	3104	2.075	0.5449	Yes
87	SPP1	3403	1.957	0.5337	Yes
88	APOD	3435	1.946	0.5366	Yes
89	SCN1B	3559	1.900	0.5345	Yes
90	ID2	3634	1.869	0.5349	Yes
91	ST6GAL1	3663	1.859	0.5378	Yes
92	ADAM8	3672	1.856	0.5417	Yes
93	WNT7A	3779	1.814	0.5403	Yes
94	CCL20	3797	1.805	0.5437	Yes
95	CIDEA	3853	1.781	0.5449	Yes
96	EREG	3945	1.761	0.5442	Yes
97	LCP1	4106	1.702	0.5397	Yes
98	LY96	4145	1.687	0.5416	Yes
99	HSD11B1	4149	1.686	0.5454	Yes
100	HKDC1	4152	1.685	0.5493	Yes
101	IKZF1	4159	1.684	0.5529	Yes
102	ETV1	4241	1.656	0.5525	Yes
103	MYCN	4256	1.651	0.5556	Yes
104	KCNN4	4279	1.641	0.5583	Yes
105	AMMECR1	4320	1.628	0.5599	Yes
106	PLEK2	4410	1.595	0.5589	Yes
107	CROT	4442	1.583	0.5610	Yes
108	GFPT2	4446	1.582	0.5646	Yes
109	HOXD11	4491	1.567	0.5659	Yes
110	TSPAN7	4538	1.549	0.5671	Yes
111	ADAMDEC1	4559	1.540	0.5696	Yes
112	IL33	4614	1.520	0.5703	Yes
113	CBL	4647	1.511	0.5721	Yes
114	ABCB1	4700	1.490	0.5729	Yes
115	SATB1	4723	1.482	0.5752	Yes
